# Neural, cognitive and psychopathological signatures of a prosocial or delinquent peer environment during early adolescence

**DOI:** 10.1016/j.dcn.2025.101566

**Published:** 2025-05-08

**Authors:** Yu Liu, Songjun Peng, Xinran Wu, Zhaowen Liu, Zhengxu Lian, Huaxin Fan, Nanyu Kuang, Xinrui Gu, Senyou Yang, Yechen Hu, Xi Jiang, Yufeng Zhang, Wei Cheng, Jianfeng Feng, Barbara J. Sahakian, Xingming Zhao, Trevor W. Robbins, Benjamin Becker, Jie Zhang

**Affiliations:** a Institute of Science and Technology for Brain-Inspired Intelligence, Fudan University, Shanghai, China; b Key Laboratory of Computational Neuroscience and Brain Inspired Intelligence, Fudan University, Ministry of Education, Shanghai, China; c School of Computer Science, Northwestern Polytechnical University, Xi’an, Shaanxi, China; d Sino-European School of Technology, Shanghai University, Shanghai, China; e The Center of Psychosomatic Medicine, Sichuan Provincial Center for Mental Health, Sichuan Provincial People's Hospital, University of Electronic Science and Technology of China, Chengdu, China; f Chinese language and literature, Fudan University, Shanghai, China; g Department of Computer Science, University of Warwick, Coventry, United Kingdom; h Behavioural and Clinical Neuroscience Institute, University of Cambridge, Cambridge, United Kingdom; i Department of Psychiatry, University of Cambridge School of Clinical Medicine, Cambridge, United Kingdom; j Department of Psychology, University of Cambridge, Cambridge, United Kingdom; k State Key Laboratory of Brain and Cognitive Sciences, The University of Hong Kong, Hong Kong; l Department of Psychology, The University of Hong Kong, Hong Kong

**Keywords:** Peer environments, Adolescent development, Behavioral problems, Brain structure, Functional connectivity

## Abstract

Adolescence is a critical period for brain development, yet the impact of peer environments on brain structure, cognition, and psychopathology remains poorly understood. Here, we capitalized on data from 7806 adolescents (age = 12.02 ± 0.67) from the Adolescent Brain Cognitive Development (ABCD) study, to determine associations between two distinct peer environments (proportion of prosocial or delinquent friends) and the structural and functional architecture of the brain, cognition, as well as behavioral and emotional dysregulation. A higher proportion of prosocial friends was associated with fewer behavioral problems and larger fronto-cingulate and striatal regions. In contrast, a higher proportion of delinquent friends was linked to increased behavioral problems, lower neurocognitive performance, and decreased functional connectivity in the default-mode and fronto-striato-limbic circuits, which spatially overlapped with external dopamine density maps. Moreover, the associations between prosocial friends and behaviors were mediated by brain volumes (e.g., pallidum), while the associations between delinquent friends and behaviors were primarily mediated by fronto-striato-limbic connectivity. Prosocial friends also attenuated the development of internalizing problems, whereas delinquent friends promoted externalizing symptoms. These findings underscore the profound influence of peer environments on adolescent brain development and mental health, highlighting the need for early interventions to promote resilience and healthy neuro-maturation.

## 1 Introduction

Adolescence is a critical developmental period during which the brain undergoes critical changes (Tooley et al., 2021, Blakemore and Mills, 2014). Peer influence plays a particularly important role during this period (Foulkes and Blakemore, 2018, Ragelienė, 2016). The transition from childhood into adolescence is not only characterized by an increasing amount of time spent with peers (Larson and Richards, 1991), but also the development of increasingly complex social networks with peers (Lamblin et al., 2017). However, converging lines of evidence suggest that peers can influence adolescent development in both positive and negative ways (Andrews et al., 2020, Hoffman et al., 2006, Güroğlu, 2022, Foulkes and Blakemore, 2021).

Adolescents with higher-quality friendships demonstrated greater psychological resilience (van Harmelen et al., 2017, van Harmelen et al., 2021) and demonstrated a weakened association between negative parenting practices and subsequent antisocial behavior (Lansford et al., 2003). Adolescents who received peer support reported a reduction in depressive symptoms (Roach, 2018). By contrast, exposure to peer bullying and victimization in adolescence has been associated with greater psychopathology, including psychotic symptoms (Boden et al., 2016) and depressive symptoms (Kaltiala-Heino et al., 2010, Machmutow et al., 2012), as well as more risky behavior (e.g., drug use) (Sullivan et al., 2006). Furthermore, a recent study revealed that adolescents face a higher risk of mental disorder diagnoses when more than one classmate with mental disorders (Alho et al., 2024). These findings suggest that peer influence may amplify negative as well as positive behavioral tendencies. Additionally, even the presence of peers can influence adolescent behavior; for instance, adolescents are more likely to be involved in accidents when driving with passengers (Chen et al., 2000).

The neural basis of peer influence has been investigated mostly in social-emotional and reward-processing contexts (Burnett et al., 2011, Andrews et al., 2021). Adolescents tend to make riskier decisions in the presence of peers, which is associated with increased activity in reward circuitry including the ventral striatum and orbitofrontal cortex (Chein et al., 2011). Peer victimization during adolescence is associated with decreased putamen volume and generalized anxiety (Quinlan et al., 2020), suggesting that negative peer experiences during this period might serve as a stressor that can imprint on brain structure, i.e. decreased regional brain volume (Fuhrmann et al., 2015). More prosocial behavior resulting from peer influence has been linked to heightened activity in brain systems related to self-referential processing, i.e. the temporal-parietal junction and medial prefrontal cortex (Van Hoorn et al., 2016). Additionally, studies have demonstrated that friends exhibited greater neural similarity than non-friends during both resting states (Hyon et al., 2020) and movie viewing tasks (Parkinson et al., 2018).

However, prior research primarily focused on the influences of prosocial or delinquent friends separately, leaving the specific brain pathways through which different peer environments affect cognition and behavior, and the underlying neural mechanisms unclear. For example, exposure to chronic stress has been linked with accelerated brain maturation (Tooley et al., 2021, Wong et al., 2023). Negative peer status has been linked to long-term stress, including relational victimization (Lafko et al., 2015). Given that adolescent brain maturation is accompanied by regional-specific reductions in brain structure (e.g., volumes) (Bethlehem et al., 2022), the negative friends as a stressful environmental factor may have accelerated structural changes, while the positive friends as a protective factor buffering adolescent stress exposure may have promoted normal brain maturation trajectories reflected in larger volumes. Moreover, neurotransmitter receptors are expected to regulate cortical development (Grewen et al., 2014, Lotter et al., 2024) and close friends in childhood have been shown to exhibit greater similarity in brain volumes of social brain regions (D’Onofrio et al., 2021) (e.g., temporal-parietal junction, medial prefrontal cortex), which are critical for understanding others during adolescence (Blakemore, 2008). Given that negative peer status correlates with relational victimization (Lafko et al., 2015) and chronic stress is associated with profound and enduring adverse effects on brain structure and neurotransmitter systems (Sandi and Haller, 2015). Therefore, investigating the interplay between peer environments, brain signatures, and neurotransmitter changes may provide critical insights into how peer environments modulate adolescent brain development through neurodevelopmental pathways. Additionally, substantial brain development occurs during adolescence, including mentalizing and resistance to peer influence (Andrews et al., 2021). Thus understanding how peers shape behaviors through neural mechanisms is also important. Finally, friendship-based interventions often focus on how friends being trained can support members of their social circle who may present with mental issues (Manchanda et al., 2023). However, these interventions frequently overlook the variability in outcomes among different peer environments. Therefore, to potentially enhance the effectiveness of clinical strategies, it is essential to examine longitudinal associations between peer environments, behavioral problems, and mental health.

Against this background, we provide a systematic characterization of the influence of peer environments (operationalized by the proportion of prosocial or delinquent friends), across several different behavioral and neurobiological levels utilizing the Adolescent Brain and Cognitive Development (ABCD) database release 5.1 (https://abcdstudy.org) (Casey et al., 2018). Utilizing data from 7806 adolescents (age = 12.02 ± 0.67) at the 2-year follow-up (2YFU), the research examines associations between peer environments and adolescent behaviors, psychiatric symptoms, neurocognitive performance, structural brain metrics (volume, cortical thickness, surface area), as well as resting-state functional connectivity (RSFC).

**Fig. 1:**
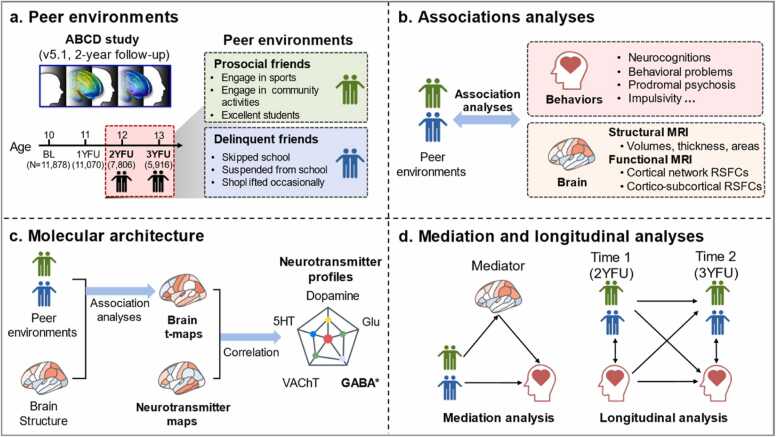
Overview of the study design. (a) 7806 adolescents at 2-year follow-up (age = 12.02 ± 0.67) in ABCD 5.1 release were used in the analyses. Peer environments are represented by the proportions of prosocial and delinquent peers, respectively. (b) Association analyses were employed to investigate the associations between peer environments (prosocial and delinquent friends), behavioral, and brain indices. (c) To understand the molecular architecture underlying peer influences, associations between brain topographical patterns (correlation between peer environments and regional cortical volumes) and the densities of expression of different neurotransmitter receptors were calculated. (d) Mediation analysis was used to test whether brain measures mediate the relationships between peer environments and behavioral/mental health indices. The cross-lagged panel models were used to test the longitudinal relationship (time 1 = 2YFU, time 2 = 3YFU) between peer environments and future behavior indices. Abbreviation: BL = baseline; 1YFU = 1-year follow-up; 2YFU = 2-year follow-up; 3YFU = 3-year follow-up; RSFC = resting-state functional connectivity.

We hypothesized that adolescents with a higher proportion of prosocial friends would exhibit fewer behavioral/psychiatric problems and higher cognitive ability, neurally reflected by positive associations with structural brain indices (increased volume in social brain regions, e.g., medial prefrontal cortex). Conversely, a higher proportion of delinquent friends would associate with more behavioral and emotional problems (including at follow-up) and opposing brain structural manifestations (e.g., volume in social brain regions, e.g., medial prefrontal cortex) and dysregulation in the functional network (e.g, default mode network, which is often linked with mental problems (Scalabrini et al., 2020); Tse et al., 2024). To further understand the molecular architecture of the neural circuits related to the peer environments, we explored the relationships between the peer environments, brain signatures, and neurotransmitter density. Additionally, we conducted mediation analyses to investigate how brain variations mediate the association between peer environments and cognitive as well as psychopathological outcomes at the behavioral level. Finally, using 3-year follow-up (3YFU) data (age = 12.92 ± 0.65), we explore the potential longitudinal associations of peer environments on future behavioral developmental trajectories, hypothesizing that prosocial friends would reduce behavioral problems, while delinquent friends would exacerbate them. See Fig. 1 for the overall study workflow.

## 2 Methods and materials

### 2.1 Participants

Data from the ABCD project of the National Institute of Mental Health National Data Archive (NDA) release 5.1, included 11,875 youths from 21 sites in the United States recruited at baseline. Due to data quality control and missing values, participant numbers varied across analyses to balance strict inclusion criteria with statistical power (Supplementary Table 1). Detailed inclusion criteria are provided in Supplementary Fig. 1. For cross-sectional analyses at 2YFU, participants ranging from 5916 to 7806 at 2YFU were included. The longitudinal analysis included 5916 participants with data from both 2YFU (age = 12.02 ± 0.67) and 3YFU (age = 12.92 ± 0.65). Parents provided full written informed consent, and children provided assent according to protocols approved by the institutional review board (IRB).

### 2.2 Peer environments

Peer environments were characterized by the prosocial friend index (PFI) and delinquent friend index (DFI) obtained from the youth peer behavior profile inventory (ce_y_pbp, first collected at 2YFU). A total of 6 items (scored 1–5 points) were used to measure involvement with prosocial (3 items) or delinquent friends (3 items), see Fig. 1a. For example, the proportion of friends who are excellent students (prosocial), or friends who have played truant from school (delinquent). The scores for 3 prosocial items and 3 delinquent items were respectively summed into the PFI and DFI. Adolescents with higher PFI or DFI reflect the strengths/proportion of the respective friend environment exposure but not the direct social behaviors, respectively.

### 2.3 Behavioral and mental health measurements

A total of 57 summary items describing cognition, behavioral problems, mental health, and adverse peer experience at the 2YFU assessments were used (Supplementary Table 2). These include 3 neurocognition items, 20 behavioral problem items assessed by the parent-reported ABCD child behavior checklist (CBCL) scores, and 34 mental health items of adolescents assessed by the self-report and parent-report questionnaires. The 34 mental health scores include (a) 8 parent-report summary scores including subsyndromal mania (1 item) and life events (7 items), and (b) 26 self-report summary scores including life events (6 items), prodromal psychosis levels (2 items), impulsivity (5 items), inhibition and reward-seeking (7 items), and adverse peer experiences (6 items).

### 2.4 Multimodal neuroimaging data and preprocessing

We used post-processed structural MRI and resting-state functional connectivity MRI data of the ABCD 5.1 release at 2YFU. Adolescents who passed the quality control were included in the imaging-related analyses. Imaging pre-processing and quality control steps have been described in previous studies (Hagler et al., 2019).

For T_1_w structural images, the Desikan atlas (Desikan et al., 2006) was used to segment the cortex into 68 regions, and three cortical metrics (brain volume, cortical thickness, and surface area) were used for each. Fischl's atlas (Fischl et al., 2002) was utilized to delineate the volume of 16 subcortical regions (the bilateral thalamus, caudate, putamen, pallidum, hippocampus, amygdala, accumbens, and ventral diencephalon).

For resting-state fMRI, post-processed time series were resampled onto the cortical surface regions, which were divided into 12 networks (Gordon et al., 2014), including the auditory (AN), visual (VN), and sensorimotor networks for hand (SHN) and mouth (SMN), cingulo-opercular (CON), cingulo-parietal (CPN), dorsal attention (DAN), default mode (DMN), fronto-parietal (FPN), retrosplenial temporal (RTN), salience (SN), and ventral attention (VAN) networks. Resting-state functional connectivity strength indices were calculated using the averaged Fisher-transformed Pearson correlation coefficient between pairs of regions within and between these 12 networks (*n* = 78), and between the 10 subcortical regions (cerebellum cortex; thalamus; hippocampus; amygdala; putamen; pallidum; caudate; nucleus accumbens; ventral diencephalon; brain-stem) and 12 cortical networks (*n* = 120).

### 2.5 Association analyses

The associations between peer environments (PFI, DFI), behavioral (including psychiatric variables), and neuroimaging measures were estimated using linear mixed-effects models (LMMs), implemented via the R package lmerTest (Kuznetsova et al., 2017). Peer environments were modeled as predictors, and behavioral/imaging measurements were the dependent variables in LMMs. Covariates included sex, age, race, family income, and parental education, with random effects for family nested within sites (MRI scanners for imaging-related analyses). To account for individual variations in head size and motion during scanning, intracranial volume (ICV) and head motion parameters served as covariates for brain structural and functional analyses, respectively. LMM provided a *t*-value for each regression coefficient (*β*), with higher *t*-values indicating stronger associations between peer environment and the dependent variables. The semi-partial R^2^ (R^2^_s*p*_) serves as the effect size (Nakagawa and Schielzeth, 2013), calculated by using the R package r2glmm (Jaeger, 2017).

False discovery rate (FDR) (Benjamini and Hochberg, 1995) corrections were used for multiple comparisons with significance set at 0.05 in each set of variables. FDR corrections were applied 57 times for behavioral-cognitive variables (57 scores), 68 times for cortical metrics (68 regions), 16 times for subcortical volumes (16 regions), 78 times for network connectivity (78 RSFCs), and 120 times for cortico-subcortical connectivity (120 RSFCs).

### 2.6 Relationships between the brain patterns of peer environments and neurotransmitter densities

We additionally examined topographical relationships between the whole-brain association patterns of peer environments and neurotransmitter density distributions (Fig. 1c). Specifically, the whole-brain association patterns of peer environments were represented by *t*-maps derived from the associations between peer environments and brain structures. For instance, 68 *t*-values from the associations between the PFI and 68 regional volumes were used to represent the whole-brain association patterns of PFI and cortical volumes. Together, 6 (3 cortical metrics × 2 peer environments) *t*-maps were used in our analysis. The 12 receptor or transporter density maps were generated using average group maps produced from other different studies, including 3 serotonin receptors (5-HT_1A_, 5-HT_1B_, 5-HT_2A_) (Savli et al., 2012), 1 transporter (5-HTT), 2 dopamine receptors (D_1_, D_2_), dopamine transporter (DAT) (Dukart et al., 2018, Alakurtti et al., 2015, Kaller et al., 2017), and F-DOPA (reflecting presynaptic dopamine synthesis capacity) (García-Gómez et al., 2013), 1 acetylcholine transporter (VAChT) (Hansen, 2022), 1 gamma-aminobutyric acid receptor (GABA_a_) (Dukart et al., 2018), 1 noradrenaline transporter (NAT) (Hesse et al., 2017), and 1 glutamate receptor (mGluR5) (Hansen, 2022). Spearman correlations between the *t*-maps and neurotransmitter density maps were calculated using the JuSpace toolbox (Dukart et al., 2021). Significance was tested via 1000 spatial-spin permutations and FDR corrections were used for multiple comparison corrections (*n* = 12 neurotransmitters).

### 2.7 Mediation analyses

To investigate how peer environments impact behaviors through brain morphometrics or pathways, mediation analyses were performed using the Mediation Toolbox (Kenny et al., 2003). Specifically, brain measures (both sMRI and RSFCs, showing significant results in association analyses) served as mediators, peer environments were the independent variables, and behavioral variables were designated as dependent variables (Fig. 4a-b). The covariates are the same as LMMs (age, sex, race, family, family income, parental education, MRI scanners, ICV for brain structures, and head motion for RSFCs). The significance was evaluated using the bootstrap approach (10,000 random samplings) with FDR corrections used for *p*-value adjustment (*n* = the number of significant brain regions/RSFCs × 57 behavioral variables).

**Fig. 2:**
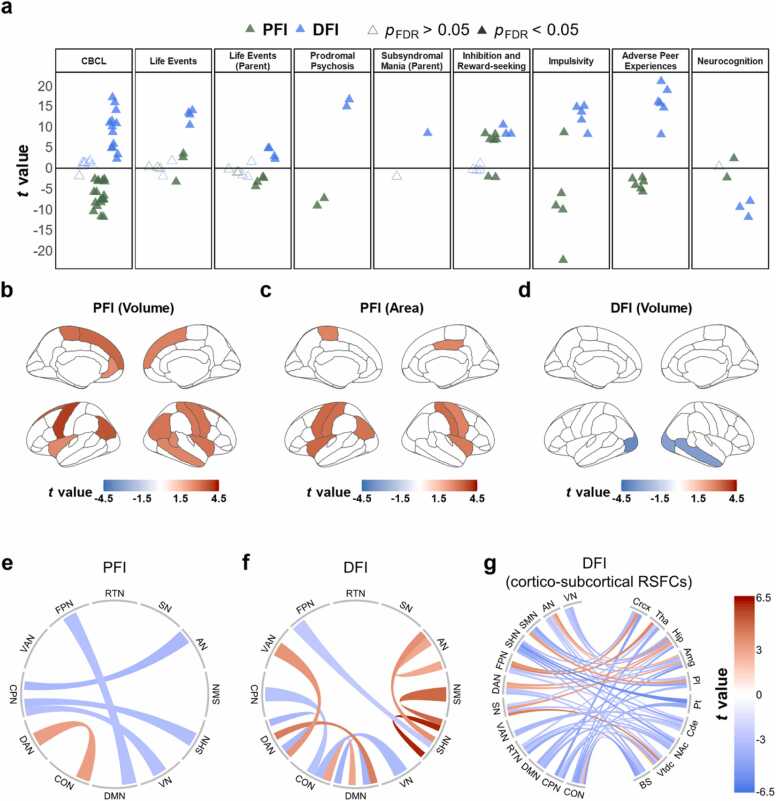
Associations between peer environments and psychopathology-relevant behavior, regional brain volume, and RSFCs. (a) Association between peer environments (PFI, DFI) and behavioral measurements (*p*_fdr_ < 0.05, 57 multiple comparisons). Each triangle represented one behavioral item. There are 57 behavioral items, including CBCL (20 items), self-report life events (6 items), parent-report life events (7 items), prodromal psychosis (2 items), parent-report subsyndromal mania (1 item), inhibition and reward-seeking (7 items), impulsivity (5 items), adverse peer experiences (6 items), neurocognition (3 items). (b-c) Associations between PFI and 14 cortical volumes and 10 cortical areas (FDR correction times = 68, *p*_fdr_ < 0.05). (d) Associations between DFI and 3 cortical volumes (*p*_fdr_ < 0.05). (e-f) Associations between peer environments and RSFCs among 12 cortical networks. 5 significant RSFCs for PFI, 12 significant RSFCs and DFI, respectively (FDR correction times = 78 within and between network RSRCs, *p*_fdr_ < 0.05). (g). Associations between DFI and 46 cortico-subcortical RSFCs (FDR correction times = 120 cortico-subcortical RSFCs, *p*_fdr_ < 0.05). Abbreviation: AN = auditory network; VN = visual network; SHN = sensorimotor hand network; SMN = sensorimotor mouth network; CON = cingulo-opercular network; CPN = cingulo-parietal network; DAN = dorsal attention network; DMN = default mode network; FPN = fronto-parietal network; RTN = retrosplenial temporal network; SN = salience network; VAN = ventral attention network; Crcx = cerebellum cortex; Tha = thalamus; Hip = hippocampus; Amg = amygdala; Pt = putamen; Pl = pallidum; Cde = caudate; NAc = nucleus accumbens; Vtdc = ventral diencephalon; BS = brain-stem.

**Fig. 3:**
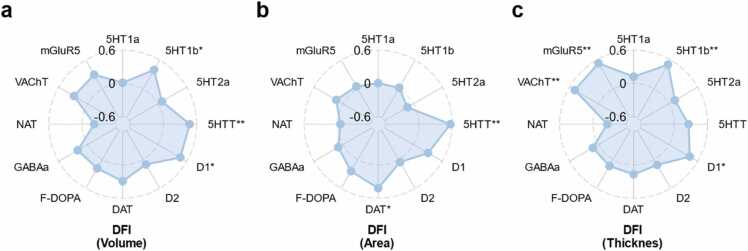
Spatial correlations between *t*-maps of brain structure-DFI associations and neurotransmitter density. Neurotransmitters included 4 dopamine receptors or transporters (D1, D2, DAT, F-DOPA), 4 serotonin receptors or transporters (5-HTT, 5-HT_1a_, 5-HT1_2a_, 5-HT_2b_), acetylcholine transporter (VAChT), gamma-aminobutyric acid receptor (GABA_a_), noradrenaline transporter (NAT), and metabotropic glutamate receptor (mGluR5). The associations between neurotransmitter densities and the *t*-maps (brain correlation patterns). a-c depicts associations between the *t*-maps (volume, area, thickness) from the DFI and neurotransmitter density maps. FDR corrections were used for multiple comparisons (12 neurotransmitters). * and * * indicate *p*_fdr_ < 0.05, *p*_fdr_ < 0.01, respectively.

**Fig. 4:**
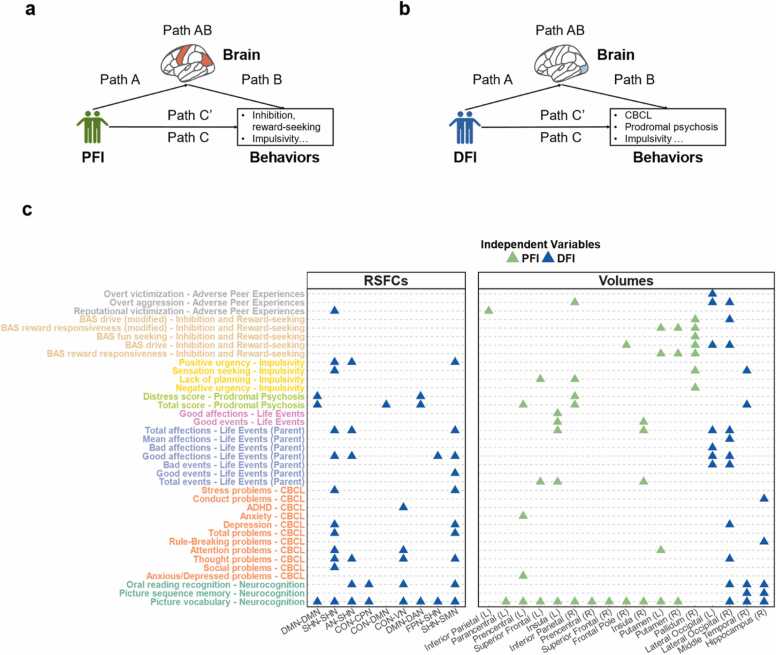
The brain features mediated the relationships between peer environments and behaviors. Each triangle/circle represents one peer-behavior relationship. (a) The mediation model of PFI. M (mediator) = brain features (significantly associated with PFI), X (independent variable) = PFI, and Y (dependent variable) = 57 behavioral-cognitive measurements measurements (e.g., for brain volumes, FDR correction times = 18 significant cortical and subcortical brain volumes × 57 behavioral-cognitive measurements). (b) The mediation model of DFI. M = brain features (significantly associated with DFI), X = DFI, and Y = 57 behavioral-cognitive measurements (e.g., for RSFCs, FDR correction times = 12 significant RSFCs × 57 behavioral-cognitive measurements). (c) The significant relationships between peer environments and behaviors were significantly mediated by brain features (regional volumes and RSFCs, *p*_fdr_ < 0.05). Abbreviation: AN = auditory network; VN = visual network; SHN = sensorimotor hand network; SMN = sensorimotor mouth network; CON = cingulo-opercular network; CPN = cingulo-parietal network; DAN = dorsal attention network; DMN = default mode network.

### 2.8 Longitudinal analyses

**Fig. 5:**
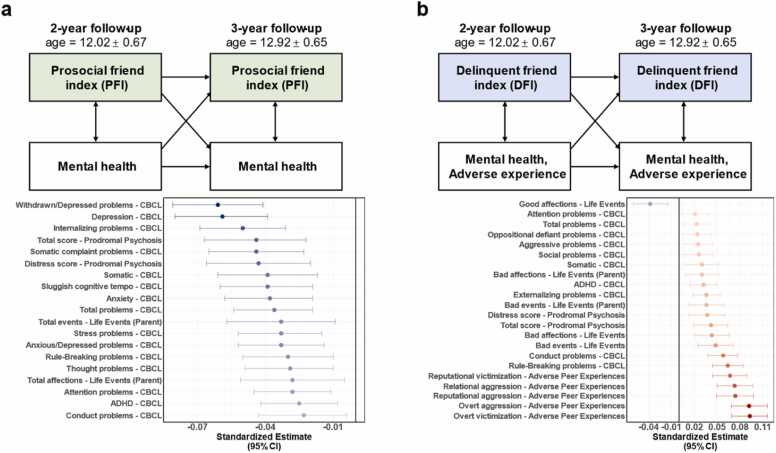
Longitudinal analysis. The longitudinal relationship of 5916 adolescents between peer environments at 2-year follow-up and behaviors at 3-year follow-up (2-year follow-up: age = 12.02 ± 0.67; 3-year follow-up: age = 12.92 ± 0.65). (a) PFI at 2YFU had negative associations with 19 variables next year, including 15 behavioral problem scores (e.g., withdrawal/depression, internal, depressive, and sluggish cognitive tempo), 2 parent-report life events, and 2 prodromal psychosis scores. (b) DFI at 2YFU had positive associations with 22 variables at 3YFU, including 10 behavioral problem scores (e.g., external problem, ADHD, conduct problem, total problem), 2 prodromal psychosis scores, 3 life events, 2 parent-report life events and 5 adverse peer experiences (reputation aggression, reputation victimization, overt victimization, overt aggression, and relational aggression). FDR corrections were used for multiple comparisons (*p*_fdr_ < 0.05, *n* = 41 behavioral variables).

To investigate whether peer environments had a long-term relationship with behavioral problems that potentially be advantageous for clinical interventions, two-wave cross-lag panel models (CLPM) were performed using the R package lavvan (Curtis et al., 2020). CLPM was estimated by using the maximum likelihood algorithm, and the cross-lagged effects were considered bidirectional. A simple illustration was presented in Fig. 5. A total of 41 behavioral, and adverse experiences variables were available at both 2YFU and 3YFU were included (Supplementary Table 2). The 2YFU assessments were designated as the first time point, and the 3YFU assessments were set as the second time point (because 3YFU assessments had more available variables than other follow-up assessments). Confounding variables were regressed before running CLPM, consistent with the behavioral LMMs (age, sex, race, family income, parental education, family nested within sites). FDR corrections were used for multiple comparison corrections (*n* = 41 behavioral variables).

### 2.9 Supplementary analyses

During adolescence, peer influence intertwines with various other factors, particularly socio-environmental factors such as family (Nash et al., 2005), neighborhood and school (Oberle et al., 2011). To further mitigate the influence of these factors, family conflict, school risk and protective factors, neighborhood safety, school grades from last year were included in supplementary analyses. Given that PFI and DFI indicate the proportion of different types of friends, the number of male and female friends were also included as confounding factors (Supplementary Table 7).

Additionally, a 5-fold cross-validation procedure was conducted by fitting LMMs to assess the robustness of effect size from the primary LMMs (Goltermann et al., 2023) (Supplementary Methods). Besides, 80 % of the samples were randomly selected, and the same LMM procedures were repeated 500 times (sampling LMMs) to evaluate the robustness of the primary LMMs. Other sets of 8 available neurotransmitter density maps were used to validate topographical relationships between the brain patterns of peer environments and neurotransmitter densities. For longitudinal analyses, the bivariate latent change score model (Kievit et al., 2018) was utilized to validate the results obtained from CLPM.

## 3 Results

### 3.1 Peer environments are associated with psychopathology-relevant behavior in early adolescence

In general, PFI was negatively associated with most psychopathology-relevant behavioral problems, while DFI was positively associated with these variables (Fig. 2a, *p*_fdr_ < 0.05). Specifically, a larger PFI was associated with fewer behavioral problems (19 out of 20 items), parent-reported life events (4 out of 7 items), prodromal psychosis, impulsivity (4 out of 5 items), and adverse peer experiences. For DFI, we found positive associations with more behavioral problems (13 out of 20 items), parent-report subsyndromal mania, self-report life events (4 out of 6 items), parent-report life events (4 out of 7 items), prodromal psychosis, impulsivity, adverse peer experiences, and neurocognition. Additionally, both PFI (5 out of 7 items) and DFI (3 out of 7 items) had positive associations with inhibition and reward-seeking. Detailed results were provided in Supplementary Table 3–4.

### 3.2 Peer environments are associated with the structural and functional architecture of the adolescent brain

We found that brain structures were generally positively associated with PFI, but negatively associated with DFI. Specifically, the total brain volume correlated positively with PFI (*t* = 4.44, R^2^_s*p*_ = 0.0019, *p* = 2.8 × 10^−5^), while negatively with DFI (*t* = -3.04, R^2^_s*p*_ = 0.0009, *p* = 0.002). For cortical volumes, PFI was positively associated with 14 regions, including the right insula (*t* = 3.04, R^2^_s*p*_ = 0.0014, *p*_fdr_ = 0.021) and lateral frontal and parietal areas (*p*_fdr_ < 0.05, Fig. 2b). In contrast, DFI exhibited negative correlations with 3 cortical volumes, including the right middle temporal and bilateral lateral occipital regions (Fig. 2d). For subcortical volumes, PFI also exhibited positive correlations with 4 subcortical volumes, encompassing the right accumbens, right pallidum, and the bilateral putamen (Supplementary Table 3). Conversely, DFI showed a negative association with the hippocampal volume (*t* = -3.09, R^2^_s*p*_ = 0.0015, *p*_fdr_ = 0.032). Similarly, total brain cortical surface area was positively correlated with PFI (*t* = 3.75, R^2^_s*p*_ = 0.0012, *p* = 1.8 × 10^−4^), with PFI showing positive associations with 10 areas, including the left inferior parietal region (Fig. 2c). For cortical thickness, DFI displayed negative relationships with 3 regions, including the right middle temporal and bilateral lateral occipital areas (Supplementary Fig. 2).

For RSFCs, different peer environments demonstrate partly opposite patterns of association, especially in the DMN and sensory networks. For example, PFI had a negative association with RSFCs between DMN and FPN (*t* = -3.22, R^2^_s*p*_ = 0.0017, *p*_fdr_ = 0.028, Fig. 2e). By contrast, DFI had positive correlations between DMN with attention networks (CON; DAN) as well as within DMN (*t* = -3.88, R^2^_s*p*_ = 0.0023, *p*_fdr_ = 0.002, Fig. 2 f). Besides, PFI was also associated with lower RSFCs between the CPN and three sensory networks (AN, VN, SHN), while DFI had positive associations with RSFCs among different sensory networks (AN, SHN, SMN). For cortical-subcortical RSFCs, PFI exhibited positive associations with 2 cortico-subcortical RSFCs (DMN-Vtdc, SMN-Cde), Whereas DFI exhibited associations (most were negative) with 46 cortico-subcortical RSFCs, especially in cortico-limbic and frontostriatal circuits (*p*_fdr_ < 0.05, Fig. 3 g). For instance, DFI was negatively associated with the CON-amygdala connectivity (*t* = -4.93, R^2^_s*p*_ = 0.004, *p*_fdr_ = 4.9 × 10^−5^). Detailed results were listed in Supplementary Table 3–4.

### 3.3 Topographical associations between peer-environment-related structural variations and receptor/transporter densities

Overall, DFI-structural MRI associations exhibited widespread spatial correlations with various neurotransmitter systems. Specifically, the *t*-map (volume) from DFI had positive associations with 3 neurotransmitter markers: 5HT1b (*ρ* = 0.40, *p*_fdr_ = 0.036), 5-HTT (*ρ* = 0.48, *p*_fdr_ = 0.008), and D1 (*ρ* = 0.47, *p*_fdr_ = 0.020, Fig. 3a). Similarly, the *t*-map for cortical surface area demonstrated positive associations with one serotonin transporter (5-HTT, *ρ* = 0.57, *p*_fdr_ = 0.002) and one dopamine transporter (DAT, *ρ* = 0.42, *p*_fdr_ = 0.01, Fig. 3c). Moreover, The *t*-map for cortical thickness further revealed positive correlations with 4 neurotransmitter systems: the acetylcholine transporter (VAChT, *ρ* = 0.49, *p*_fdr_ = 0.007), the metabotropic glutamate receptor (mGluR5, *ρ* = 0.53, *p*_fdr_ = 0.007), one serotonin receptor (5-HT1b, *ρ* = 0.51, *p*_fdr_ = 0.007), one dopamine receptor (D1, *ρ* = 0.44, *p*_fdr_ = 0.037, Fig. 3b). Besides, no significant associations were found between the *t*-maps from PFI and neurotransmitters (Supplementary Fig. 3b).

### 3.4 Brain architecture mediates the relationship between peer environments and behavioral profiles

We next investigated whether brain measures mediate the relationships between peer environments and behavioral phenotypes (Fig. 4a-b). In general, regional volumes mediated the associations between PFI and neurocognition, as well as psychopathology-relevant behaviors, whereas RSFCs mediated the relationships between DFI and those measurements (Fig. 4c). Specifically, 41 mediation effects for brain volumes (multiple comparison times = 18 brain volumes × 57 behavioral-cognitive variables) were found in the relationships between PFI and behavioral-cognitive variables, especially in inhibition and reward-seeking, and impulsivity (*p*_fdr_ < 0.05). For example, the right inferior parietal volume mediated the relationship between PFI and prodromal psychosis distress scores (path AB, *β* = −0.0055, 95 % CI = [-0.0074, −0.0042], mediation proportion = 2.37 %, *p*_fdr_ = 0.046). Moreover, 28 mediation effects for brain volumes (multiple comparison times = 4 brain volumes × 57 behavioral-cognitive variables) were found in the relationships between DFI and behaviors, especially in parent-reported life events and neurocognition (*p*_fdr_ < 0.05, Fig. 4c).

Alternatively, we identified 44 associations between DFI and behavioral-cognitive variables, particularly those related to psychopathology, that were mediated by RSFCs (Fig. 4c). For instance, the within DMN connectivity mediated the relationship between DFI and prodromal psychosis (total score, path AB, *β* = 0.0075, 95 % CI = [0.0060, 0.0095], mediation proportion = 1.96 %, *p*_fdr_ = 0.011). For cortico-subcortical RSFCs, 199 significant mediation effects were identified between DFI and behavioral problems, with the majority of significant mediators involving frontal-limbic and frontostriatal RSFCs (*p*_fdr_ < 0.05, Supplementary Fig. 4). Detailed results were listed in Supplementary Table 5.

### 3.5 Longitudinal analysis of peer environments and behavioral variables

Overall, PFI at 2YFU displayed negative associations with internal mental problems at 3YFU, whereas DFI at 2YFU exhibited positive correlations with external mental problems and adverse peer experiences at 3YFU (*p*_fdr_ < 0.05; corrected for 41 comparisons). Specifically, PFI at 2YFU was negatively associated with 19 variables at 3YFU (15 behavioral problems, 2 parent-reported life events, and 2 prodromal psychosis scores, Fig. 5a). For example, PFI at 2YFU was negatively associated with the depression problem score at 3YFU (standardized *β* = −0.059, 95 % CI = [-0.080, –0.039], *p*_fdr_ = 2.9 × 10^−7^). In comparison, DFI at 2YFU correlated with 22 variables at 3YFU (10 behavioral problem scores, 2 prodromal psychosis scores, 2 parent-reported life events, 3 life events, and 5 adverse peer experiences, Fig. 5b). For instance, DFI at 2YFU had positive associations with the ADHD problem score (standardized *β* = 0.032, 95 % CI = [0.015, 0.049], *p*_fdr_ = 9.1 × 10^−4^) and overt victimization (standardized *β* = 0.093, 95 % CI = [0.069, 0.116], *p*_fdr_ = 4.6 × 10^−13^) at 3YFU. All detailed results have been listed in Supplementary Table 6.

### 3.6 Supplementary analyses: control of potential confounders and validation

The primary results remained stable while we additionally controlled for the school grade from last year, family conflict, neighborhood safety, school risk and protective factors, and the number of male and female friends, underscoring the unique contribution of peer environment (Supplementary Fig. 5–11).

Effect size estimation based on 5-fold cross-validation of LMMs (Supplementary Fig. 12–13), and 500 times sampling LMMs (Supplementary Table 3–4), also indicated that the primary results of LMMs were stable. Besides, another set of eight neurotransmitter density maps also found stable results regarding the associations between neurotransmitter density maps and the *t*-maps from DFI (Supplementary Fig. 14). Lastly, the bivariate latent change score model (Kievit et al., 2018) was used to validate the results of longitudinal analyses, and similar results were still observed (Supplementary Fig. 15).

## 4 Discussion

Capitalizing on a large-scale database, we demonstrate a robust association between both prosocial and delinquent peer environments during adolescence with mental health, cognitive performance, and the structural and functional organization of the brain as well as developmental trajectories. Overall, a higher proportion of prosocial friends correlated with positive behavioral outcomes and associated neural patterns, while more delinquent friends corresponded with adverse outcomes and related neural alterations.

At the behavioral level, we found that a larger proportion of prosocial friends was associated with a lower level of problem behaviors, adverse experiences, prodromal psychosis, and impulsivity, while in contrast a higher proportion of delinquent friends was associated with higher levels of pathology-relevant behaviors. These findings align with a growing body of literature suggesting a complex and reciprocal interaction between social-emotional peer experiences (early threat experiences (Sumner et al., 2019) or peer victimization (Reijntjes et al., 2010)) and mental health (Fusar-Poli et al., 2012). Collectively, these findings highlight the potential influence of prosocial friendships in fostering positive and resilient developmental pathways, while more delinquent friends suggest an elevated risk for emotional problems and potentially adverse developmental trajectories. Interestingly, both PFI and DFI were positively associated with behavioral activation/reward-seeking, consistent with prior studies showing a substantial peer influence on reward and inhibitory processes (Jongen et al., 2011) driven by increased risky behaviors in the presence of peers (Chein et al., 2011, Smith et al., 2014). These findings might suggest that increased risky behavior – due to social conformity from peers instead of their prosocial or delinquent nature.

At the brain structure level, the associations between behavioral and psychopathological indices were mirrored in opposing effects on the structural and functional organization of the brain. PFI was positively associated with volumes of the insula, regions consistently implicated in social cognitive and emotional development during adolescence (Blakemore, 2008). Additionally, PFI was linked to a larger volume in the left inferior parietal, which is also social cognition and language (Bzdok et al., 2016). Furthermore, PFI demonstrated associations with larger volumes in striatal subregions, including the pallidum, which plays a key role in emotion and motor processing (Strakowski et al., 2005), and the putamen, which is involved in learning and regulatory control (Zhao et al., 2021). Reduced volumes in these regions have been linked to psychopathology; for instance, a lower pallidum volume has been observed in pediatric bipolar disorder (Zhang et al., 2022), and reduced putamen volume has been prospectively associated with emotional and behavioral dysregulation in prior studies (Becker et al., 2015, Zhou et al., 2020). These results therefore may reflect that a prosocial peer environment might be associated with better emotion and regulatory control to potentially cope with mental problems. In contrast to PFI, DFI exhibited reductions in lateral occipital volume and thickness, regions linked to multisensory processing (Beauchamp, 2005). This aligns with our observations of stronger associations between DFI and sensorimotor networks.

At the functional network level, a higher proportion of prosocial friends was negatively associated with connectivity between DMN and FPN, while hyperconnectivity between DMN and FPN has been associated with depression (Brandl et al., 2022) (negative associations between PFI and depression score were observed in association analyses). These results suggested that adolescents with more prosocial friends might experience a reduced vulnerability to mental disorders. In contrast, delinquent friends were also associated with widespread neurofunctional alterations, in particular with the intrinsic organization of DMN, which is related to increased adverse life events (Karcher et al., 2019). These findings align with recent ABCD studies suggesting an association between DMN integrity and higher general psychopathology (Karcher et al., 2021), which indicated that delinquent friends were linked with dysfunction in social emotion regulation which may promote mental illness. Additionally, DFI was negatively linked to cortico-limbic and cortico-striatal functional connectivity. Specifically, the CON-amygdala RSFC was associated with stressful family environments (Thijssen et al., 2020), which is also consistent with the stress acceleration hypothesis suggesting that adolescents with early stress experiences could accelerate maturation to cope with adversity (Callaghan and Tottenham, 2016). Thus, these results might reflect that adolescents with more delinquent peers may experience greater exposure to stress.

The DFI-brain signatures are associated separably with neurotransmitter density distribution maps (as derived from other independent studies) on the molecular architecture level. Firstly, the relationship between DFI and brain morphology (volume, area) was more prominent in regions with greater 5-HTT density, which has been linked to the effects of sustained early-life stress in rhesus macaques (Kinnally et al., 2010) and humans (Liu et al., 2021). Moreover, DFI-thickness correlations spatially overlapped with mGluR5 receptor distributions, which are known to regulate discrete social behaviors (Mesic et al., 2015), mirroring our behavioral findings linking DFI to life events and problematic social behaviors (Fig. 2a). Similarly, DFI-structure associations (both volume and thickness) aligned with D1-type dopaminergic system distributions, which have been strongly related to reward (Glimcher, 2011). This may mirror our findings that DFI was positively correlated with reward-seeking and impulsivity (Fig. 2a). Together, these findings reinforce our association analyses and highlight the potential molecular underpinnings of these relationships.

Further mediation analyses highlighted that PFI (brain volumes) and DFI (RSFCs) may shape behaviors through distinct neural pathways. Specifically, the association between PFI and prodromal psychosis by the inferior parietal, a brain region linked to psychosis transition (Fusar-Poli et al., 2011). This finding supports our hypothesis that prosocial friends may serve as a protective factor, potentially mitigating behavioral and mental health problems through neurodevelopmental pathways. The relationship between DFI and prodromal psychosis and critically mediated by different network RSFCs such as the DMN-DMN pathway, which has been positively correlated with poor outcomes in adolescent psychosis (Collin et al., 2020). These findings underscore the role of DMN in the influence of delinquent peer associations on psychotic symptoms. Additionally, the cortico-limbic RSFCs mediated the relationships between DFI and behavioral problems. Given the negative association between CON-amygdala RSFC and adverse life events (Brieant et al., 2021), associations with delinquent friends might signify a stressful environment, linked with mental health and connectivity patterns.

Furthermore, the longitudinal analyses found that more prosocial friends had negative associations with internalizing problems (e.g., depression) one year later whereas more delinquent friends had positive associations with external problems (e.g., ADHD), suggesting the significance of a positive peer environment for preventing behavioral problems in adolescents (Walters, 2020, Van Ryzin and Leve, 2012). In line with our findings, prosocial peer treatment was shown to help alleviate depressive affect in children (Troop-Gordon and Unhjem, 2018), and peer delinquency was related to later externalizing behaviors of adolescents (Brook et al., 2011). These results suggested that different strategies should be used in behavioral interventions in adolescents. Additionally, more delinquent friends had associations with more peer victimization one year later, which also emphasizes the importance of interventions toward this peer group for preventive purposes.

This study has several limitations. First, the self-report six-item measurements may not fully capture the complexity of peer environments. Future studies should include assessments from parents, teachers, and more diverse behaviors. Second, the relatively small effect sizes for PFI/DFI in LMMs suggest that adolescent development is shaped by multiple interacting factors. Nevertheless, small effects may accumulate over time and have significant population-level impacts (Funder and Ozer, 2019). Third, neurotransmitter density maps from external datasets were utilized instead of data directly from adolescents themselves, resulting in a relatively coarse analysis. Fourth, cross-sectional analyses (e.g., association and mediation analyses) were utilized in the current study while the adolescent brain undergoes substantial developmental alterations, such as cortical thickness reduction^3^ and function network changes (Casey et al., 2019). Further longitudinal research is warranted to explore how peers impact the longitudinal development of the adolescent brain.

In conclusion, we demonstrate the significant influence of peer environments on both vulnerability and resilience to mental health challenges during early adolescence. More importantly, these findings carry implications for clinical behavioral interventions targeting adolescents with mental health concerns.

## CRediT authorship contribution statement

**Sahakian Barbara J.:** Writing – review & editing. **Zhao Xingming:** Project administration. **Lian Zhengxu:** Project administration. **Robbins Trevor W.:** Writing – review & editing. **Fan Huaxin:** Project administration. **Becker Benjamin:** Writing – review & editing. **Kuang Nanyu:** Project administration. **Zhang Jie:** Writing – review & editing, Supervision. **Gu Xinrui:** Project administration. **Yang Senyou:** Project administration. **Hu Yechen:** Project administration. **Jiang Xi:** Project administration. **Zhang Yufeng:** Project administration. **Liu Yu:** Writing – original draft, Visualization, Validation, Methodology, Investigation. **Cheng Wei:** Project administration. **Peng Songjun:** Writing – review & editing. **Feng Jianfeng:** Resources, Project administration. **Wu Xinran:** Writing – review & editing, Visualization. **Liu Zhaowen:** Writing – review & editing.

## Ethics declarations

Participants from the ABCD project of the National Institute of Mental Health National Data Archive release 5.1. The University of California, San Diego granted centralized IRB approval, with each participating site obtaining its own local IRB approval.

## Declaration of Competing Interest

The authors declare that they have no known competing financial interests or personal relationships that could have appeared to influence the work reported in this paper.

## Data Availability

The authors do not have permission to share data.
