# Supplementary material for: Neural, cognitive and psychopathological signatures of a prosocial or delinquent peer environment during early adolescence
Source: Dev Cogn Neurosci. 2025 May 8;73:101566. doi: 10.1016/j.dcn.2025.101566 (PMC12140950; doi:10.1016/j.dcn.2025.101566)
Supplement: Supplementary file 1 — Supplementary material [file mmc1.docx]

**Supplementary Methods**

**Cross-validation of effect sizes.** 5-fold cross-validation was implemented by fitting LMMs to the training sets (comprising 4-fold data) to estimate effect sizes and subsequently applying the resulting linear coefficients to the corresponding test sets (the remaining one-fold data)^1^. To get the predictive value of response variables (behavioral and brain variables) in the test set, the resulting beta coefficients yielded by model estimation within the train set were applied to the test set. Residuals were computed as the differences between the actual response variables (behavioral and brain variables) and their predicted values in the test set. In the test set, the sum of squared residuals (SSR) was computed for both the full model (including PFI/DFI as a fixed effect) and the reduced model (excluding PFI/DFI). The R2 *p* for PFI/DFI was then obtained using the following formula. Finally, the mean R2 *p* for PFI/DFI was calculated across the five test sets as CV-based effect sizes. Note that models cannot run in the test set due to the nest structure of sites/scanners and family IDs (5,228-6,674 unique family IDs) even if in 2-fold cross-validation. To avoid this situation, the family IDs were removed from the random effect set as a fixed effect cross-validation.

R2 *p*= SSR_reduced_ – SSR_full_ / SSR_reduced_

**Supplementary Tables**

**Supplementary Table 1** **|** Demographic information of participants in each analysis. Abbreviation: sMRI = structural MRI; RSFC = resting-state functional connectivity; 2YFU = 2-year follow-up; 3YFU = 3-year follow-up.

| **Analysis** | ***n***  **(female %)** | **Age**  **(SD)** | **Income***  **(SD)** | **Education***  **(SD)** | **Race*** |
| --- | --- | --- | --- | --- | --- |
| Association (Behavior)  Association (sMRI)  Association (RSFCs)  Mediation (sMRI)  Mediation (RSFCs) | 7,806  (48.14%) | 12.02  (0.67) | 7.64  (2.22) | 18.19  (3.05) | 4,514/994/1,401/134/813 |
|  | 6,242  (46.84%) | 11.95  (0.65) | 7.58  (2.25) | 18.06  (3.15) | 3,593/735/1,166/112/636 |
|  | 6,003  (47.26%) | 11.97  (0.65) | 7.64  (2.20) | 18.13  (3.12) | 3,511/668/1,111/105/606 |
|  | 5,823  (47.38%) | 11.96  (0.65) | 7.65  (2.17) | 18.17  (3.05) | 3,441/676/1,203  /90/593 |
|  | 5,165  (47.78%) | 11.97  (0.65) | 7.69  (2.14) | 18.21  (3.02) | 3,087/569/906  /79/524 |
| Longitudinal (2YFU) | 5,916  (49.15%) | 12.02  (0.67) | 7.82  (2.04) | 18.33  (2.84) | 3,607/610/991  /86/622 |
| Longitudinal (3YFU) | 5,916  (49.15%) | 12.92  (0.65) | 7.88  (20.03) | 18.31  (2.81) | 3,607/610/991  /86/622 |

Income: The total family income.

Education: The educational status of the more educated parent.

Race: White/Black/Hispanic/Asian/Other.

**Supplementary Table 2 |** A total of 57 behavioral variables were used in our analyses. The association and mediation analyses used 57 behavioral variables that were available at 2YFU. The longitudinal analysis used 41 variables that both were available at 2YFU and 3YFU. Abbreviations: 2YFU = 2-year follow-up. 3YFU = 3-year follow-up. * indicates parent-reported inventory.

| **Behavioral Variables (n = 57)** | **Category** | **Inventory** | **2YFU** | **3YFU** |
| --- | --- | --- | --- | --- |
| Picture Vocabulary | Neruocgontion | nc_y_nihtb | √ |  |
| Picture Sequence Memory | Neruocgontion | nc_y_nihtb | √ |  |
| Oral reading recognition | Neruocgontion | nc_y_nihtb | √ |  |
| Anxious/Depressed problems | CBCL | mh_p_cbcl | √ | √ |
| Withdrawn/Depressed problems | CBCL | mh_p_cbcl | √ | √ |
| Somatic complaint problems | CBCL | mh_p_cbcl | √ | √ |
| Social problems | CBCL | mh_p_cbcl | √ | √ |
| Thought problems | CBCL | mh_p_cbcl | √ | √ |
| Attention problems | CBCL | mh_p_cbcl | √ | √ |
| Rule-Breaking problems | CBCL | mh_p_cbcl | √ | √ |
| Aggressive problems | CBCL | mh_p_cbcl | √ | √ |
| Internalizing problems | CBCL | mh_p_cbcl | √ | √ |
| Externalizing problems | CBCL | mh_p_cbcl | √ | √ |
| Total problems | CBCL | mh_p_cbcl | √ | √ |
| Depression | CBCL | mh_p_cbcl | √ | √ |
| Anxiety | CBCL | mh_p_cbcl | √ | √ |
| Somatic | CBCL | mh_p_cbcl | √ | √ |
| ADHD | CBCL | mh_p_cbcl | √ | √ |
| Oppositional defiant problems | CBCL | mh_p_cbcl | √ | √ |
| Conduct problems | CBCL | mh_p_cbcl | √ | √ |
| Sluggish cognitive tempo | CBCL | mh_p_cbcl | √ | √ |
| OCD | CBCL | mh_p_cbcl | √ | √ |
| Stress problems | CBCL | mh_p_cbcl | √ | √ |
| General behavior total score* | Subsyndromal Mania (Parent) | mh_p_gbi | √ |  |
| Life events (total)* | Life Events (Parent) | mh_p_le | √ | √ |
| Life events (good)* | Life Events (Parent) | mh_p_le | √ | √ |
| Life events (bad)* | Life Events (Parent) | mh_p_le | √ | √ |
| Life events (good affections)* | Life Events (Parent) | mh_p_le | √ | √ |
| Life events (bad affections)* | Life Events (Parent) | mh_p_le | √ | √ |
| Life events (mean affections)* | Life Events (Parent) | mh_p_le | √ | √ |
| Life events (total affections)* | Life Events (Parent) | mh_p_le | √ | √ |
| Life events (total) | Life Events | mh_y_le | √ | √ |
| Life events (good) | Life Events | mh_y_le | √ | √ |
| Life events (bad) | Life Events | mh_y_le | √ | √ |
| Life events (sum affections) | Life Events | mh_y_le | √ | √ |
| Life events (good affections) | Life Events | mh_y_le | √ | √ |
| Life events (bad affections) | Life Events | mh_y_le | √ | √ |
| Prodromal psychosis (total) | Prodromal Psychosis | mh_y_pps | √ | √ |
| Prodromal psychosis (distress) | Prodromal Psychosis | mh_y_pps | √ | √ |
| Negative urgency | Impulsivity | mh_y_upps | √ |  |
| Lack of planning | Impulsivity | mh_y_upps | √ |  |
| Sensation seeking | Impulsivity | mh_y_upps | √ |  |
| Positive urgency | Impulsivity | mh_y_upps | √ |  |
| Lack of perseverance | Impulsivity | mh_y_upps | √ |  |
| BIS sum score | Inhibition and Reward-seeking | mh_y_bisbas | √ |  |
| BAS reward responsiveness | Inhibition and Reward-seeking | mh_y_bisbas | √ |  |
| BAS drive | Inhibition and Reward-seeking | mh_y_bisbas | √ |  |
| BAS fun seeking | Inhibition and Reward-seeking | mh_y_bisbas | √ |  |
| BIS sum score (modified) | Inhibition and Reward-seeking | mh_y_bisbas | √ |  |
| BAS reward responsiveness (modified) | Inhibition and Reward-seeking | mh_y_bisbas | √ |  |
| BAS drive (modified) | Inhibition and Reward-seeking | mh_y_bisbas | √ |  |
| Relational Victimization | Adverse Peer Experience | mh_y_peq | √ | √ |
| Reputational Aggression | Adverse Peer Experience | mh_y_peq | √ | √ |
| Reputational Victimization | Adverse Peer Experience | mh_y_peq | √ | √ |
| Overt Aggression | Adverse Peer Experience | mh_y_peq | √ | √ |
| Overt Victimization | Adverse Peer Experience | mh_y_peq | √ | √ |
| Relational Aggression | Adverse Peer Experience | mh_y_peq | √ | √ |

**Supplementary Table 3 |** Significant results of association analyses between prosocial friends index (PFI) and 48 behavioral-cognitive variables and brain structures (14 cortical volumes, 4 subcortical volumes, and 10 areas) and RSFCs (5 network RSFCs, 2 cortico-subcortical RSFCs, *p*_fdr_ < 0.05). To test the robustness of our results in LMMs, randomly select 80% of samples and run the same procedures of LMMs 500 times (sampling LMMs) for PFI and DFI. For each resampling LMM, significant variables can be calculated in each variable set (*p*_fdr_ < 0.05). Repeat this procedure for all sampling LMMs. Subsequently, the percentage of significant variables from the primary LMMs that replicated significantly in the sampling LMMs was calculated. Additionally, the average and SD of the *t*-value and R2 s*p* of sampling LMMs were also included. Abbreviation: AN = auditory network; VN = visual network; SHN = sensorimotor hand network; SMN = sensorimotor mouth network; CON = cingulo-opercular network; CPN = cingulo-parietal network; DAN = dorsal attention network; DMN = default mode network; FPN = fronto-parietal network; RTN = retrosplenial temporal network; SN = salience network; Vtdc = ventral diencephalon; Amg = amygdala; Cde = caudate.

| **Variables** | ***t*-value** | ***t*-value (samplings)**  **mean (SD)** | **R2 s*p*** | **R2 s*p***  **(samplings)**  **mean (SD)** | **Percentage of replications (samplings)** |
| --- | --- | --- | --- | --- | --- |
| Picture vocabulary - Neurocognition | -2.25 | -2.15 (0.47) | 7.60E-04 | 7.6e-04 (3.2e-04) | 55.20% |
| Picture sequence memory - Neurocognition | 2.39 | 2.16 (0.46) | 8.00E-04 | 8.0e-04 (3.3e-04) | 57.60% |
| Anxious/Depressed problems - CBCL | -5.81 | -5.16 (0.46) | 4.30E-03 | 4.3e-03 (7.6e-04) | 100% |
| Withdrawn/Depressed problems - CBCL | -11.83 | -10.63 (0.46) | 1.80E-02 | 1.8e-02 (1.5e-03) | 100% |
| Somatic complaint problems - CBCL | -3.23 | -2.86 (0.47) | 1.40E-03 | 1.4e-03 (4.4e-04) | 94.80% |
| Social problems - CBCL | -7.7 | -6.87 (0.45) | 7.60E-03 | 7.6e-03 (9.9e-04) | 100% |
| Thought problems - CBCL | -7.57 | -6.77 (0.42) | 7.40E-03 | 7.4e-03 (9.1e-04) | 100% |
| Attention problems - CBCL | -10.47 | -9.37 (0.46) | 1.40E-02 | 1.4e-02 (1.4e-03) | 100% |
| Rule-Breaking problems - CBCL | -3.13 | -2.8 (0.46) | 1.30E-03 | 1.3e-03 (4.2e-04) | 93.20% |
| Aggressive problems - CBCL | -2.67 | -2.34 (0.46) | 9.20E-04 | 9.2e-04 (3.5e-04) | 70.60% |
| Internalizing problems - CBCL | -8.29 | -7.4 (0.47) | 8.60E-03 | 8.6e-03 (1.1e-03) | 100% |
| Externalizing problems - CBCL | -3 | -2.66 (0.46) | 1.20E-03 | 1.2e-03 (3.9e-04) | 89.20% |
| Total problems - CBCL | -8.39 | -7.48 (0.46) | 8.70E-03 | 8.7e-03 (1.1e-03) | 100% |
| Depression - CBCL | -9.2 | -8.21 (0.45) | 1.10E-02 | 1.1e-02 (1.2e-03) | 100% |
| Anxiety - CBCL | -6.68 | -5.96 (0.46) | 5.70E-03 | 5.7e-03 (8.7e-04) | 100% |
| ADHD - CBCL | -7.69 | -6.86 (0.47) | 7.60E-03 | 7.6e-03 (1.0e-03) | 100% |
| Oppositional defiant problems - CBCL | -2.61 | -2.31 (0.47) | 8.90E-04 | 8.9e-04 (3.4e-04) | 69.60% |
| Conduct problems - CBCL | -3.02 | -2.68 (0.46) | 1.20E-03 | 1.2e-03 (4.0e-04) | 90% |
| Sluggish cognitive tempo - CBCL | -11.73 | -10.51 (0.42) | 1.80E-02 | 1.8e-02 (1.4e-03) | 100% |
| OCD - CBCL | -5.75 | -5.12 (0.45) | 4.30E-03 | 4.3e-03 (7.5e-04) | 100% |
| Stress problems - CBCL | -7.62 | -6.78 (0.45) | 7.30E-03 | 7.3e-03 (9.6e-04) | 100% |
| Total events - Life Events (Parent) | -4.41 | -4 (0.5) | 1.90E-03 | 1.9e-03 (5.3e-04) | 100% |
| Bad events - Life Events (Parent) | -2.39 | -2.09 (0.49) | 5.90E-04 | 5.9e-04 (2.7e-04) | 47.80% |
| Bad affections - Life Events (Parent) | -2.11 | -1.82 (0.48) | 4.70E-04 | 4.7e-04 (2.4e-04) | 32% |
| Total affections - Life Events (Parent) | -3.33 | -2.94 (0.48) | 1.10E-03 | 1.1e-03 (3.7e-04) | 95.80% |
| Total events - Life Events | -3.27 | -2.96 (0.44) | 1.40E-03 | 1.4e-03 (4.0e-04) | 98% |
| Good events - Life Events | 2.64 | 2.4 (0.45) | 9.50E-04 | 9.5e-04 (3.4e-04) | 78.60% |
| Good affections - Life Events | 3.53 | 3.18 (0.44) | 1.60E-03 | 1.6e-03 (4.5e-04) | 99.40% |
| Total score - Prodromal Psychosis | -9.1 | -8.18 (0.46) | 1.10E-02 | 1.1e-02 (1.2e-03) | 100% |
| Distress score - Prodromal Psychosis | -7.29 | -6.54 (0.47) | 7.00E-03 | 7.0e-03 (9.8e-04) | 100% |
| Negative urgency - Impulsivity | -9.09 | -8.18 (0.48) | 1.10E-02 | 1.1e-02 (1.3e-03) | 100% |
| Lack of planning - Impulsivity | -10.05 | -9.05 (0.5) | 1.30E-02 | 1.3e-02 (1.4e-03) | 100% |
| Sensation seeking - Impulsivity | 8.69 | 7.83 (0.44) | 9.90E-03 | 9.9e-03 (1.1e-03) | 100% |
| Positive urgency - Impulsivity | -6.07 | -5.42 (0.44) | 4.80E-03 | 4.8e-03 (7.7e-04) | 100% |
| Lack of perseverance - Impulsivity | -22.27 | -20 (0.48) | 6.10E-02 | 6.1e-02 (2.7e-03) | 100% |
| BIS sum score - Inhibition and Reward-seeking | -2.06 | -1.88 (0.46) | 6.10E-04 | 6.1e-04 (2.8e-04) | 34.60% |
| BAS reward responsiveness - Inhibition and Reward-seeking | 8.22 | 7.39 (0.45) | 8.90E-03 | 8.9e-03 (1.1e-03) | 100% |
| BAS drive - Inhibition and Reward-seeking | 7.02 | 6.25 (0.46) | 6.40E-03 | 6.4e-03 (9.3e-04) | 100% |
| BAS fun seeking - Inhibition and Reward-seeking | 6.79 | 6.07 (0.48) | 6.00E-03 | 6.0e-03 (9.3e-04) | 100% |
| BIS sum score (modified) - Inhibition and Reward-seeking | -2.18 | -2 (0.47) | 6.90E-04 | 6.9e-04 (3.1e-04) | 45.20% |
| BAS reward responsiveness (modified) - Inhibition and Reward-seeking | 8.44 | 7.58 (0.46) | 9.30E-03 | 9.3e-03 (1.1e-03) | 100% |
| BAS drive (modified) - Inhibition and Reward-seeking | 7.02 | 6.25 (0.46) | 6.40E-03 | 6.4e-03 (9.3e-04) | 100% |
| Relational victimization - Adverse Peer Experiences | -4.12 | -3.72 (0.46) | 2.30E-03 | 2.3e-03 (5.5e-04) | 99.80% |
| Reputational aggression - Adverse Peer Experiences | -2.21 | -1.96 (0.47) | 6.60E-04 | 6.6e-04 (3.1e-04) | 40% |
| Reputational victimization - Adverse Peer Experiences | -3.23 | -2.95 (0.44) | 1.40E-03 | 1.4e-03 (4.2e-04) | 97% |
| Overt aggression - Adverse Peer Experiences | -4.94 | -4.43 (0.46) | 3.20E-03 | 3.2e-03 (6.6e-04) | 100% |
| Overt victimization - Adverse Peer Experiences | -5.67 | -5.1 (0.45) | 4.30E-03 | 4.3e-03 (7.4e-04) | 100% |
| Relational aggression - Adverse Peer Experiences | -2.53 | -2.26 (0.44) | 8.70E-04 | 8.7e-04 (3.2e-04) | 68.80% |
| Inferior Parietal - Left (Volume) | 3.6 | 3.29 (0.46) | 2.10E-03 | 2.1e-03 (5.9e-04) | 78% |
| Paracentral - Left (Volume) | 3.16 | 2.85 (0.45) | 1.60E-03 | 1.6e-03 (5.0e-04) | 54.60% |
| Precentral – Left (Volume) | 3.96 | 3.57 (0.42) | 2.40E-03 | 2.4e-03 (5.6e-04) | 87.40% |
| Rostral Anterior Cingulate – Left (Volume) | 2.59 | 2.33 (0.46) | 1.10E-03 | 1.1e-03 (4.3e-04) | 22.80% |
| Superior Frontal - Left (Volume) | 3.32 | 2.97 (0.47) | 1.60E-03 | 1.6e-03 (4.8e-04) | 62.40% |
| Insula - Left (Volume) | 2.62 | 2.37 (0.45) | 1.00E-03 | 1.0e-03 (3.9e-04) | 25.60% |
| Bankssts - Left (Volume) | 2.63 | 2.41 (0.45) | 1.10E-03 | 1.1e-03 (4.1e-04) | 25.40% |
| Inferior Parietal - Right (Volume) | 3.02 | 2.76 (0.43) | 1.40E-03 | 1.4e-03 (4.4e-04) | 48.40% |
| Middle Temporal - Right (Volume) | 2.67 | 2.43 (0.45) | 1.10E-03 | 1.1e-03 (3.9e-04) | 28.60% |
| Postcentral - Right (Volume) | 2.95 | 2.63 (0.44) | 1.30E-03 | 1.3e-03 (4.4e-04) | 41.20% |
| Precentral - Right (Volume) | 3.04 | 2.77 (0.44) | 1.40E-03 | 1.4e-03 (4.4e-04) | 49.80% |
| Superior Frontal - Right (Volume) | 2.81 | 2.52 (0.46) | 1.10E-03 | 1.1e-03 (3.8e-04) | 34.40% |
| Frontal Pole - Right (Volume) | 3.29 | 2.95 (0.45) | 1.70E-03 | 1.7e-03 (5.1e-04) | 58.60% |
| Insula - Right (Volume) | 3.04 | 2.73 (0.44) | 1.50E-03 | 1.5e-03 (4.6e-04) | 45.20% |
| Inferior Parietal - Left (Area) | 3.04 | 3.38 (0.44) | 2.20E-03 | 2.2e-03 (5.7e-04) | 36.80% |
| Paracentral - Left (Area) | 2.75 | 2.67 (0.44) | 1.40E-03 | 1.4e-03 (4.6e-04) | 21% |
| Postcentral - Left (Area) | 3.15 | 3.48 (0.46) | 2.40E-03 | 2.4e-03 (6.3e-04) | 40% |
| Precentral - Left (Area) | 3.26 | 2.34 (0.47) | 1.10E-03 | 1.1e-03 (4.3e-04) | 46.60% |
| Insula - Left (Area) | 3.28 | 2.77 (0.46) | 1.40E-03 | 1.4e-03 (4.6e-04) | 45.80% |
| Postcentral - Right (Area) | 3.2 | 2.46 (0.45) | 1.20E-03 | 1.2e-03 (4.2e-04) | 44.80% |
| Posterior Cingulate - Right (Area) | 2.73 | 2.81 (0.44) | 1.20E-03 | 1.2e-03 (3.8e-04) | 19.40% |
| Precentral - Right (Area) | 2.77 | 2.92 (0.42) | 1.30E-03 | 1.3e-03 (3.7e-04) | 26% |
| Frontal Pole - Right (Area) | 3.19 | 2.95 (0.46) | 1.70E-03 | 1.7e-03 (5.4e-04) | 42.80% |
| Insula - Right (Area) | 2.93 | 2.88 (0.46) | 1.30E-03 | 1.3e-03 (3.9e-04) | 31.20% |
| Putamen - Left (Subcortical Volume) | 3.74 | 2.43 (0.45) | 1.10E-03 | 1.1e-03 (4.2e-04) | 92.60% |
| Putamen - Right (Subcortical Volume) | 2.93 | 2.51 (0.43) | 9.70E-04 | 9.7e-04 (3.3e-04) | 62.40% |
| Pallidum - Right (Subcortical Volume) | 3.84 | 2.85 (0.45) | 1.60E-03 | 1.6e-03 (4.9e-04) | 94% |
| NAc - Right (Subcortical Volume) | 2.54 | 2.64 (0.44) | 1.40E-03 | 1.4e-03 (4.4e-04) | 38.20% |
| AN_CPN (RSFCs) | -3.59 | -3.22 (0.45) | 2.20E-03 | 2.2e-03 (6.1e-04) | 53.20% |
| CON_DAN (RSFCs) | 3.12 | 2.78 (0.43) | 1.70E-03 | 1.7e-03 (5.1e-04) | 26% |
| CPN_SMN (RSFCs) | -3.33 | -2.97 (0.46) | 1.90E-03 | 1.9e-03 (5.8e-04) | 41% |
| CPN_VN (RSFCs) | -3.17 | -2.81 (0.46) | 1.70E-03 | 1.7e-03 (5.5e-04) | 28.20% |
| DMN_FPN (RSFCs) | -3.22 | -2.88 (0.46) | 1.80E-03 | 1.8e-03 (5.6e-04) | 32.40% |
| DMN-Vtdc (Cortico-subcortical RSFCs) | 3.44 | 3.07 (0.44) | 1.80E-03 | 1.8e-03 (5.0e-04) | 22% |
| SMN-Cde (Cortico-subcortical RSFCs) | 3.38 | 3.04 (0.42) | 2.00E-03 | 2.0e-03 (5.3e-04) | 19% |

**Supplementary Table 4 |** Significant results of association analyses between delinquent friends index (DFI) and behavioral (41 behavioral-cognitive variables) and brain structures (3 cortical volumes, 3 cortical thickness) and RSFCs (12 network RSFCs, 46 cortico-subcortical RSFCs, *p*_fdr_ < 0.05). As described in Supplementary Table 3, the average and SD of the *t*-value and R2 s*p*, and the percentage of significant variables from the primary LMMs that replicated significantly in the sampling LMMs were included. Abbreviation: AN = auditory network; VN = visual network; SHN = sensorimotor hand network; SMN = sensorimotor mouth network; CON = cingulo-opercular network; CPN = cingulo-parietal network; DAN = dorsal attention network; DMN = default mode network; FPN = fronto-parietal network; RTN = retrosplenial temporal network; SN = salience network; VAN = ventral attention network; Crcx = cerebellum cortex; Tha = thalamus; Hip = hippocampus; Amg = amygdala; Pt = putamen; Pl = pallidum; Cde = caudate; NAc = nucleus accumbens; Vtdc = ventral diencephalon; BS = brain-stem.

| **Variables** | ***t*-value** | ***t*-value (samplings)**  **mean (SD)** | **R2 s*p*** | **R2 s*p***  **(samplings)**  **mean (SD)** | **Percentage of replications (samplings)** |
| --- | --- | --- | --- | --- | --- |
| Picture vocabulary - Neurocognition | -11.87 | -10.81 (0.43) | 1.80E-02 | 1.8e-02 (1.4e-03) | 100% |
| Picture sequence memory - Neurocognition | -7.96 | -7.22 (0.45) | 8.40E-03 | 8.4e-03 (1.0e-03) | 100% |
| Oral reading recognition - Neurocognition | -9.43 | -8.5 (0.46) | 1.10E-02 | 1.1e-02 (1.2e-03) | 100% |
| Social problems - CBCL | 5.07 | 4.6 (0.56) | 3.40E-03 | 3.4e-03 (8.2e-04) | 100% |
| Thought problems - CBCL | 4.83 | 4.39 (0.54) | 3.10E-03 | 3.1e-03 (7.5e-04) | 100% |
| Attention problems - CBCL | 10.07 | 9.07 (0.57) | 1.30E-02 | 1.3e-02 (1.6e-03) | 100% |
| Rule-Breaking problems - CBCL | 17.19 | 15.59 (0.73) | 3.80E-02 | 3.8e-02 (3.4e-03) | 100% |
| Aggressive problems - CBCL | 11.46 | 10.34 (0.6) | 1.70E-02 | 1.7e-02 (1.9e-03) | 100% |
| Externalizing problems - CBCL | 14.07 | 12.74 (0.65) | 2.50E-02 | 2.5e-02 (2.5e-03) | 100% |
| Total problems - CBCL | 8.74 | 7.97 (0.6) | 9.80E-03 | 9.8e-03 (1.5e-03) | 100% |
| Depression - CBCL | 3.34 | 3.09 (0.53) | 1.60E-03 | 1.6e-03 (5.1e-04) | 96.60% |
| ADHD - CBCL | 11 | 9.88 (0.57) | 1.60E-02 | 1.6e-02 (1.8e-03) | 100% |
| Oppositional defiant problems - CBCL | 10.86 | 9.83 (0.53) | 1.50E-02 | 1.5e-02 (1.6e-03) | 100% |
| Conduct problems - CBCL | 15.97 | 14.49 (0.75) | 3.30E-02 | 3.3e-02 (3.3e-03) | 100% |
| Sluggish cognitive tempo - CBCL | 2.27 | 2.08 (0.55) | 7.50E-04 | 7.5e-04 (3.7e-04) | 50.80% |
| Stress problems - CBCL | 5.86 | 5.33 (0.55) | 4.50E-03 | 4.5e-03 (9.1e-04) | 100% |
| Sum score - Subsyndromal Mania (Parent) | 8.51 | 7.63 (0.73) | 9.30E-03 | 9.3e-03 (1.7e-03) | 100% |
| Bad events - Life Events (Parent) | 4.82 | 4.52 (0.61) | 2.60E-03 | 2.6e-03 (6.9e-04) | 100% |
| Bad affections - Life Events (Parent) | 4.86 | 4.6 (0.62) | 2.80E-03 | 2.8e-03 (7.5e-04) | 100% |
| Mean affections - Life Events (Parent) | 2.22 | 2.09 (0.52) | 6.60E-04 | 6.6e-04 (3.1e-04) | 50.40% |
| Total affections - Life Events (Parent) | 2.87 | 2.79 (0.6) | 1.00E-03 | 1.0e-03 (4.3e-04) | 89% |
| Total events - Life Events | 12.99 | 11.64 (0.59) | 2.00E-02 | 2.0e-02 (2.0e-03) | 100% |
| Bad events - Life Events | 14.03 | 12.62 (0.61) | 2.50E-02 | 2.5e-02 (2.4e-03) | 100% |
| Total affections - Life Events | 10.47 | 9.37 (0.61) | 1.40E-02 | 1.4e-02 (1.7e-03) | 100% |
| Bad affections - Life Events | 13.46 | 12.09 (0.64) | 2.30E-02 | 2.3e-02 (2.4e-03) | 100% |
| Total score - Prodromal Psychosis | 16.68 | 14.95 (0.61) | 3.50E-02 | 3.5e-02 (2.7e-03) | 100% |
| Distress score - Prodromal Psychosis | 14.96 | 13.43 (0.63) | 2.80E-02 | 2.8e-02 (2.6e-03) | 100% |
| Negative urgency - Impulsivity | 14.84 | 13.36 (0.49) | 2.80E-02 | 2.8e-02 (2.0e-03) | 100% |
| Lack of planning - Impulsivity | 13.68 | 12.21 (0.58) | 2.30E-02 | 2.3e-02 (2.2e-03) | 100% |
| Sensation seeking - Impulsivity | 8.27 | 7.4 (0.44) | 8.80E-03 | 8.8e-03 (1.0e-03) | 100% |
| Positive urgency - Impulsivity | 15.07 | 13.54 (0.5) | 2.90E-02 | 2.9e-02 (2.1e-03) | 100% |
| Lack of perseverance - Impulsivity | 11.74 | 10.51 (0.54) | 1.80E-02 | 1.8e-02 (1.8e-03) | 100% |
| BAS drive - Inhibition and Reward-seeking | 8.33 | 7.52 (0.5) | 9.10E-03 | 9.1e-03 (1.2e-03) | 100% |
| BAS fun seeking - Inhibition and Reward-seeking | 10.54 | 9.46 (0.52) | 1.40E-02 | 1.4e-02 (1.5e-03) | 100% |
| BAS drive (modified) - Inhibition and Reward-seeking | 8.33 | 7.52 (0.5) | 9.10E-03 | 9.1e-03 (1.2e-03) | 100% |
| Relational victimization - Adverse Peer Experiences | 8.18 | 7.33 (0.51) | 8.70E-03 | 8.7e-03 (1.2e-03) | 100% |
| Reputational aggression - Adverse Peer Experiences | 18.94 | 16.99 (1.05) | 4.50E-02 | 4.5e-02 (5.2e-03) | 100% |
| Reputational victimization - Adverse Peer Experiences | 14.66 | 13.18 (0.62) | 2.70E-02 | 2.7e-02 (2.5e-03) | 100% |
| Overt aggression - Adverse Peer Experiences | 21.09 | 18.94 (0.89) | 5.50E-02 | 5.5e-02 (4.9e-03) | 100% |
| Overt victimization - Adverse Peer Experiences | 16.1 | 14.43 (0.69) | 3.30E-02 | 3.3e-02 (3.0e-03) | 100% |
| Relational aggression - Adverse Peer Experiences | 15.76 | 14.14 (0.63) | 3.10E-02 | 3.1e-02 (2.7e-03) | 100% |
| Lateral Occipital - Left (Volume) | -3.76 | -3.39 (0.43) | 2.30E-03 | 2.3e-03 (5.7e-04) | 62% |
| Lateral Occipital - Right (Volume) | -3.14 | -2.89 (0.43) | 1.70E-03 | 1.7e-03 (4.9e-04) | 31.80% |
| Middle Temporal - Right (Volume) | -3.21 | -2.98 (0.46) | 1.60E-03 | 1.6e-03 (4.7e-04) | 39.20% |
| Lateral Occipital - Left (Thickness) | -3.41 | -2.83 (0.43) | 1.60E-03 | 1.6e-03 (4.9e-04) | 55.20% |
| Superior Frontal - Left (Thickness) | -3.14 | -3.11 (0.46) | 1.60E-03 | 1.6e-03 (4.6e-04) | 39.60% |
| Lateral Occipital - Right (Thickness) | -4.05 | -2.86 (0.46) | 1.60E-03 | 1.6e-03 (5.2e-04) | 77% |
| Hippocampus (Subcortical Volume) | -3.09 | -3.64 (0.46) | 2.20E-03 | 2.2e-03 (5.6e-04) | 39.80% |
| AN-AN (RSFCs) | 2.85 | 2.54 (0.49) | 1.40E-03 | 1.4e-03 (5.1e-04) | 37% |
| DMN-DMN (RSFCs) | -3.88 | -3.49 (0.44) | 2.30E-03 | 2.3e-03 (5.7e-04) | 92% |
| DAN-DAN (RSFCs) | -3.61 | -3.24 (0.45) | 2.20E-03 | 2.2e-03 (6.2e-04) | 84.20% |
| SHN-SHN (RSFCs) | 6.13 | 5.56 (0.44) | 6.40E-03 | 6.4e-03 (1.0e-03) | 100% |
| AN-SHN (RSFCs) | 3.56 | 3.24 (0.45) | 2.20E-03 | 2.2e-03 (6.0e-04) | 86.40% |
| CON-CPN (RSFCs) | -3.09 | -2.83 (0.44) | 1.70E-03 | 1.7e-03 (5.1e-04) | 60.40% |
| CON-DMN (RSFCs) | 2.96 | 2.64 (0.45) | 1.40E-03 | 1.4e-03 (4.8e-04) | 43% |
| CON-VN (RSFCs) | -3.43 | -3.08 (0.44) | 2.00E-03 | 2.0e-03 (5.7e-04) | 75.80% |
| DMN-DAN (RSFCs) | 4.14 | 3.75 (0.47) | 2.80E-03 | 2.8e-03 (7.0e-04) | 96.20% |
| DAN-VAN (RSFCs) | 3.44 | 3.05 (0.44) | 1.90E-03 | 1.9e-03 (5.5e-04) | 75% |
| FPN-SHN (RSFCs) | -2.71 | -2.48 (0.46) | 1.30E-03 | 1.3e-03 (4.7e-04) | 31.60% |
| SHN-SMN (RSFCs) | 4.71 | 4.3 (0.45) | 3.80E-03 | 3.8e-03 (8.0e-04) | 99.80% |
| AN-NAc (Cortico-subcortical RSFCs) | -3.08 | -2.76 (0.46) | 1.60E-03 | 1.6e-03 (5.3e-04) | 79.20% |
| AN-Amg (Cortico-subcortical RSFCs) | 2.54 | 2.27 (0.49) | 1.10E-03 | 1.1e-03 (4.7e-04) | 41.80% |
| AN-Cde (Cortico-subcortical RSFCs) | -2.43 | -2.14 (0.47) | 1.00E-03 | 1.0e-03 (4.2e-04) | 30.60% |
| AN-Hip (Cortico-subcortical RSFCs) | 3.43 | 3.1 (0.48) | 2.00E-03 | 2.0e-03 (6.2e-04) | 92.60% |
| AN-Vtdc (Cortico-subcortical RSFCs) | -2.47 | -2.2 (0.48) | 1.00E-03 | 1.0e-03 (4.4e-04) | 37% |
| CON-Amg (Cortico-subcortical RSFCs) | -4.94 | -4.51 (0.46) | 4.20E-03 | 4.2e-03 (8.4e-04) | 100% |
| CON-Cde (Cortico-subcortical RSFCs) | -2.58 | -2.34 (0.47) | 1.20E-03 | 1.2e-03 (4.6e-04) | 46.40% |
| CON-Hip (Cortico-subcortical RSFCs) | -4.59 | -4.17 (0.43) | 3.50E-03 | 3.5e-03 (7.4e-04) | 100% |
| CON-Pt (Cortico-subcortical RSFCs) | -4.25 | -3.81 (0.43) | 3.00E-03 | 3.0e-03 (6.7e-04) | 99.80% |
| CON-Tha (Cortico-subcortical RSFCs) | -4.13 | -3.72 (0.49) | 2.90E-03 | 2.9e-03 (7.5e-04) | 99.20% |
| CON-Vtdc (Cortico-subcortical RSFCs) | 2.59 | 2.33 (0.46) | 1.20E-03 | 1.2e-03 (4.5e-04) | 47% |
| CPN-Cde (Cortico-subcortical RSFCs) | -2.93 | -2.66 (0.46) | 1.50E-03 | 1.5e-03 (5.1e-04) | 70.40% |
| CPN-Crcx (Cortico-subcortical RSFCs) | -4.63 | -4.16 (0.46) | 3.70E-03 | 3.7e-03 (8.0e-04) | 99.80% |
| CPN-Vtdc (Cortico-subcortical RSFCs) | -4 | -3.64 (0.49) | 2.80E-03 | 2.8e-03 (7.5e-04) | 99% |
| DMN-NAc (Cortico-subcortical RSFCs) | -3.86 | -3.47 (0.46) | 2.40E-03 | 2.4e-03 (6.1e-04) | 98.40% |
| DMN-Amg (Cortico-subcortical RSFCs) | -3.87 | -3.49 (0.48) | 2.60E-03 | 2.6e-03 (7.0e-04) | 98.40% |
| DMN-Pl (Cortico-subcortical RSFCs) | -3.44 | -3.07 (0.45) | 2.00E-03 | 2.0e-03 (5.6e-04) | 92.20% |
| DAN-Amg (Cortico-subcortical RSFCs) | 2.81 | 2.59 (0.43) | 1.40E-03 | 1.4e-03 (4.6e-04) | 66.80% |
| DAN-Hip (Cortico-subcortical RSFCs) | 3.01 | 2.72 (0.44) | 1.60E-03 | 1.6e-03 (4.9e-04) | 77.80% |
| DAN-Vtdc (Cortico-subcortical RSFCs) | -2.97 | -2.7 (0.43) | 1.50E-03 | 1.5e-03 (4.7e-04) | 75.40% |
| FPN-Amg (Cortico-subcortical RSFCs) | -3.15 | -2.84 (0.41) | 1.70E-03 | 1.7e-03 (4.8e-04) | 86% |
| FPN-Cde (Cortico-subcortical RSFCs) | -2.61 | -2.36 (0.46) | 1.10E-03 | 1.1e-03 (4.2e-04) | 51% |
| FPN-Crcx (Cortico-subcortical RSFCs) | 3.51 | 3.19 (0.45) | 2.10E-03 | 2.1e-03 (6.0e-04) | 97.20% |
| FPN-Tha (Cortico-subcortical RSFCs) | 3.81 | 3.43 (0.43) | 2.50E-03 | 2.5e-03 (6.2e-04) | 99.60% |
| RTN-NAc (Cortico-subcortical RSFCs) | -3.65 | -3.29 (0.48) | 2.20E-03 | 2.2e-03 (6.3e-04) | 97% |
| RTN-Hip (Cortico-subcortical RSFCs) | -2.63 | -2.38 (0.43) | 1.20E-03 | 1.2e-03 (4.3e-04) | 51.60% |
| RTN-Vtdc (Cortico-subcortical RSFCs) | -2.87 | -2.59 (0.47) | 1.40E-03 | 1.4e-03 (5.0e-04) | 65.60% |
| SN-NAc (Cortico-subcortical RSFCs) | -3.03 | -2.72 (0.45) | 1.50E-03 | 1.5e-03 (4.8e-04) | 75.80% |
| SN-Hip (Cortico-subcortical RSFCs) | 3.2 | 2.91 (0.47) | 1.80E-03 | 1.8e-03 (5.7e-04) | 85.20% |
| SN-Tha (Cortico-subcortical RSFCs) | -2.48 | -2.22 (0.49) | 1.00E-03 | 1.0e-03 (4.2e-04) | 38.40% |
| SN-Vtdc (Cortico-subcortical RSFCs) | 4.13 | 3.74 (0.44) | 2.90E-03 | 2.9e-03 (6.8e-04) | 99.60% |
| SHN-NAc (Cortico-subcortical RSFCs) | -4.68 | -4.23 (0.47) | 3.80E-03 | 3.8e-03 (8.3e-04) | 100% |
| SHN-Cde (Cortico-subcortical RSFCs) | -4.4 | -4.01 (0.48) | 3.30E-03 | 3.3e-03 (8.0e-04) | 100% |
| SHN-Crcx (Cortico-subcortical RSFCs) | -3.39 | -3.04 (0.43) | 1.90E-03 | 1.9e-03 (5.3e-04) | 91% |
| SHN-Pl (Cortico-subcortical RSFCs) | -4.38 | -3.9 (0.42) | 3.20E-03 | 3.2e-03 (6.6e-04) | 100% |
| SHN-Pt (Cortico-subcortical RSFCs) | -5.6 | -5.07 (0.45) | 5.20E-03 | 5.2e-03 (9.7e-04) | 100% |
| SMN-Amg (Cortico-subcortical RSFCs) | -3.58 | -3.27 (0.45) | 2.30E-03 | 2.3e-03 (6.2e-04) | 96.20% |
| SMN-Hip (Cortico-subcortical RSFCs) | -4.3 | -3.91 (0.47) | 3.20E-03 | 3.2e-03 (7.6e-04) | 100% |
| SMN-Pl (Cortico-subcortical RSFCs) | 3.22 | 2.89 (0.43) | 1.70E-03 | 1.7e-03 (5.0e-04) | 88.40% |
| VAN-Cde (Cortico-subcortical RSFCs) | -3.57 | -3.23 (0.44) | 2.20E-03 | 2.2e-03 (5.7e-04) | 96.20% |
| VN-Hip (Cortico-subcortical RSFCs) | -3.46 | -3.1 (0.46) | 2.00E-03 | 2.0e-03 (6.0e-04) | 92.80% |
| VN-Pl (Cortico-subcortical RSFCs) | -2.87 | -2.57 (0.49) | 1.40E-03 | 1.4e-03 (5.4e-04) | 62.20% |
| CON-BS (Cortico-subcortical RSFCs) | -4.81 | -4.29 (0.48) | 3.90E-03 | 3.9e-03 (8.5e-04) | 100% |
| DAN-BS (Cortico-subcortical RSFCs) | -3.23 | -2.92 (0.43) | 1.70E-03 | 1.7e-03 (5.0e-04) | 87.80% |
| RTN-BS (Cortico-subcortical RSFCs) | -4.34 | -3.94 (0.49) | 3.30E-03 | 3.3e-03 (8.0e-04) | 99.60% |
| SHN-BS (Cortico-subcortical RSFCs) | -3.59 | -3.23 (0.44) | 2.20E-03 | 2.2e-03 (5.7e-04) | 96.20% |

**Supplementary Table 5 |** The significant results of mediation analyses of peer environments. Brain features (significant with PFI/DFI in LMMs) were mediators, PFI/DFI were independent variables, and behavioral-cognitive variables were dependent variables. Due to space limitations, only the associations that were significantly mediated by peer environments were presented (*p*_fdr_ < 0.05 for each path). For brain volumes, 41 mediation effects were found between PFI and behavioral-cognitive variables. And 28 mediation effects for brain volumes were found in the relationships between DFI and behavioral-cognitive variables. For network RSFCs, 44 mediation effects were found between DFI and behavioral variables. For cortico-subcortical RSFCs, 199 mediation effects were found between DFI and behavioral-cognitive variables. Abbreviation: AN = auditory network; VN = visual network; SHN = sensorimotor hand network; SMN = sensorimotor mouth network; CON = cingulo-opercular network; CPN = cingulo-parietal network; DAN = dorsal attention network; DMN = default mode network; FPN = fronto-parietal network; RTN = retrosplenial temporal network; SN = salience network; VAN = ventral attention network.

| **Independent variables** | **Dependent variables** | **Mediator** | **PathA**  **(*β*)** | **PathB**  **(*β*)** | **PathAB**  **(*β*)** | **PathC**  **(*β*)** | **PathC’**  **(*β*)** | **Mediation Proportion** |
| --- | --- | --- | --- | --- | --- | --- | --- | --- |
| PFI | Reputational victimization - Adverse Peer Experiences | Inferior Parietal (L) | 39.125 | -3.97E-05 | -0.0016 | -0.0357 | -0.0341 | 4.35% |
| PFI | Picture vocabulary - Neurocognition | Parancentral (L) | 10.446 | 5.81E-04 | 0.0061 | -0.1301 | -0.1362 | 4.67% |
| PFI | Picture vocabulary - Neurocognition | Prencentral (L) | 32.84 | 4.45E-04 | 0.0146 | -0.1301 | -0.1447 | 11.24% |
| PFI | Anxious/Depressed problems - CBCL | Prencentral (L) | 32.84 | 8.20E-05 | 0.0027 | -0.0677 | -0.0704 | 3.98% |
| PFI | Anxiety - CBCL | Prencentral (L) | 32.84 | 6.70E-05 | 0.0022 | -0.0648 | -0.067 | 3.40% |
| PFI | Total score - Prodromal Psychosis | Prencentral (L) | 32.84 | -8.55E-05 | -0.0028 | -0.109 | -0.1062 | 2.58% |
| PFI | Picture vocabulary - Neurocognition | Superior Frontal (L) | 59.381 | 2.24E-04 | 0.0133 | -0.1301 | -0.1434 | 10.20% |
| PFI | Total events - Life Events (Parent) | Superior Frontal (L) | 59.381 | 3.68E-05 | 0.0022 | -0.0542 | -0.0564 | 4.03% |
| PFI | Lack of planning - Impulsivity | Superior Frontal (L) | 59.381 | -3.31E-05 | -0.002 | -0.1073 | -0.1053 | 1.83% |
| PFI | Picture vocabulary - Neurocognition | Insula (L) | 11.403 | 6.85E-04 | 0.0078 | -0.1301 | -0.1379 | 6% |
| PFI | Total events - Life Events (Parent) | Insula (L) | 11.403 | 1.88E-04 | 0.0021 | -0.0542 | -0.0563 | 3.96% |
| PFI | Total affections - Life Events (Parent) | Insula (L) | 11.403 | 3.54E-04 | 0.004 | -0.0775 | -0.0816 | 5.21% |
| PFI | Good events - Life Events | Insula (L) | 11.403 | 8.05E-05 | 9.00E-04 | 0.016 | 0.0151 | 5.72% |
| PFI | Good affections - Life Events | Insula (L) | 11.403 | 1.78E-04 | 0.002 | 0.044 | 0.042 | 4.61% |
| PFI | Picture vocabulary - Neurocognition | Inferior Parietal (R) | 40.955 | 1.88E-04 | 0.0077 | -0.1301 | -0.1378 | 5.92% |
| PFI | Total score - Prodromal Psychosis | Inferior Parietal (R) | 40.955 | -5.63E-05 | -0.0023 | -0.109 | -0.1067 | 2.11% |
| PFI | Distress score - Prodromal Psychosis | Inferior Parietal (R) | 40.955 | -1.33E-04 | -0.0055 | -0.2306 | -0.2251 | 2.37% |
| PFI | Lack of planning - Impulsivity | Inferior Parietal (R) | 40.955 | -4.49E-05 | -0.0018 | -0.1073 | -0.1055 | 1.71% |
| PFI | Overt aggression - Adverse Peer Experiences | Inferior Parietal (R) | 40.955 | -1.70E-05 | -7.00E-04 | -0.0276 | -0.027 | 2.52% |
| PFI | Picture vocabulary - Neurocognition | Prencentral (R) | 29.074 | 4.58E-04 | 0.0133 | -0.1301 | -0.1434 | 10.24% |
| PFI | Picture vocabulary - Neurocognition | Superior Frontal (R) | 48.277 | 2.51E-04 | 0.0121 | -0.1301 | -0.1422 | 9.31% |
| PFI | Picture vocabulary - Neurocognition | Frontal Pole (R) | 3.2169 | 0.001811 | 0.0058 | -0.1301 | -0.1359 | 4.48% |
| PFI | BAS drive - Inhibition and Reward-seeking | Frontal Pole (R) | 3.2169 | -6.74E-04 | -0.0022 | 0.0754 | 0.0776 | 2.87% |
| PFI | Picture vocabulary - Neurocognition | Insula (R) | 12.595 | 4.37E-04 | 0.0055 | -0.1301 | -0.1356 | 4.23% |
| PFI | Total events - Life Events (Parent) | Insula (R) | 12.595 | 1.82E-04 | 0.0023 | -0.0542 | -0.0565 | 4.22% |
| PFI | Total affections - Life Events (Parent) | Insula (R) | 12.595 | 2.98E-04 | 0.0038 | -0.0775 | -0.0813 | 4.84% |
| PFI | Good events - Life Events | Insula (R) | 12.595 | 7.85E-05 | 0.001 | 0.016 | 0.0151 | 6.16% |
| PFI | Picture vocabulary - Neurocognition | Putamen (L) | 8.6714 | 6.85E-04 | 0.0059 | -0.1301 | -0.136 | 4.57% |
| PFI | Attention problems - CBCL | Putamen (L) | 8.6714 | -2.88E-04 | -0.0025 | -0.142 | -0.1395 | 1.76% |
| PFI | BAS reward responsiveness - Inhibition and Reward-seeking | Putamen (L) | 8.6714 | 2.81E-04 | 0.0024 | 0.1046 | 0.1021 | 2.33% |
| PFI | BAS reward responsiveness (modified) - Inhibition and Reward-seeking | Putamen (L) | 8.6714 | 2.54E-04 | 0.0022 | 0.0876 | 0.0854 | 2.52% |
| PFI | Picture vocabulary - Neurocognition | Putamen (R) | 7.8473 | 5.85E-04 | 0.0046 | -0.1301 | -0.1347 | 3.53% |
| PFI | BAS reward responsiveness - Inhibition and Reward-seeking | Putamen (R) | 7.8473 | 3.16E-04 | 0.0025 | 0.1046 | 0.1021 | 2.37% |
| PFI | BAS reward responsiveness (modified) - Inhibition and Reward-seeking | Putamen (R) | 7.8473 | 2.78E-04 | 0.0022 | 0.0876 | 0.0855 | 2.49% |
| PFI | Negative urgency - Impulsivity | Pallidum (R) | 3.5388 | 5.92E-04 | 0.0021 | -0.0983 | -0.1004 | 2.13% |
| PFI | Sensation seeking - Impulsivity | Pallidum (R) | 3.5388 | 5.68E-04 | 0.002 | 0.0949 | 0.0929 | 2.12% |
| PFI | BAS reward responsiveness - Inhibition and Reward-seeking | Pallidum (R) | 3.5388 | 0.001055 | 0.0037 | 0.1046 | 0.1008 | 3.57% |
| PFI | BAS drive - Inhibition and Reward-seeking | Pallidum (R) | 3.5388 | 9.88E-04 | 0.0035 | 0.0754 | 0.0719 | 4.64% |
| PFI | BAS fun seeking - Inhibition and Reward-seeking | Pallidum (R) | 3.5388 | 6.71E-04 | 0.0024 | 0.0706 | 0.0683 | 3.36% |
| PFI | BAS reward responsiveness (modified) - Inhibition and Reward-seeking | Pallidum (R) | 3.5388 | 8.71E-04 | 0.0031 | 0.0876 | 0.0846 | 3.52% |
| PFI | BAS drive (modified) - Inhibition and Reward-seeking | Pallidum (R) | 3.5388 | 9.88E-04 | 0.0035 | 0.0754 | 0.0719 | 4.64% |
| PFI | Total events - Life Events | DMN-Ventral Diencephalon | 0.0014 | 1.9374 | 0.0026 | -0.0419 | -0.0445 | 6.28% |
| DFI | Bad events - Life Events (Parent) | Lateral Occipital (L) | -71.409 | 3.63E-05 | -0.0026 | 0.0893 | 0.0919 | 2.90% |
| DFI | Good affections - Life Events (Parent) | Lateral Occipital (L) | -71.409 | 6.82E-05 | -0.0049 | -0.0594 | -0.0545 | 8.21% |
| DFI | Bad affections - Life Events (Parent) | Lateral Occipital (L) | -71.409 | 8.41E-05 | -0.006 | 0.2121 | 0.2181 | 2.83% |
| DFI | Total affections - Life Events (Parent) | Lateral Occipital (L) | -71.409 | 1.90E-04 | -0.0136 | 0.1804 | 0.194 | 7.52% |
| DFI | BAS drive - Inhibition and Reward-seeking | Lateral Occipital (L) | -71.409 | -5.62E-05 | 0.004 | 0.2436 | 0.2396 | 1.65% |
| DFI | Overt aggression - Adverse Peer Experiences | Lateral Occipital (L) | -71.409 | -2.37E-05 | 0.0017 | 0.1864 | 0.1847 | 0.91% |
| DFI | Overt victimization - Adverse Peer Experiences | Lateral Occipital (L) | -71.409 | -3.31E-05 | 0.0024 | 0.21 | 0.2076 | 1.13% |
| DFI | Picture vocabulary - Neurocognition | Lateral Occipital (R) | -64.721 | 2.46E-04 | -0.0159 | -0.9153 | -0.8994 | 1.74% |
| DFI | Oral reading recognition - Neurocognition | Lateral Occipital (R) | -64.721 | 1.59E-04 | -0.0103 | -0.5185 | -0.5082 | 1.99% |
| DFI | Thought problems - CBCL | Lateral Occipital (R) | -64.721 | 4.95E-05 | -0.0032 | 0.1066 | 0.1098 | 3.01% |
| DFI | Depression - CBCL | Lateral Occipital (R) | -64.721 | 4.65E-05 | -0.003 | 0.0902 | 0.0932 | 3.34% |
| DFI | Bad events - Life Events (Parent) | Lateral Occipital (R) | -64.721 | 3.49E-05 | -0.0023 | 0.0893 | 0.0915 | 2.53% |
| DFI | Good affections - Life Events (Parent) | Lateral Occipital (R) | -64.721 | 6.92E-05 | -0.0045 | -0.0594 | -0.0549 | 7.54% |
| DFI | Mean affections - Life Events (Parent) | Lateral Occipital (R) | -64.721 | 1.90E-05 | -0.0012 | 0.0193 | 0.0205 | 6.36% |
| DFI | Total affections - Life Events (Parent) | Lateral Occipital (R) | -64.721 | 1.77E-04 | -0.0114 | 0.1804 | 0.1918 | 6.33% |
| DFI | BAS drive - Inhibition and Reward-seeking | Lateral Occipital (R) | -64.721 | -7.80E-05 | 0.005 | 0.2436 | 0.2386 | 2.07% |
| DFI | BAS drive (modified) - Inhibition and Reward-seeking | Lateral Occipital (R) | -64.721 | -7.80E-05 | 0.005 | 0.2436 | 0.2386 | 2.07% |
| DFI | Overt aggression - Adverse Peer Experiences | Lateral Occipital (R) | -64.721 | -2.34E-05 | 0.0015 | 0.1864 | 0.1848 | 0.81% |
| DFI | Picture vocabulary - Neurocognition | Middle Temporal (R) | -67.88 | 3.90E-04 | -0.0265 | -0.9153 | -0.8888 | 2.89% |
| DFI | Picture sequence memory - Neurocognition | Middle Temporal (R) | -67.88 | 4.19E-04 | -0.0285 | -1.0606 | -1.0322 | 2.68% |
| DFI | Oral reading recognition - Neurocognition | Middle Temporal (R) | -67.88 | 2.43E-04 | -0.0165 | -0.5185 | -0.502 | 3.19% |
| DFI | Total score - Prodromal Psychosis | Middle Temporal (R) | -67.88 | -6.46E-05 | 0.0044 | 0.4055 | 0.4012 | 1.08% |
| DFI | Sensation seeking - Impulsivity | Middle Temporal (R) | -67.88 | 7.40E-05 | -0.005 | 0.188 | 0.193 | 2.67% |
| DFI | Picture vocabulary - Neurocognition | Hippocampus (R) | -12.954 | 0.001465 | -0.019 | -0.9153 | -0.8963 | 2.07% |
| DFI | Picture sequence memory - Neurocognition | Hippocampus (R) | -12.954 | 0.001621 | -0.021 | -1.0606 | -1.0396 | 1.98% |
| DFI | Oral reading recognition - Neurocognition | Hippocampus (R) | -12.954 | 0.001197 | -0.0155 | -0.5185 | -0.503 | 2.99% |
| DFI | Rule-Breaking problems - CBCL | Hippocampus (R) | -12.954 | -1.88E-04 | 0.0024 | 0.3118 | 0.3093 | 0.78% |
| DFI | Conduct problems - CBCL | Hippocampus (R) | -12.954 | -2.43E-04 | 0.0032 | 0.3577 | 0.3545 | 0.88% |
| DFI | Picture vocabulary - Neurocognition | DMN-DMN | -0.0028 | 5.8142 | -0.0165 | -1.0014 | -0.9849 | 1.65% |
| DFI | Total score - Prodromal Psychosis | DMN-DMN | -0.0028 | -2.6299 | 0.0075 | 0.3808 | 0.3734 | 1.96% |
| DFI | Distress score - Prodromal Psychosis | DMN-DMN | -0.0028 | -5.5887 | 0.0159 | 1.0081 | 0.9922 | 1.57% |
| DFI | Picture vocabulary - Neurocognition | SHN-SHN | 0.007 | -7.2816 | -0.0508 | -1.0014 | -0.9506 | 5.07% |
| DFI | Social problems - CBCL | SHN-SHN | 0.007 | -1.0376 | -0.0072 | 0.1127 | 0.1199 | 6.42% |
| DFI | Thought problems - CBCL | SHN-SHN | 0.007 | -1.2324 | -0.0086 | 0.0947 | 0.1033 | 9.08% |
| DFI | Attention problems - CBCL | SHN-SHN | 0.007 | -1.8248 | -0.0127 | 0.3213 | 0.334 | 3.96% |
| DFI | Total problems - CBCL | SHN-SHN | 0.007 | -9.4219 | -0.0657 | 1.4931 | 1.5588 | 4.40% |
| DFI | Depression - CBCL | SHN-SHN | 0.007 | -1.2978 | -0.0091 | 0.0809 | 0.09 | 11.19% |
| DFI | Stress problems - CBCL | SHN-SHN | 0.007 | -2.0828 | -0.0145 | 0.1989 | 0.2134 | 7.30% |
| DFI | Good affections - Life Events (Parent) | SHN-SHN | 0.007 | -1.5616 | -0.0109 | -0.0713 | -0.0604 | 15.28% |
| DFI | Total affections - Life Events (Parent) | SHN-SHN | 0.007 | -2.6229 | -0.0183 | 0.1624 | 0.1807 | 11.27% |
| DFI | Sensation seeking - Impulsivity | SHN-SHN | 0.007 | 1.4757 | 0.0103 | 0.199 | 0.1887 | 5.17% |
| DFI | Positive urgency - Impulsivity | SHN-SHN | 0.007 | 2.2141 | 0.0154 | 0.3748 | 0.3593 | 4.12% |
| DFI | Reputational victimization - Adverse Peer Experiences | SHN-SHN | 0.007 | 0.75413 | 0.0053 | 0.2626 | 0.2573 | 2% |
| DFI | Picture vocabulary - Neurocognition | AN-SHN | 0.0035 | -9.9828 | -0.035 | -1.0014 | -0.9665 | 3.49% |
| DFI | Oral reading recognition - Neurocognition | AN-SHN | 0.0035 | -4.5323 | -0.0159 | -0.5717 | -0.5558 | 2.78% |
| DFI | Thought problems - CBCL | AN-SHN | 0.0035 | -1.4525 | -0.0051 | 0.0947 | 0.0998 | 5.37% |
| DFI | Good affections - Life Events (Parent) | AN-SHN | 0.0035 | -1.9843 | -0.0069 | -0.0713 | -0.0643 | 9.75% |
| DFI | Total affections - Life Events (Parent) | AN-SHN | 0.0035 | -3.3199 | -0.0116 | 0.1624 | 0.1741 | 7.16% |
| DFI | Positive urgency - Impulsivity | AN-SHN | 0.0035 | 2.1036 | 0.0074 | 0.3748 | 0.3674 | 1.97% |
| DFI | Picture vocabulary - Neurocognition | CON-CPN | -0.0029 | 8.7165 | -0.0252 | -1.0014 | -0.9762 | 2.51% |
| DFI | Oral reading recognition - Neurocognition | CON-CPN | -0.0029 | 5.2293 | -0.0151 | -0.5717 | -0.5566 | 2.64% |
| DFI | Total score - Prodromal Psychosis | CON-DMN | 0.0024 | 2.2322 | 0.0053 | 0.3808 | 0.3755 | 1.40% |
| DFI | Picture vocabulary - Neurocognition | CON-VN | -0.0023 | 5.4301 | -0.0127 | -1.0014 | -0.9887 | 1.27% |
| DFI | Oral reading recognition - Neurocognition | CON-VN | -0.0023 | 7.3924 | -0.0173 | -0.5717 | -0.5544 | 3.02% |
| DFI | Thought problems - CBCL | CON-VN | -0.0023 | -1.3778 | 0.0032 | 0.0947 | 0.0915 | 3.40% |
| DFI | Attention problems - CBCL | CON-VN | -0.0023 | -2.5356 | 0.0059 | 0.3213 | 0.3154 | 1.84% |
| DFI | ADHD - CBCL | CON-VN | -0.0023 | -2.1313 | 0.005 | 0.2848 | 0.2798 | 1.75% |
| DFI | Picture vocabulary - Neurocognition | DMN-DAN | 0.0029 | -8.3231 | -0.024 | -1.0014 | -0.9774 | 2.40% |
| DFI | Total score - Prodromal Psychosis | DMN-DAN | 0.0029 | 3.1031 | 0.009 | 0.3808 | 0.3719 | 2.35% |
| DFI | Distress score - Prodromal Psychosis | DMN-DAN | 0.0029 | 6.5564 | 0.0189 | 1.0081 | 0.9891 | 1.88% |
| DFI | Picture vocabulary - Neurocognition | FPN-SHN | -0.0018 | 7.5124 | -0.0138 | -1.0014 | -0.9876 | 1.38% |
| DFI | Good affections - Life Events (Parent) | FPN-SHN | -0.0018 | 2.4515 | -0.0045 | -0.0713 | -0.0668 | 6.32% |
| DFI | Picture vocabulary - Neurocognition | SHN-SMN | 0.0074 | -6.6022 | -0.0487 | -1.0014 | -0.9528 | 4.86% |
| DFI | Oral reading recognition - Neurocognition | SHN-SMN | 0.0074 | -2.7116 | -0.02 | -0.5717 | -0.5517 | 3.50% |
| DFI | Thought problems - CBCL | SHN-SMN | 0.0074 | -0.84391 | -0.0062 | 0.0947 | 0.1009 | 6.57% |
| DFI | Total problems - CBCL | SHN-SMN | 0.0074 | -6.9594 | -0.0513 | 1.4931 | 1.5444 | 3.44% |
| DFI | Depression - CBCL | SHN-SMN | 0.0074 | -1.0231 | -0.0075 | 0.0809 | 0.0885 | 9.32% |
| DFI | Stress problems - CBCL | SHN-SMN | 0.0074 | -1.5894 | -0.0117 | 0.1989 | 0.2106 | 5.89% |
| DFI | Good events - Life Events (Parent) | SHN-SMN | 0.0074 | -0.57689 | -0.0043 | -0.0473 | -0.0431 | 8.99% |
| DFI | Good affections - Life Events (Parent) | SHN-SMN | 0.0074 | -1.6134 | -0.0119 | -0.0713 | -0.0594 | 16.68% |
| DFI | Total affections - Life Events (Parent) | SHN-SMN | 0.0074 | -2.1063 | -0.0155 | 0.1624 | 0.178 | 9.56% |
| DFI | Positive urgency - Impulsivity | SHN-SMN | 0.0074 | 1.9138 | 0.0141 | 0.3748 | 0.3607 | 3.76% |
| DFI | Picture vocabulary - Neurocognition | AN-Hippocampus | 0.0027 | -9.1643 | -0.0249 | -1.0014 | -0.9765 | 2.49% |
| DFI | Oral reading recognition - Neurocognition | AN-Hippocampus | 0.0027 | -5.7907 | -0.0158 | -0.5717 | -0.556 | 2.76% |
| DFI | BAS drive - Inhibition and Reward-seeking | AN-Hippocampus | 0.0027 | 1.9306 | 0.0053 | 0.2357 | 0.2304 | 2.23% |
| DFI | BAS drive (modified) - Inhibition and Reward-seeking | AN-Hippocampus | 0.0027 | 1.9306 | 0.0053 | 0.2357 | 0.2304 | 2.23% |
| DFI | Picture vocabulary - Neurocognition | CON-Amygdala | -0.0062 | 7.552 | -0.0471 | -1.0014 | -0.9543 | 4.70% |
| DFI | Oral reading recognition - Neurocognition | CON-Amygdala | -0.0062 | 3.1611 | -0.0197 | -0.5717 | -0.552 | 3.45% |
| DFI | Thought problems - CBCL | CON-Amygdala | -0.0062 | 1.2896 | -0.008 | 0.0947 | 0.1028 | 8.49% |
| DFI | Attention problems - CBCL | CON-Amygdala | -0.0062 | 1.6305 | -0.0102 | 0.3213 | 0.3315 | 3.17% |
| DFI | Total problems - CBCL | CON-Amygdala | -0.0062 | 7.7332 | -0.0482 | 1.4931 | 1.5413 | 3.23% |
| DFI | ADHD - CBCL | CON-Amygdala | -0.0062 | 1.2512 | -0.0078 | 0.2848 | 0.2926 | 2.74% |
| DFI | Stress problems - CBCL | CON-Amygdala | -0.0062 | 1.6617 | -0.0104 | 0.1989 | 0.2093 | 5.21% |
| DFI | Good affections - Life Events (Parent) | CON-Amygdala | -0.0062 | 1.3393 | -0.0084 | -0.0713 | -0.0629 | 11.72% |
| DFI | Negative urgency - Impulsivity | CON-Amygdala | -0.0062 | -0.91468 | 0.0057 | 0.3235 | 0.3178 | 1.76% |
| DFI | Sensation seeking - Impulsivity | CON-Amygdala | -0.0062 | -1.4369 | 0.009 | 0.199 | 0.1901 | 4.50% |
| DFI | Positive urgency - Impulsivity | CON-Amygdala | -0.0062 | -1.713 | 0.0107 | 0.3748 | 0.3641 | 2.85% |
| DFI | BAS drive - Inhibition and Reward-seeking | CON-Amygdala | -0.0062 | -1.069 | 0.0067 | 0.2357 | 0.229 | 2.83% |
| DFI | BAS drive (modified) - Inhibition and Reward-seeking | CON-Amygdala | -0.0062 | -1.069 | 0.0067 | 0.2357 | 0.229 | 2.83% |
| DFI | Picture vocabulary - Neurocognition | CON-Hippocampus | -0.0044 | 10.355 | -0.0453 | -1.0014 | -0.9561 | 4.53% |
| DFI | Oral reading recognition - Neurocognition | CON-Hippocampus | -0.0044 | 6.6729 | -0.0292 | -0.5717 | -0.5425 | 5.11% |
| DFI | Stress problems - CBCL | CON-Hippocampus | -0.0044 | 1.8701 | -0.0082 | 0.1989 | 0.2071 | 4.11% |
| DFI | Good affections - Life Events (Parent) | CON-Hippocampus | -0.0044 | 1.7724 | -0.0078 | -0.0713 | -0.0635 | 10.88% |
| DFI | Good affections - Life Events | CON-Hippocampus | -0.0044 | 1.75 | -0.0077 | -0.0977 | -0.09 | 7.84% |
| DFI | Sensation seeking - Impulsivity | CON-Hippocampus | -0.0044 | -1.721 | 0.0075 | 0.199 | 0.1915 | 3.78% |
| DFI | Positive urgency - Impulsivity | CON-Hippocampus | -0.0044 | -1.8536 | 0.0081 | 0.3748 | 0.3667 | 2.16% |
| DFI | Reputational aggression - Adverse Peer Experiences | CON-Hippocampus | -0.0044 | -0.35286 | 0.0015 | 0.1246 | 0.123 | 1.24% |
| DFI | Reputational victimization - Adverse Peer Experiences | CON-Hippocampus | -0.0044 | -0.91794 | 0.004 | 0.2626 | 0.2585 | 1.53% |
| DFI | Picture vocabulary - Neurocognition | CON-Putamen | -0.0037 | 7.1551 | -0.0268 | -1.0014 | -0.9747 | 2.67% |
| DFI | Social problems - CBCL | CON-Putamen | -0.0037 | 1.8329 | -0.0069 | 0.1127 | 0.1195 | 6.08% |
| DFI | Thought problems - CBCL | CON-Putamen | -0.0037 | 2.1883 | -0.0082 | 0.0947 | 0.1029 | 8.64% |
| DFI | Attention problems - CBCL | CON-Putamen | -0.0037 | 3.3679 | -0.0126 | 0.3213 | 0.3339 | 3.92% |
| DFI | Aggressive problems - CBCL | CON-Putamen | -0.0037 | 2.6148 | -0.0098 | 0.4288 | 0.4386 | 2.28% |
| DFI | Total problems - CBCL | CON-Putamen | -0.0037 | 16.996 | -0.0636 | 1.4931 | 1.5567 | 4.26% |
| DFI | Depression - CBCL | CON-Putamen | -0.0037 | 1.8536 | -0.0069 | 0.0809 | 0.0879 | 8.57% |
| DFI | ADHD - CBCL | CON-Putamen | -0.0037 | 2.4197 | -0.009 | 0.2848 | 0.2938 | 3.18% |
| DFI | Stress problems - CBCL | CON-Putamen | -0.0037 | 3.8157 | -0.0143 | 0.1989 | 0.2132 | 7.17% |
| DFI | Good affections - Life Events (Parent) | CON-Putamen | -0.0037 | 2.1446 | -0.008 | -0.0713 | -0.0633 | 11.25% |
| DFI | Positive urgency - Impulsivity | CON-Putamen | -0.0037 | -1.8594 | 0.007 | 0.3748 | 0.3678 | 1.86% |
| DFI | Oral reading recognition - Neurocognition | CON-Thalamus | -0.0032 | 6.6728 | -0.0214 | -0.5717 | -0.5503 | 3.75% |
| DFI | Rule-Breaking problems - CBCL | CON-Thalamus | -0.0032 | -1.3576 | 0.0044 | 0.2984 | 0.2941 | 1.46% |
| DFI | Total problems - CBCL | CON-Thalamus | -0.0032 | -11.4 | 0.0366 | 1.4931 | 1.4565 | 2.45% |
| DFI | ADHD - CBCL | CON-Thalamus | -0.0032 | -1.7439 | 0.0056 | 0.2848 | 0.2792 | 1.97% |
| DFI | Conduct problems - CBCL | CON-Thalamus | -0.0032 | -1.4586 | 0.0047 | 0.3368 | 0.3322 | 1.39% |
| DFI | BAS drive - Inhibition and Reward-seeking | CON-Thalamus | -0.0032 | 1.9202 | -0.0062 | 0.2357 | 0.2418 | 2.62% |
| DFI | BAS drive (modified) - Inhibition and Reward-seeking | CON-Thalamus | -0.0032 | 1.9202 | -0.0062 | 0.2357 | 0.2418 | 2.62% |
| DFI | Overt aggression - Adverse Peer Experiences | CON-Thalamus | -0.0032 | -0.57958 | 0.0019 | 0.1837 | 0.1819 | 1.01% |
| DFI | Oral reading recognition - Neurocognition | CON-Ventral Diencephalon | 0.0022 | -6.1974 | -0.0139 | -0.5717 | -0.5578 | 2.43% |
| DFI | Total events - Life Events | CON-Ventral Diencephalon | 0.0022 | 1.9921 | 0.0045 | 0.3837 | 0.3792 | 1.16% |
| DFI | Bad events - Life Events | CON-Ventral Diencephalon | 0.0022 | 1.3047 | 0.0029 | 0.3135 | 0.3105 | 0.93% |
| DFI | Bad affections - Life Events | CON-Ventral Diencephalon | 0.0022 | 3.1077 | 0.007 | 0.7328 | 0.7258 | 0.95% |
| DFI | Picture vocabulary - Neurocognition | CPN-Caudate | -0.0028 | 5.7018 | -0.0157 | -1.0014 | -0.9857 | 1.57% |
| DFI | Thought problems - CBCL | CPN-Caudate | -0.0028 | 1.6556 | -0.0046 | 0.0947 | 0.0993 | 4.81% |
| DFI | Stress problems - CBCL | CPN-Caudate | -0.0028 | 2.4513 | -0.0067 | 0.1989 | 0.2057 | 3.39% |
| DFI | Mean affections - Life Events (Parent) | CPN-Caudate | -0.0028 | 0.54575 | -0.0015 | 0.0198 | 0.0213 | 7.60% |
| DFI | Good affections - Life Events | CPN-Caudate | -0.0028 | 2.1522 | -0.0059 | -0.0977 | -0.0917 | 6.06% |
| DFI | Picture vocabulary - Neurocognition | CPN-Cerebellum Cortex | -0.0044 | 6.8196 | -0.0299 | -1.0014 | -0.9715 | 2.99% |
| DFI | Oral reading recognition - Neurocognition | CPN-Cerebellum Cortex | -0.0044 | 4.8848 | -0.0214 | -0.5717 | -0.5503 | 3.75% |
| DFI | Picture vocabulary - Neurocognition | CPN-Ventral Diencephalon | -0.0028 | 7.0102 | -0.0196 | -1.0014 | -0.9818 | 1.96% |
| DFI | Good affections - Life Events | CPN-Ventral Diencephalon | -0.0028 | 2.2719 | -0.0064 | -0.0977 | -0.0913 | 6.51% |
| DFI | Total score - Prodromal Psychosis | CPN-Ventral Diencephalon | -0.0028 | -2.2164 | 0.0062 | 0.3808 | 0.3746 | 1.63% |
| DFI | Distress score - Prodromal Psychosis | CPN-Ventral Diencephalon | -0.0028 | -5.132 | 0.0144 | 1.0081 | 0.9937 | 1.42% |
| DFI | Picture vocabulary - Neurocognition | DMN-Accumbens | -0.0051 | 4.9536 | -0.0251 | -1.0014 | -0.9763 | 2.51% |
| DFI | Oral reading recognition - Neurocognition | DMN-Accumbens | -0.0051 | 3.166 | -0.0161 | -0.5717 | -0.5557 | 2.81% |
| DFI | Depression - CBCL | DMN-Accumbens | -0.0051 | 0.97421 | -0.0049 | 0.0809 | 0.0859 | 6.10% |
| DFI | Stress problems - CBCL | DMN-Accumbens | -0.0051 | 1.4081 | -0.0071 | 0.1989 | 0.2061 | 3.59% |
| DFI | Good affections - Life Events (Parent) | DMN-Accumbens | -0.0051 | 1.2846 | -0.0065 | -0.0713 | -0.0648 | 9.14% |
| DFI | Mean affections - Life Events (Parent) | DMN-Accumbens | -0.0051 | 0.39508 | -0.002 | 0.0198 | 0.0218 | 10.14% |
| DFI | Total affections - Life Events (Parent) | DMN-Accumbens | -0.0051 | 3.0108 | -0.0153 | 0.1624 | 0.1777 | 9.40% |
| DFI | Positive urgency - Impulsivity | DMN-Accumbens | -0.0051 | -1.0283 | 0.0052 | 0.3748 | 0.3696 | 1.39% |
| DFI | Picture vocabulary - Neurocognition | DMN-Amygdala | -0.0025 | 9.2739 | -0.0232 | -1.0014 | -0.9783 | 2.31% |
| DFI | Oral reading recognition - Neurocognition | DMN-Amygdala | -0.0025 | 7.1667 | -0.0179 | -0.5717 | -0.5538 | 3.13% |
| DFI | Picture vocabulary - Neurocognition | DMN-Pallidum | -0.0026 | 5.7493 | -0.0148 | -1.0014 | -0.9866 | 1.48% |
| DFI | Social problems - CBCL | DMN-Pallidum | -0.0026 | 1.3934 | -0.0036 | 0.1127 | 0.1163 | 3.19% |
| DFI | Thought problems - CBCL | DMN-Pallidum | -0.0026 | 1.8133 | -0.0047 | 0.0947 | 0.0994 | 4.94% |
| DFI | Total problems - CBCL | DMN-Pallidum | -0.0026 | 12.895 | -0.0333 | 1.4931 | 1.5264 | 2.23% |
| DFI | Depression - CBCL | DMN-Pallidum | -0.0026 | 1.5926 | -0.0041 | 0.0809 | 0.085 | 5.08% |
| DFI | Stress problems - CBCL | DMN-Pallidum | -0.0026 | 2.8546 | -0.0074 | 0.1989 | 0.2063 | 3.70% |
| DFI | Picture sequence memory - Neurocognition | DAN-Amygdala | 0.0021 | -9.5878 | -0.0198 | -1.1446 | -1.1247 | 1.73% |
| DFI | Oral reading recognition - Neurocognition | DAN-Amygdala | 0.0021 | -4.9866 | -0.0103 | -0.5717 | -0.5614 | 1.80% |
| DFI | Social problems - CBCL | DAN-Hippocampus | 0.0023 | -2.0424 | -0.0048 | 0.1127 | 0.1174 | 4.22% |
| DFI | Stress problems - CBCL | DAN-Hippocampus | 0.0023 | -2.5985 | -0.006 | 0.1989 | 0.205 | 3.04% |
| DFI | Positive urgency - Impulsivity | DAN-Hippocampus | 0.0023 | 1.9767 | 0.0046 | 0.3748 | 0.3702 | 1.23% |
| DFI | BAS fun seeking - Inhibition and Reward-seeking | DAN-Hippocampus | 0.0023 | 1.848 | 0.0043 | 0.2694 | 0.2651 | 1.60% |
| DFI | Total score - Prodromal Psychosis | DAN-Ventral Diencephalon | -0.0029 | -1.8445 | 0.0054 | 0.3808 | 0.3754 | 1.41% |
| DFI | Picture vocabulary - Neurocognition | FPN-Amygdala | -0.002 | 10.54 | -0.0212 | -1.0014 | -0.9802 | 2.12% |
| DFI | Oral reading recognition - Neurocognition | FPN-Amygdala | -0.002 | 6.0854 | -0.0123 | -0.5717 | -0.5595 | 2.14% |
| DFI | Good affections - Life Events | FPN-Amygdala | -0.002 | 2.3156 | -0.0047 | -0.0977 | -0.093 | 4.77% |
| DFI | Picture vocabulary - Neurocognition | FPN-Caudate | -0.0015 | 8.7496 | -0.013 | -1.0014 | -0.9885 | 1.29% |
| DFI | Depression - CBCL | FPN-Caudate | -0.0015 | 2.4108 | -0.0036 | 0.0809 | 0.0845 | 4.41% |
| DFI | Picture sequence memory - Neurocognition | FPN-Cerebellum Cortex | 0.0023 | -12.616 | -0.0286 | -1.1446 | -1.116 | 2.49% |
| DFI | Oral reading recognition - Neurocognition | FPN-Cerebellum Cortex | 0.0023 | -5.7835 | -0.0131 | -0.5717 | -0.5586 | 2.29% |
| DFI | Positive urgency - Impulsivity | FPN-Cerebellum Cortex | 0.0023 | 2.6042 | 0.0059 | 0.3748 | 0.3689 | 1.57% |
| DFI | Lack of planning - Impulsivity | FPN-Thalamus | 0.0025 | 1.5419 | 0.0039 | 0.3009 | 0.297 | 1.28% |
| DFI | Lack of perseverance - Impulsivity | FPN-Thalamus | 0.0025 | 1.4024 | 0.0035 | 0.2504 | 0.2469 | 1.40% |
| DFI | Oral reading recognition - Neurocognition | RTN-Accumbens | -0.0026 | 4.3385 | -0.0112 | -0.5717 | -0.5605 | 1.96% |
| DFI | Picture vocabulary - Neurocognition | RTN-Ventral Diencephalon | -0.0032 | 7.447 | -0.0236 | -1.0014 | -0.9778 | 2.36% |
| DFI | Thought problems - CBCL | RTN-Ventral Diencephalon | -0.0032 | 1.4252 | -0.0045 | 0.0947 | 0.0992 | 4.77% |
| DFI | Attention problems - CBCL | RTN-Ventral Diencephalon | -0.0032 | 1.6234 | -0.0051 | 0.3213 | 0.3265 | 1.60% |
| DFI | Stress problems - CBCL | RTN-Ventral Diencephalon | -0.0032 | 2.2245 | -0.0071 | 0.1989 | 0.206 | 3.55% |
| DFI | Good affections - Life Events (Parent) | RTN-Ventral Diencephalon | -0.0032 | 1.4635 | -0.0046 | -0.0713 | -0.0666 | 6.51% |
| DFI | Sensation seeking - Impulsivity | RTN-Ventral Diencephalon | -0.0032 | -1.3107 | 0.0042 | 0.199 | 0.1949 | 2.09% |
| DFI | Positive urgency - Impulsivity | RTN-Ventral Diencephalon | -0.0032 | -1.5243 | 0.0048 | 0.3748 | 0.3699 | 1.29% |
| DFI | Picture vocabulary - Neurocognition | SN-Ventral Diencephalon | 0.003 | -7.0883 | -0.0213 | -1.0014 | -0.9802 | 2.12% |
| DFI | Oral reading recognition - Neurocognition | SN-Ventral Diencephalon | 0.003 | -6.0914 | -0.0183 | -0.5717 | -0.5534 | 3.20% |
| DFI | Total events - Life Events | SN-Ventral Diencephalon | 0.003 | 2.5617 | 0.0077 | 0.3837 | 0.376 | 2% |
| DFI | Bad events - Life Events | SN-Ventral Diencephalon | 0.003 | 2.0081 | 0.006 | 0.3135 | 0.3074 | 1.92% |
| DFI | Bad affections - Life Events | SN-Ventral Diencephalon | 0.003 | 4.7266 | 0.0142 | 0.7328 | 0.7186 | 1.94% |
| DFI | Positive urgency - Impulsivity | SN-Ventral Diencephalon | 0.003 | 2.0176 | 0.0061 | 0.3748 | 0.3687 | 1.62% |
| DFI | Picture vocabulary - Neurocognition | SHN-Accumbens | -0.0035 | 9.418 | -0.0326 | -1.0014 | -0.9688 | 3.26% |
| DFI | Oral reading recognition - Neurocognition | SHN-Accumbens | -0.0035 | 4.4114 | -0.0153 | -0.5717 | -0.5564 | 2.67% |
| DFI | Good affections - Life Events | SHN-Accumbens | -0.0035 | 1.9982 | -0.0069 | -0.0977 | -0.0907 | 7.09% |
| DFI | Positive urgency - Impulsivity | SHN-Accumbens | -0.0035 | -1.9988 | 0.0069 | 0.3748 | 0.3678 | 1.85% |
| DFI | Picture vocabulary - Neurocognition | SHN-Caudate | -0.0044 | 9.7623 | -0.0427 | -1.0014 | -0.9587 | 4.26% |
| DFI | Social problems - CBCL | SHN-Caudate | -0.0044 | 1.3683 | -0.006 | 0.1127 | 0.1187 | 5.31% |
| DFI | Thought problems - CBCL | SHN-Caudate | -0.0044 | 2.4075 | -0.0105 | 0.0947 | 0.1052 | 11.11% |
| DFI | Attention problems - CBCL | SHN-Caudate | -0.0044 | 2.843 | -0.0124 | 0.3213 | 0.3338 | 3.87% |
| DFI | Total problems - CBCL | SHN-Caudate | -0.0044 | 14.679 | -0.0642 | 1.4931 | 1.5573 | 4.30% |
| DFI | Depression - CBCL | SHN-Caudate | -0.0044 | 1.8349 | -0.008 | 0.0809 | 0.0889 | 9.92% |
| DFI | ADHD - CBCL | SHN-Caudate | -0.0044 | 1.865 | -0.0082 | 0.2848 | 0.293 | 2.86% |
| DFI | Stress problems - CBCL | SHN-Caudate | -0.0044 | 3.5798 | -0.0157 | 0.1989 | 0.2146 | 7.87% |
| DFI | Good events - Life Events (Parent) | SHN-Caudate | -0.0044 | 0.84595 | -0.0037 | -0.0473 | -0.0436 | 7.82% |
| DFI | Good affections - Life Events (Parent) | SHN-Caudate | -0.0044 | 2.2712 | -0.0099 | -0.0713 | -0.0614 | 13.93% |
| DFI | Total affections - Life Events (Parent) | SHN-Caudate | -0.0044 | 3.117 | -0.0136 | 0.1624 | 0.1761 | 8.39% |
| DFI | Bad events - Life Events | SHN-Caudate | -0.0044 | -1.2609 | 0.0055 | 0.3135 | 0.3079 | 1.76% |
| DFI | Good affections - Life Events | SHN-Caudate | -0.0044 | 1.8327 | -0.008 | -0.0977 | -0.0896 | 8.21% |
| DFI | Sensation seeking - Impulsivity | SHN-Caudate | -0.0044 | -1.9297 | 0.0084 | 0.199 | 0.1906 | 4.24% |
| DFI | Positive urgency - Impulsivity | SHN-Caudate | -0.0044 | -2.2787 | 0.01 | 0.3748 | 0.3648 | 2.66% |
| DFI | Picture vocabulary - Neurocognition | SHN-Cerebellum Cortex | -0.0032 | 5.9558 | -0.019 | -1.0014 | -0.9824 | 1.90% |
| DFI | Social problems - CBCL | SHN-Cerebellum Cortex | -0.0032 | 1.4897 | -0.0048 | 0.1127 | 0.1174 | 4.22% |
| DFI | Thought problems - CBCL | SHN-Cerebellum Cortex | -0.0032 | 1.6125 | -0.0051 | 0.0947 | 0.0999 | 5.43% |
| DFI | Attention problems - CBCL | SHN-Cerebellum Cortex | -0.0032 | 2.1841 | -0.007 | 0.3213 | 0.3283 | 2.17% |
| DFI | Total problems - CBCL | SHN-Cerebellum Cortex | -0.0032 | 11.614 | -0.0371 | 1.4931 | 1.5302 | 2.48% |
| DFI | Depression - CBCL | SHN-Cerebellum Cortex | -0.0032 | 1.3697 | -0.0044 | 0.0809 | 0.0853 | 5.40% |
| DFI | Stress problems - CBCL | SHN-Cerebellum Cortex | -0.0032 | 2.9341 | -0.0094 | 0.1989 | 0.2083 | 4.71% |
| DFI | Good events - Life Events (Parent) | SHN-Cerebellum Cortex | -0.0032 | 0.83071 | -0.0027 | -0.0473 | -0.0447 | 5.60% |
| DFI | Good affections - Life Events (Parent) | SHN-Cerebellum Cortex | -0.0032 | 1.9685 | -0.0063 | -0.0713 | -0.065 | 8.81% |
| DFI | Bad events - Life Events | SHN-Cerebellum Cortex | -0.0032 | -1.3845 | 0.0044 | 0.3135 | 0.309 | 1.41% |
| DFI | Positive urgency - Impulsivity | SHN-Cerebellum Cortex | -0.0032 | -2.0256 | 0.0065 | 0.3748 | 0.3683 | 1.72% |
| DFI | Picture vocabulary - Neurocognition | SHN-Pallidum | -0.0043 | 4.767 | -0.0205 | -1.0014 | -0.981 | 2.04% |
| DFI | Thought problems - CBCL | SHN-Pallidum | -0.0043 | 1.6232 | -0.007 | 0.0947 | 0.1017 | 7.35% |
| DFI | Attention problems - CBCL | SHN-Pallidum | -0.0043 | 2.2722 | -0.0098 | 0.3213 | 0.3311 | 3.03% |
| DFI | Total problems - CBCL | SHN-Pallidum | -0.0043 | 11.242 | -0.0482 | 1.4931 | 1.5414 | 3.23% |
| DFI | Depression - CBCL | SHN-Pallidum | -0.0043 | 1.2986 | -0.0056 | 0.0809 | 0.0865 | 6.89% |
| DFI | ADHD - CBCL | SHN-Pallidum | -0.0043 | 1.6772 | -0.0072 | 0.2848 | 0.292 | 2.53% |
| DFI | Stress problems - CBCL | SHN-Pallidum | -0.0043 | 2.5814 | -0.0111 | 0.1989 | 0.21 | 5.57% |
| DFI | Good affections - Life Events (Parent) | SHN-Pallidum | -0.0043 | 1.5737 | -0.0068 | -0.0713 | -0.0645 | 9.47% |
| DFI | Distress score - Prodromal Psychosis | SHN-Pallidum | -0.0043 | -4.4637 | 0.0192 | 1.0081 | 0.9889 | 1.90% |
| DFI | Positive urgency - Impulsivity | SHN-Pallidum | -0.0043 | -1.7791 | 0.0076 | 0.3748 | 0.3671 | 2.04% |
| DFI | Picture vocabulary - Neurocognition | SHN-Putamen | -0.006 | 9.1283 | -0.0543 | -1.0014 | -0.9471 | 5.43% |
| DFI | Oral reading recognition - Neurocognition | SHN-Putamen | -0.006 | 5.5398 | -0.033 | -0.5717 | -0.5387 | 5.77% |
| DFI | Good affections - Life Events (Parent) | SHN-Putamen | -0.006 | 1.6916 | -0.0101 | -0.0713 | -0.0612 | 14.13% |
| DFI | Good affections - Life Events | SHN-Putamen | -0.006 | 2.0097 | -0.012 | -0.0977 | -0.0857 | 12.25% |
| DFI | Negative urgency - Impulsivity | SHN-Putamen | -0.006 | -1.3492 | 0.008 | 0.3235 | 0.3155 | 2.48% |
| DFI | Sensation seeking - Impulsivity | SHN-Putamen | -0.006 | -1.267 | 0.0075 | 0.199 | 0.1915 | 3.79% |
| DFI | Positive urgency - Impulsivity | SHN-Putamen | -0.006 | -2.4068 | 0.0143 | 0.3748 | 0.3604 | 3.82% |
| DFI | Mean affections - Life Events (Parent) | SMN-Amygdala | -0.0026 | 0.55089 | -0.0014 | 0.0198 | 0.0212 | 7.18% |
| DFI | Total score - Prodromal Psychosis | SMN-Amygdala | -0.0026 | -1.5787 | 0.0041 | 0.3808 | 0.3767 | 1.07% |
| DFI | Picture vocabulary - Neurocognition | SMN-Hippocampus | -0.003 | 6.4764 | -0.0195 | -1.0014 | -0.982 | 1.94% |
| DFI | Stress problems - CBCL | SMN-Hippocampus | -0.003 | 2.2488 | -0.0068 | 0.1989 | 0.2057 | 3.40% |
| DFI | Total score - Prodromal Psychosis | SMN-Hippocampus | -0.003 | -1.8976 | 0.0057 | 0.3808 | 0.3751 | 1.50% |
| DFI | Distress score - Prodromal Psychosis | SMN-Hippocampus | -0.003 | -4.676 | 0.0141 | 1.0081 | 0.994 | 1.39% |
| DFI | Picture vocabulary - Neurocognition | VAN-Caudate | -0.0024 | 7.1417 | -0.0169 | -1.0014 | -0.9845 | 1.69% |
| DFI | Oral reading recognition - Neurocognition | VAN-Caudate | -0.0024 | 6.6591 | -0.0158 | -0.5717 | -0.5559 | 2.76% |
| DFI | Good affections - Life Events (Parent) | VAN-Caudate | -0.0024 | 2.6253 | -0.0062 | -0.0713 | -0.0651 | 8.72% |
| DFI | Good affections - Life Events | VAN-Caudate | -0.0024 | 2.3842 | -0.0056 | -0.0977 | -0.092 | 5.78% |
| DFI | Positive urgency - Impulsivity | VAN-Caudate | -0.0024 | -1.9422 | 0.0046 | 0.3748 | 0.3702 | 1.23% |
| DFI | Picture vocabulary - Neurocognition | VN-Hippocampus | -0.0046 | 6.4294 | -0.0297 | -1.0014 | -0.9718 | 2.96% |
| DFI | Oral reading recognition - Neurocognition | VN-Hippocampus | -0.0046 | 2.971 | -0.0137 | -0.5717 | -0.558 | 2.40% |
| DFI | Thought problems - CBCL | VN-Hippocampus | -0.0046 | 0.98178 | -0.0045 | 0.0947 | 0.0993 | 4.78% |
| DFI | Good affections - Life Events (Parent) | VN-Hippocampus | -0.0046 | 1.2394 | -0.0057 | -0.0713 | -0.0656 | 8.03% |
| DFI | Good affections - Life Events | VN-Hippocampus | -0.0046 | 1.5294 | -0.0071 | -0.0977 | -0.0906 | 7.23% |
| DFI | Positive urgency - Impulsivity | VN-Hippocampus | -0.0046 | -1.6782 | 0.0077 | 0.3748 | 0.367 | 2.07% |
| DFI | Picture vocabulary - Neurocognition | VN-Pallidum | -0.0024 | 10.54 | -0.0249 | -1.0014 | -0.9766 | 2.48% |
| DFI | Oral reading recognition - Neurocognition | VN-Pallidum | -0.0024 | 5.3099 | -0.0125 | -0.5717 | -0.5592 | 2.19% |
| DFI | Thought problems - CBCL | VN-Pallidum | -0.0024 | 1.9772 | -0.0047 | 0.0947 | 0.0994 | 4.93% |
| DFI | Stress problems - CBCL | VN-Pallidum | -0.0024 | 3.4213 | -0.0081 | 0.1989 | 0.207 | 4.06% |
| DFI | Picture vocabulary - Neurocognition | CON-Brain Stem | -0.0043 | 7.586 | -0.0323 | -1.0014 | -0.9692 | 3.22% |
| DFI | Social problems - CBCL | CON-Brain Stem | -0.0043 | 1.1617 | -0.0049 | 0.1127 | 0.1176 | 4.39% |
| DFI | Thought problems - CBCL | CON-Brain Stem | -0.0043 | 2.0071 | -0.0085 | 0.0947 | 0.1033 | 9.01% |
| DFI | Attention problems - CBCL | CON-Brain Stem | -0.0043 | 2.1786 | -0.0093 | 0.3213 | 0.3306 | 2.88% |
| DFI | Total problems - CBCL | CON-Brain Stem | -0.0043 | 12.348 | -0.0525 | 1.4931 | 1.5456 | 3.52% |
| DFI | Depression - CBCL | CON-Brain Stem | -0.0043 | 1.39 | -0.0059 | 0.0809 | 0.0868 | 7.31% |
| DFI | Sluggish cognitive tempo - CBCL | CON-Brain Stem | -0.0043 | 0.60308 | -0.0026 | 0.0275 | 0.03 | 9.34% |
| DFI | Stress problems - CBCL | CON-Brain Stem | -0.0043 | 2.6935 | -0.0115 | 0.1989 | 0.2104 | 5.76% |
| DFI | BAS drive - Inhibition and Reward-seeking | CON-Brain Stem | -0.0043 | -1.4621 | 0.0062 | 0.2357 | 0.2294 | 2.64% |
| DFI | BAS drive (modified) - Inhibition and Reward-seeking | CON-Brain Stem | -0.0043 | -1.4621 | 0.0062 | 0.2357 | 0.2294 | 2.64% |
| DFI | Social problems - CBCL | DAN-Brain Stem | -0.0042 | 1.1081 | -0.0046 | 0.1127 | 0.1173 | 4.12% |
| DFI | Thought problems - CBCL | DAN-Brain Stem | -0.0042 | 1.0597 | -0.0044 | 0.0947 | 0.0992 | 4.69% |
| DFI | Total problems - CBCL | DAN-Brain Stem | -0.0042 | 9.0234 | -0.0378 | 1.4931 | 1.5309 | 2.53% |
| DFI | Depression - CBCL | DAN-Brain Stem | -0.0042 | 1.2174 | -0.0051 | 0.0809 | 0.086 | 6.30% |
| DFI | Stress problems - CBCL | DAN-Brain Stem | -0.0042 | 1.8855 | -0.0079 | 0.1989 | 0.2068 | 3.97% |
| DFI | Good events - Life Events (Parent) | DAN-Brain Stem | -0.0042 | 0.72657 | -0.003 | -0.0473 | -0.0443 | 6.44% |
| DFI | Good affections - Life Events (Parent) | DAN-Brain Stem | -0.0042 | 1.4675 | -0.0061 | -0.0713 | -0.0651 | 8.63% |
| DFI | Picture vocabulary - Neurocognition | RTN-Brain Stem | -0.0051 | 4.9485 | -0.0253 | -1.0014 | -0.9762 | 2.52% |
| DFI | Oral reading recognition - Neurocognition | RTN-Brain Stem | -0.0051 | 2.8328 | -0.0145 | -0.5717 | -0.5573 | 2.53% |
| DFI | Positive urgency - Impulsivity | RTN-Brain Stem | -0.0051 | -1.1932 | 0.0061 | 0.3748 | 0.3687 | 1.62% |
| DFI | Distress score - Prodromal Psychosis | SHN-Brain Stem | -0.0043 | -3.9799 | 0.0173 | 1.0081 | 0.9908 | 1.71% |
| DFI | Positive urgency - Impulsivity | SHN-Brain Stem | -0.0043 | -1.5003 | 0.0065 | 0.3748 | 0.3683 | 1.74% |
| DFI | BAS drive - Inhibition and Reward-seeking | SHN-Brain Stem | -0.0043 | -1.3548 | 0.0059 | 0.2357 | 0.2298 | 2.50% |
| DFI | BAS drive (modified) - Inhibition and Reward-seeking | SHN-Brain Stem | -0.0043 | -1.3548 | 0.0059 | 0.2357 | 0.2298 | 2.50% |

**Supplementary Table 6 |** The significant results of longitudinal analyses of peer environments. Significant associations were observed between PFI/DFI at 2-year follow-up and behavioral variables at 3-year follow-up (*p*_fdr_ < 0.05). PFI had at 2-year follow-up had 19 longitudinal associations with behavioral variables at 3-year follow-up, while DFI had at 2-year follow-up had 22 longitudinal associations with behavioral variables at 3-year follow-up.

| **Peer** | **Variables** | **Standardized**  ***β*** | ***β (*CI lower)** | ***β (*CI upper)** | **SE** | ***p*_fdr_** |
| --- | --- | --- | --- | --- | --- | --- |
| PFI | Anxious/Depressed problems - CBCL | -0.033 | -0.052 | -0.014 | 0.01 | 2.40E-03 |
| PFI | Withdrawn/Depressed problems - CBCL | -0.061 | -0.081 | -0.041 | 0.01 | 1.90E-07 |
| PFI | Somatic complaint problems - CBCL | -0.044 | -0.065 | -0.023 | 0.011 | 3.70E-04 |
| PFI | Thought problems - CBCL | -0.029 | -0.049 | -0.01 | 0.01 | 8.70E-03 |
| PFI | Attention problems - CBCL | -0.028 | -0.045 | -0.011 | 0.009 | 4.50E-03 |
| PFI | Rule-Breaking problems - CBCL | -0.03 | -0.05 | -0.01 | 0.01 | 8.20E-03 |
| PFI | Internalizing problems - CBCL | -0.05 | -0.069 | -0.031 | 0.01 | 4.40E-06 |
| PFI | Total problems - CBCL | -0.036 | -0.054 | -0.019 | 0.009 | 3.70E-04 |
| PFI | Depression - CBCL | -0.059 | -0.08 | -0.039 | 0.01 | 2.90E-07 |
| PFI | Anxiety - CBCL | -0.038 | -0.058 | -0.019 | 0.01 | 7.20E-04 |
| PFI | Somatic - CBCL | -0.039 | -0.061 | -0.017 | 0.011 | 1.80E-03 |
| PFI | ADHD - CBCL | -0.025 | -0.042 | -0.008 | 0.009 | 1.20E-02 |
| PFI | Conduct problems - CBCL | -0.023 | -0.043 | -0.004 | 0.01 | 4.00E-02 |
| PFI | Sluggish cognitive tempo - CBCL | -0.039 | -0.06 | -0.019 | 0.01 | 9.90E-04 |
| PFI | Stress problems - CBCL | -0.033 | -0.052 | -0.015 | 0.01 | 1.90E-03 |
| PFI | Total events - Life Events (Parent) | -0.033 | -0.057 | -0.009 | 0.012 | 1.70E-02 |
| PFI | Total affections - Life Events (Parent) | -0.028 | -0.051 | -0.005 | 0.012 | 3.70E-02 |
| PFI | Total score - Prodromal Psychosis | -0.044 | -0.067 | -0.022 | 0.012 | 7.20E-04 |
| PFI | Distress score - Prodromal Psychosis | -0.043 | -0.066 | -0.02 | 0.012 | 1.30E-03 |
| DFI | Social problems - CBCL | 0.026 | 0.007 | 0.045 | 0.01 | 1.50E-02 |
| DFI | Attention problems - CBCL | 0.021 | 0.004 | 0.039 | 0.009 | 2.70E-02 |
| DFI | Rule-Breaking problems - CBCL | 0.064 | 0.044 | 0.084 | 0.01 | 5.70E-09 |
| DFI | Aggressive problems - CBCL | 0.025 | 0.007 | 0.044 | 0.009 | 1.40E-02 |
| DFI | Externalizing problems - CBCL | 0.036 | 0.018 | 0.054 | 0.009 | 5.00E-04 |
| DFI | Total problems - CBCL | 0.023 | 0.006 | 0.04 | 0.009 | 2.00E-02 |
| DFI | Somatic - CBCL | 0.03 | 0.008 | 0.052 | 0.011 | 1.70E-02 |
| DFI | ADHD - CBCL | 0.032 | 0.015 | 0.049 | 0.009 | 9.10E-04 |
| DFI | Oppositional defiant problems - CBCL | 0.024 | 0.005 | 0.042 | 0.01 | 2.60E-02 |
| DFI | Conduct problems - CBCL | 0.058 | 0.038 | 0.077 | 0.01 | 4.50E-08 |
| DFI | Bad events - Life Events (Parent) | 0.036 | 0.013 | 0.059 | 0.012 | 5.90E-03 |
| DFI | Bad affections - Life Events (Parent) | 0.03 | 0.008 | 0.052 | 0.011 | 1.70E-02 |
| DFI | Bad events - Life Events | 0.048 | 0.025 | 0.071 | 0.012 | 2.40E-04 |
| DFI | Bad affections - Life Events | -0.038 | -0.06 | -0.015 | 0.012 | 4.10E-03 |
| DFI | Mean affections - Life Events | 0.043 | 0.021 | 0.066 | 0.012 | 7.50E-04 |
| DFI | Total score - Prodromal Psychosis | 0.042 | 0.019 | 0.064 | 0.012 | 1.20E-03 |
| DFI | Distress score - Prodromal Psychosis | 0.037 | 0.013 | 0.06 | 0.012 | 5.90E-03 |
| DFI | Reputational aggression - Adverse Peer Experiences | 0.074 | 0.049 | 0.098 | 0.012 | 2.10E-08 |
| DFI | Reputational victimization - Adverse Peer Experiences | 0.067 | 0.044 | 0.089 | 0.012 | 4.10E-08 |
| DFI | Overt aggression - Adverse Peer Experiences | 0.092 | 0.069 | 0.116 | 0.012 | 6.50E-13 |
| DFI | Overt victimization - Adverse Peer Experiences | 0.093 | 0.069 | 0.116 | 0.012 | 4.60E-13 |
| DFI | Relational aggression - Adverse Peer Experiences | 0.073 | 0.05 | 0.097 | 0.012 | 1.30E-08 |

**Supplementary Table 7 |** Partial correlations between peer environments and social factors, controlling for covariates in supplementary analyses (2-year follow-up). The first-order partial correlations were calculated between peer environments (PFI/DFI) and each socioeconomic factor (family income, parental education, the number of male friends, the number of female friends, family conflict, neighborhood safety, school environment, school involvement, school disengagement), while controlling for the remaining socioeconomic variables in the model. All factors showed significant associations with both PFI and DFI, except for parental education, which was not significantly correlated with either index. These variables were subsequently included as covariates in supplementary analyses to account for potential confounding effects. * indicates that these variables were already accounted for in the primary analyses.

| Peer Environments | Social Factors | Statistic | Partial Correlation | *p* value |
| --- | --- | --- | --- | --- |
| PFI | Family income* | 11.18 | 0.127 | 8.20E-29 |
| PFI | Parental education* | 0.56 | 0.006 | 5.75E-01 |
| PFI | The number of male friends | 9.23 | 0.105 | 3.51E-20 |
| PFI | The number of female friends | 6.5 | 0.074 | 8.46E-11 |
| PFI | Family conflict | 3.78 | 0.043 | 1.55E-04 |
| PFI | Neighborhood safety | 3.52 | 0.04 | 4.34E-04 |
| PFI | School environment | 5.58 | 0.063 | 2.54E-08 |
| PFI | School involvement | 10.53 | 0.119 | 9.12E-26 |
| PFI | School disengagement | -4.56 | -0.052 | 5.17E-06 |
| DFI | Family income* | -18.38 | -0.205 | 6.83E-74 |
| DFI | Parental education* | -0.8 | -0.009 | 4.21E-01 |
| DFI | The number of male friends | 12.29 | 0.139 | 2.17E-34 |
| DFI | The number of female friends | 5.6 | 0.064 | 2.25E-08 |
| DFI | Family conflict | 3.22 | 0.037 | 1.28E-03 |
| DFI | Neighborhood safety | -3.8 | -0.043 | 1.46E-04 |
| DFI | School environment | -4.03 | -0.046 | 5.63E-05 |
| DFI | School involvement | -4.01 | -0.046 | 6.23E-05 |
| DFI | School disengagement | 8.85 | 0.1 | 1.10E-18 |

**Supplementary Figures**

**Supplementary Fig. 1** **|** Sample inclusion criteria. For each analysis, we used different criteria to maximize the sample size. (a) Sample inclusion criteria for association analyses. (b) Sample inclusion criteria for mediation analyses. (c) Sample inclusion criteria for longitudinal analyses. Abbreviations: 2YFU = 2-year follow-up. 3YFU = 3-year follow-up.


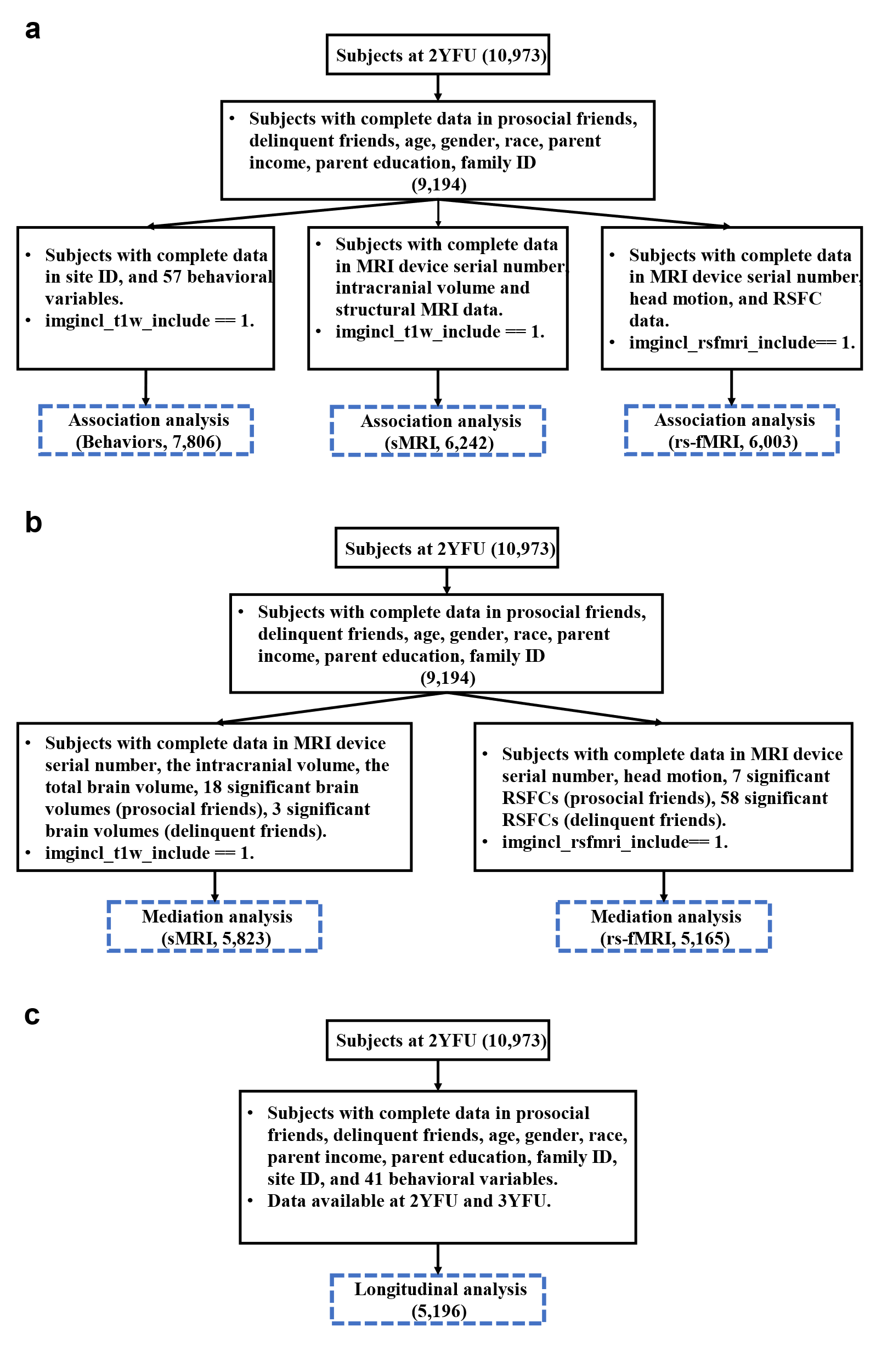


**Supplementary Fig. 2 | Significant results between peer environments and cortical thickness.** Significant results between DFI and cortical thickness in 3 regions, including the bilateral lateral occipital and the right middle temporal (*p*_fdr_ < 0.05). See detailed results in Supplementary Table 4.


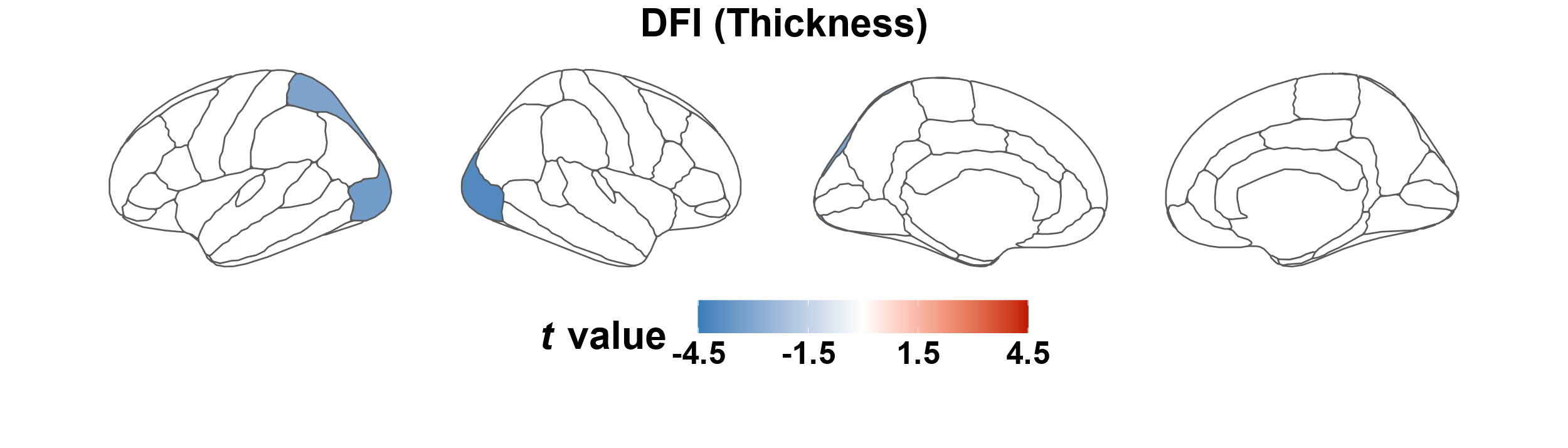


**Supplementary Fig. 3 |** Spatial correlations between the *t*-maps of PFI associations and neurotransmitter density. (a-c) No significant results between the *t*-maps from PFI (volume, area, thickness) and neurotransmitter density maps.


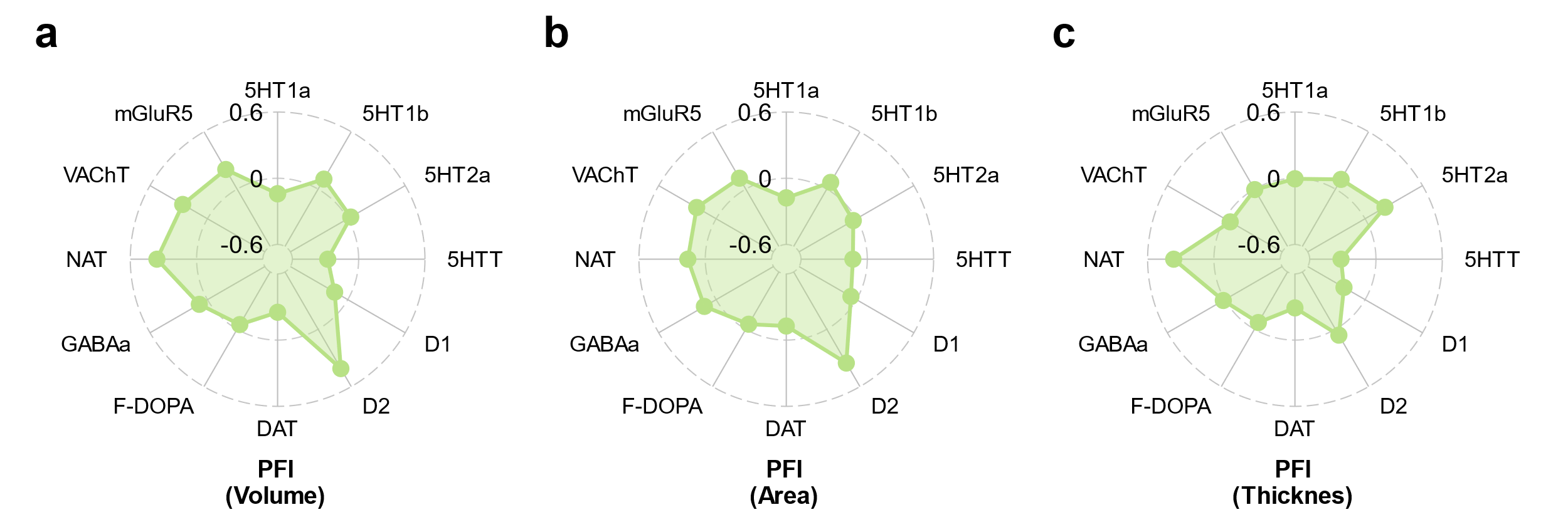


**Supplementary Fig. 4 | The cortico-subcortical RSFCs mediated the relationships between the DFI and behaviors.** Each triangle/circle represents one peer-behavior relationship. (a) The mediation analysis of DFI. X = DFI, Y = 57 behavioral-cognitive measurements, M = brain features (46 cortico-subcortical RSFCs that were significantly associated with DFI). (b) The relationships between DFI and behaviors were mediated by cortico-subcortical RSFCs. A total of 199 significant results were identified, with the majority of significant mediators involving frontal-limbic and frontostriatal RSFCs (*p*_fdr_ < 0.05, FDR correction times = 46 cortico-subcortical RSFCs × 57 behavioral-cognitive measurements). Additionally, the within DMN-ventral diencephalon connectivity mediated the relationship between PFI and parent-reported total life events (*p*_fdr_ < 0.05, FDR correction times = 2 cortico-subcortical RSFCs × 57 behavioral-cognitive measurements). See detailed results in Supplementary Table 5. Abbreviation: AN = auditory network; VN = visual network; SHN = sensorimotor hand network; SMN = sensorimotor mouth network; CON = cingulo-opercular network; CPN = cingulo-parietal network; DAN = dorsal attention network; DMN = default mode network; FPN = fronto-parietal network; RTN = retrosplenial temporal network; SN = salience network; VAN = ventral attention network.


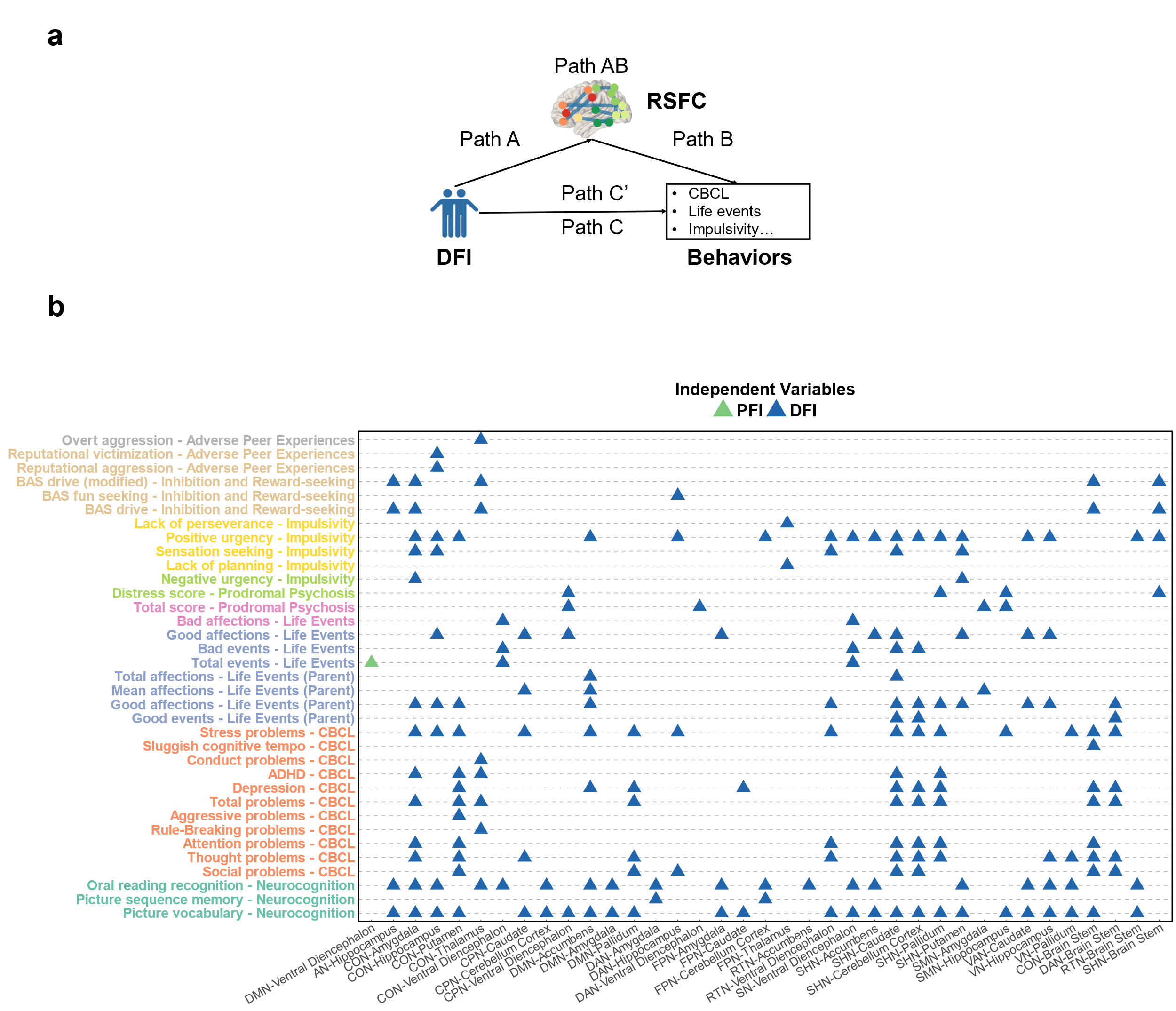
 **Supplementary Fig. 5 | Associations between peer environments and psychopathology-relevant behaviors (supplementary analyses).** Additional confounders were included to further test the robustness of our results: family conflict (ce_y_fes), school risk and protective factors (ce_y_srpf), neighborhood safety (ce_y_nsc), school grades from last year (ce_p_sag), the number of male and female friends (mh_y_or). Similar to the primary analyses, PFI predominantly exhibited negative associations with psychopathology-relevant behaviors, whereas DFI had positive associations with those variables. PFI had 33 (originally 47) significant associations with behavioral variables, while DFI had 35 (originally 40) significant associations with behavioral variables. In detail, A larger PFI correlated with fewer behavioral problems (14 out of 20 items), parent-reported life events (3 out of 7 items), prodromal psychosis scores, impulsivity (3 out of 5 items), and adverse peer experiences (2 out of 5 items). A larger DFI was associated with more behavioral problems (12 out of 20 items), life events (4 out of 6 items), prodromal psychosis scores, impulsivity, and adverse peer experiences.


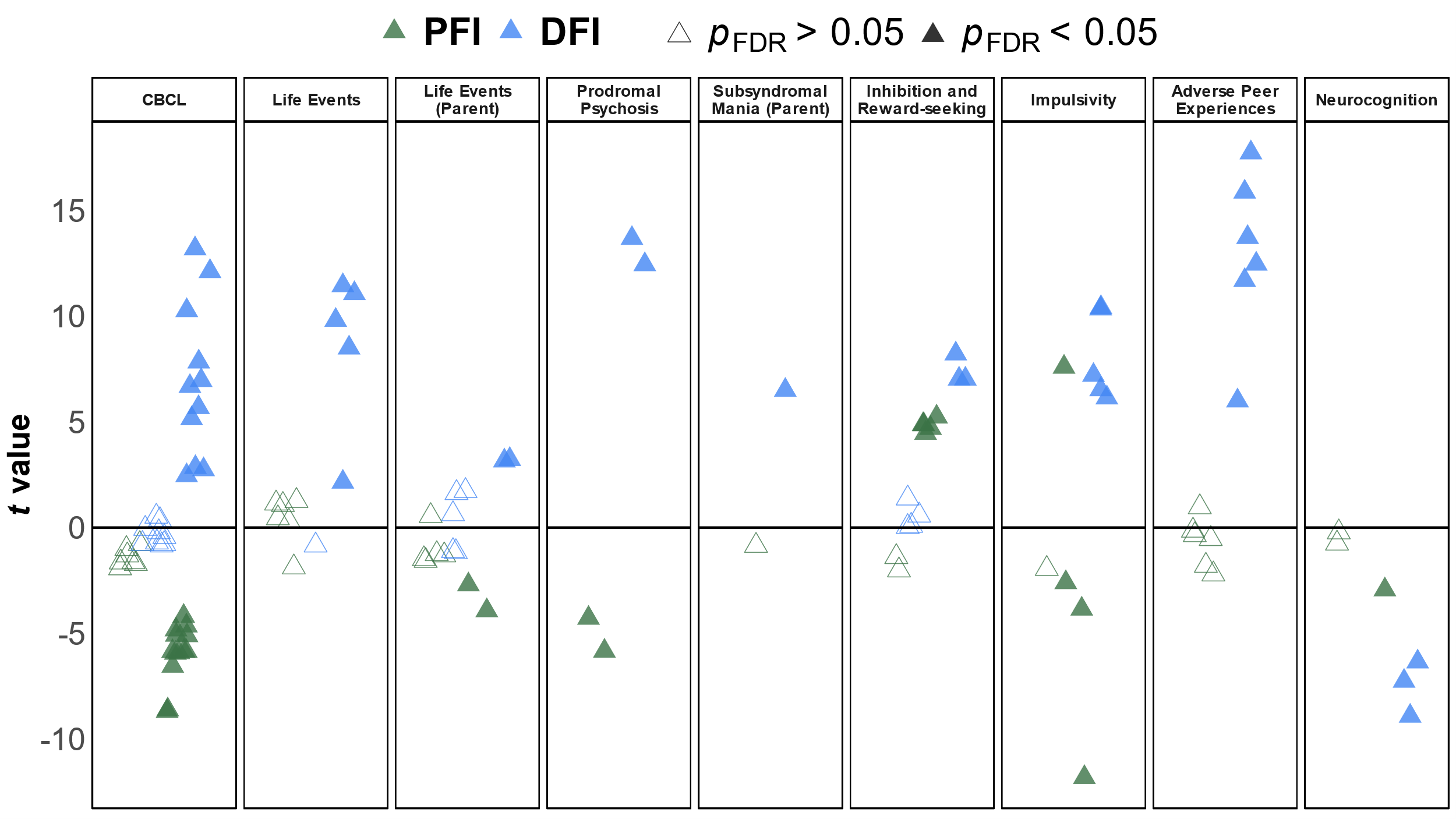


**Supplementary Fig. 6** **| Associations between peer environments and regional brain volumes (supplementary analyses).** The associations between PFI, the total volumes, and total areas are still significant (total volume: *t* = 3.48, R2 s*p* = 0.0012, *p* = 4.9 × 10^-4^; total area: *t* = 2.67, R2 s*p* = 0.0006, *p* = 0.0076). (a) PFI had positive associations with 2 volumes (originally 14), including the left inferior parietal (*t* = 3.33, R2 s*p* = 0.0017, *p*_fdr_ = 0.039), the left precentral (*t* = 3.26, R2 s*p* = 0.0016, *p*_fdr_ = 0.039). In addition, PFI had positive associations with 2 subcortical volumes (originally 4), including the left putamen (*t* = 3.35, R2 s*p* = 0.0017, *p*_fdr_ = 0.007), and the right pallidum (*t* = 3.35, R2 s*p* = 0.0017, *p*_fdr_ = 0.006). (b) The association between DFI and the lateral occipital volume (*t* = -3.45, R2 s*p* = 0.0019, *p*_fdr_ = 0.039; originally 3). (c) The association between DFI and the lateral occipital thickness (*t* = -3.43, R2 s*p* = 0.0016, *p*_fdr_ = 0.041; originally 3).


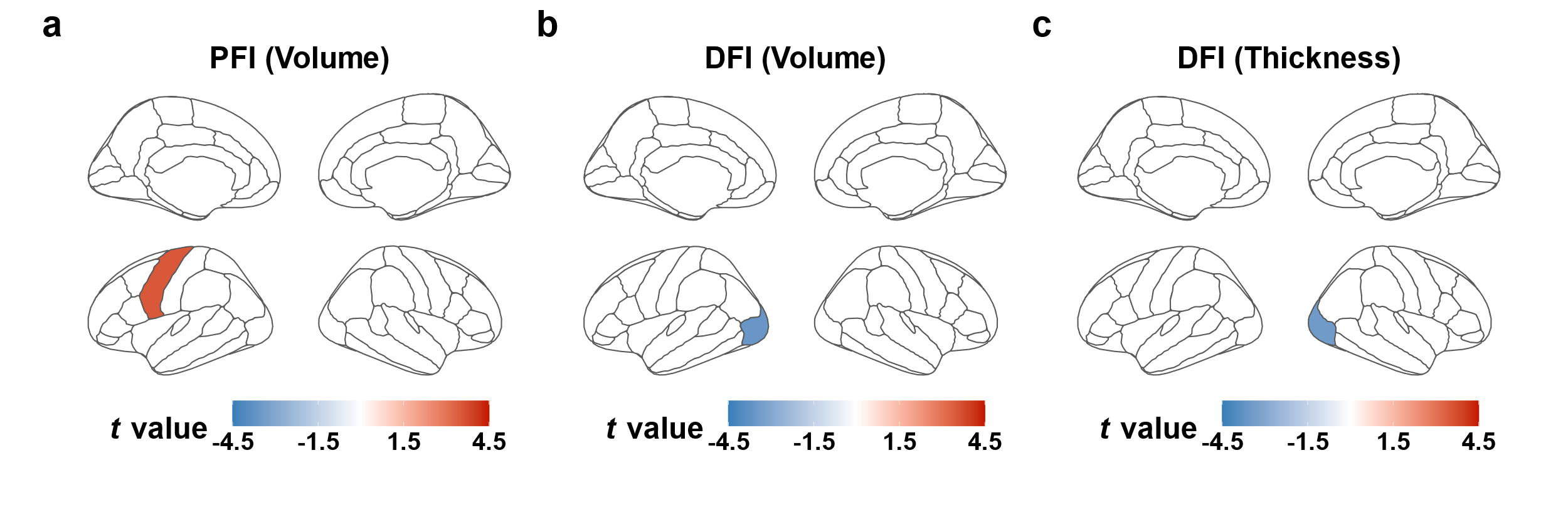


**Supplementary Fig. 7** **| Associations between peer environments and RSFCs (supplementary analyses).** Compared with the primary analyses, the majority of findings within DFI remained consistent. DFI had associations with 6 RSFCs (originally 12), such as within DMN connectivity (*t* = -3.57, R2 s*p* = 0.0019, *p*_fdr_ = 0.009). (c) DFI had associations with 25 cortico-subcortical RSFCs (originally 36), such as CON-amygdala connectivity (*t* = -3.73, R2 s*p* = 0.0023, *p*_fdr_ = 0.003). Abbreviation: auditory network (AN), visual network (VN), sensorimotor hand network (SHN), sensorimotor mouth network (AN), cingulo-opercular network (CON), cingulo-parietal network (CPN), dorsal attention network (DAN), default mode network (DMN), fronto-parietal network (FPN), retrosplenial temporal network (RTN), salience network (SN), ventral attention network (VAN); cerebellum cortex (Crcx), thalamus (Tha), hippocampus (Hip), amygdala (Amg), putamen (Pt), pallidum (Pl), caudate (Cde), nucleus accumbens (NAc), ventral diencephalon (Vtcd) and brain-stem (BS).


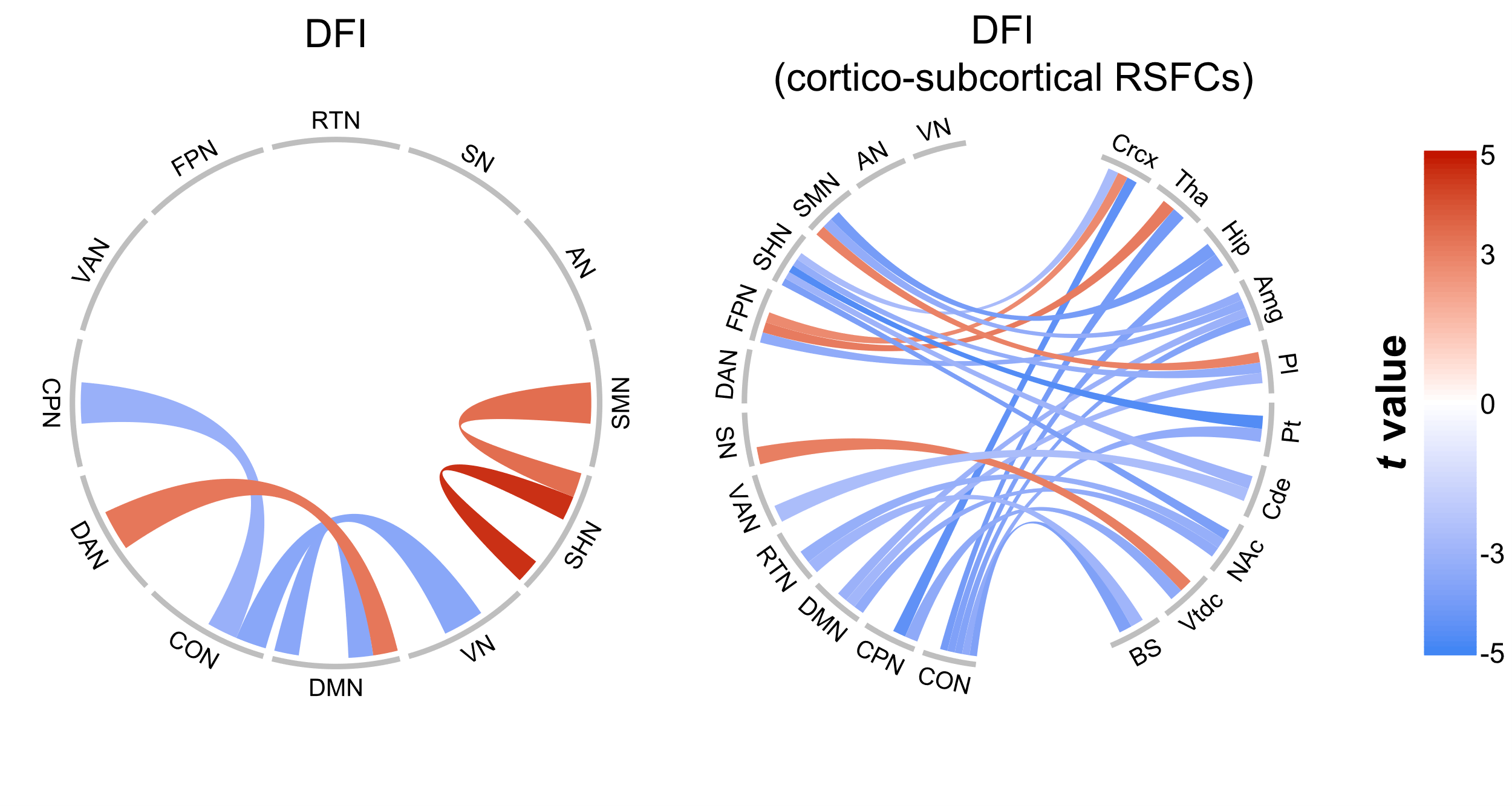


**Supplementary Fig. 8** **| Spatial correlations between *t*-maps of brain volume-peer environments associations and neurotransmitter density (supplementary analyses).** (a-c). Correlations between the *t*-maps (volume, area, thickness) from PFI and neurotransmitter density maps. Consistent with our primary analyses, only the *t*-map (volume) from PFI had one positive association with NAT (*ρ* = 0.42, *p*_fdr_ = 0.031). (d-f). Associations between the *t*-maps (volume, area, thickness) from DFI and neurotransmitter density maps. Similar to our primary analyses, the *t*-map (volume) from DFI still had significant positive associations with 5-HT1b (*ρ* = 0.44, *p*_fdr_ = 0.030), 5-HTT (*ρ* = 0.40, *p*_fdr_ = 0.030), and D1 (*ρ* = 0.41, *p*_fdr_ = 0.030). Moreover, the *t*-map (area) from DFI still had significant positive associations with 5-HTT (*ρ* = 0.56, *p*_fdr_ = 0.001) and DAT (*ρ* = 0.41, *p*_fdr_ = 0.027). Furthermore, the *t*-map (thickness) from DFI still had significant positive associations with 5-HT1b (*ρ* = 0.55, *p*_fdr_ = 0.004), mGluR5 (*ρ* = 0.50, *p*_fdr_ = 0.007), and VAChT (*ρ* = 0.46, *p*_fdr_ = 0.011). FDR corrections were used for multiple comparisons (12 neurotransmitters). * and ** indicate *p*_fdr_ < 0.05, *p*_fdr_ < 0.01, respectively.


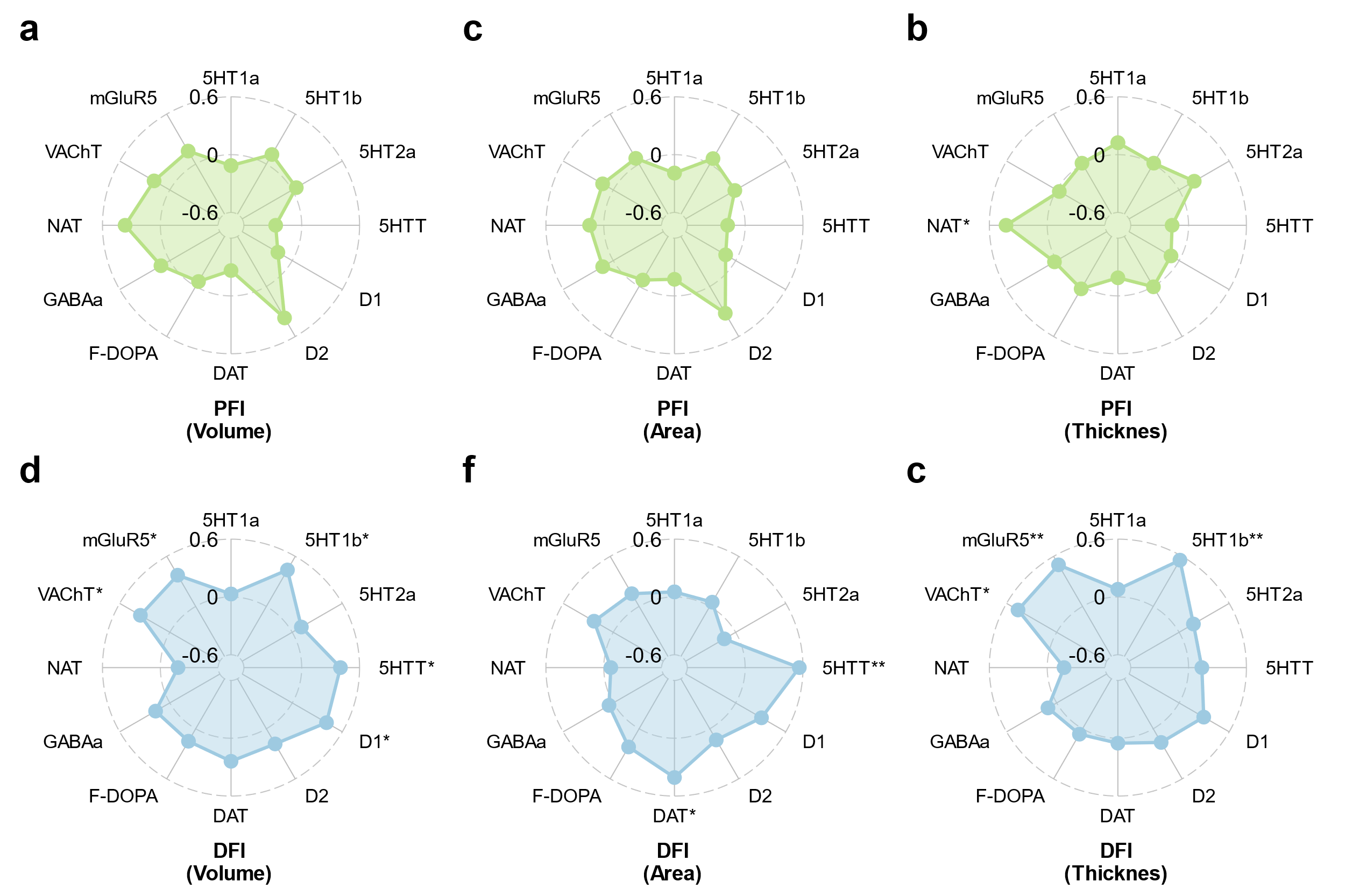


**Supplementary Fig. 9 | The brain features mediated the relationships between peer environments and behaviors (supplementary analyses)**. (a) The mediation model of PFI. M (mediator) = significant brain features, X = PFI, and Y = 57 behavioral measurements (e.g., for brain volumes, FDR correction times = 5 brain volumes × 57 behaviors). (b) The mediation model of DFI. M = DFI, X = significant brain features, and Y = 57 behavioral measurements (e.g., for RSFCs, FDR correction times = 6 RFSCs × 57 behaviors). (c) The relationships between brain features and behaviors were significantly mediated by brain features. For PFI, 19 significant mediation effects (originally 41) were observed for brain volumes. For DFI, 7 significant mediation effects (originally 28) were detected for brain volumes, and 37 significant mediation effects (originally 44) were detected for RSFCs. Abbreviation: VN = visual network; SHN = sensorimotor hand network; SMN = sensorimotor mouth network; CON = cingulo-opercular network; CPN = cingulo-parietal network; DAN = dorsal attention network; DMN = default mode network.


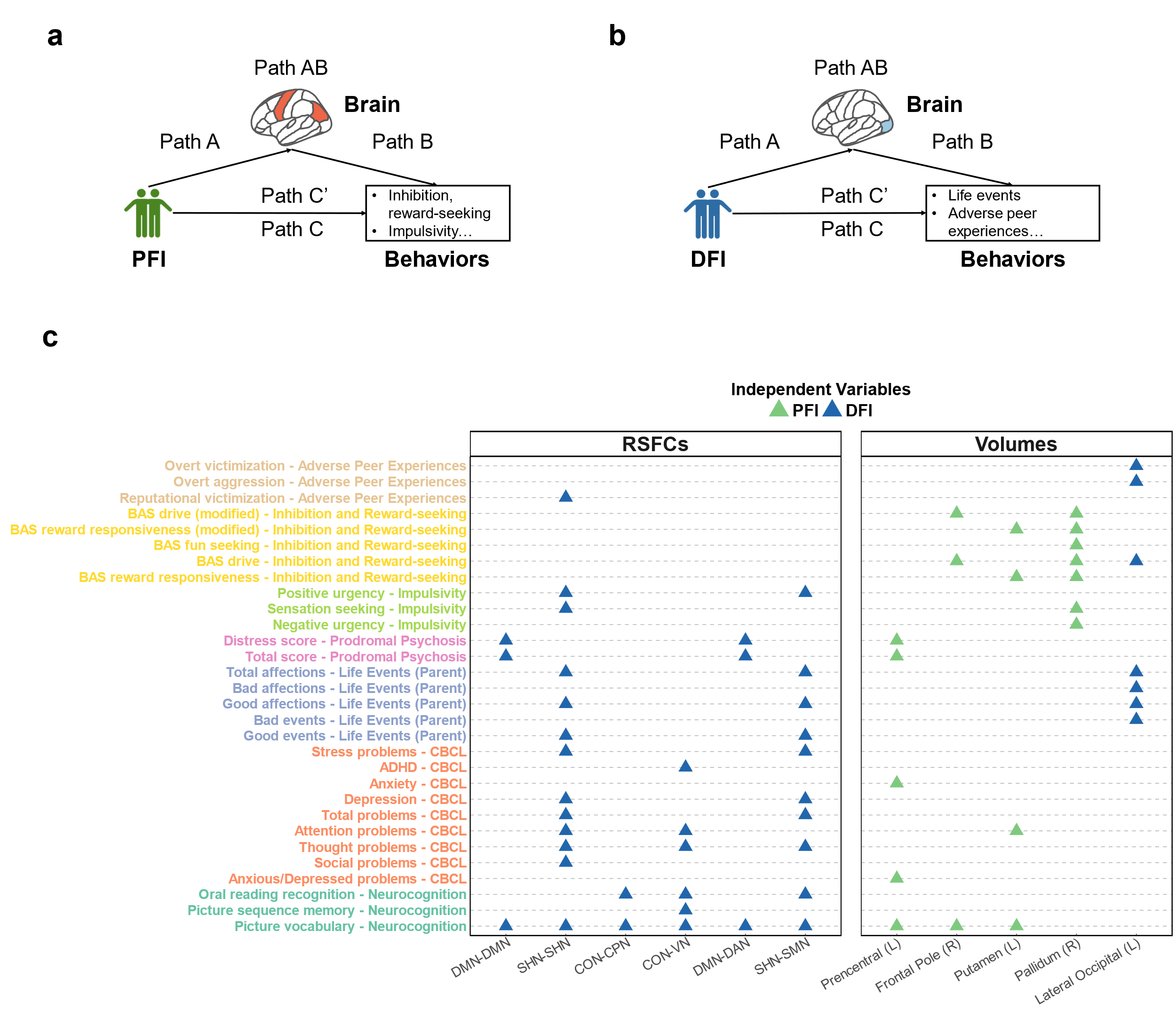


**Supplementary Fig. 10 | The cortico-subcortical RSFCs mediated the relationships between DFI and behaviors (supplementary analyses).** (a) M = brain features (25 cortico-subcortical RSFCs that were significantly associated with DFI), X = DFI, and Y = 57 behavioral measurements. (b) The relationships between DFI and behaviors were mediated by cortico-subcortical RSFCs. A total of 174 (originally 199) significant results were found (*p*_fdr_ < 0.05, FDR correction times = 20 RSFCs × 57 behaviors). Abbreviation: SHN = sensorimotor hand network; SMN = sensorimotor mouth network; CON = cingulo-opercular network; CPN = cingulo-parietal network; DAN = dorsal attention network; DMN = default mode network; FPN = fronto-parietal network; RTN = retrosplenial temporal network; SN = salience network; VAN = ventral attention network.


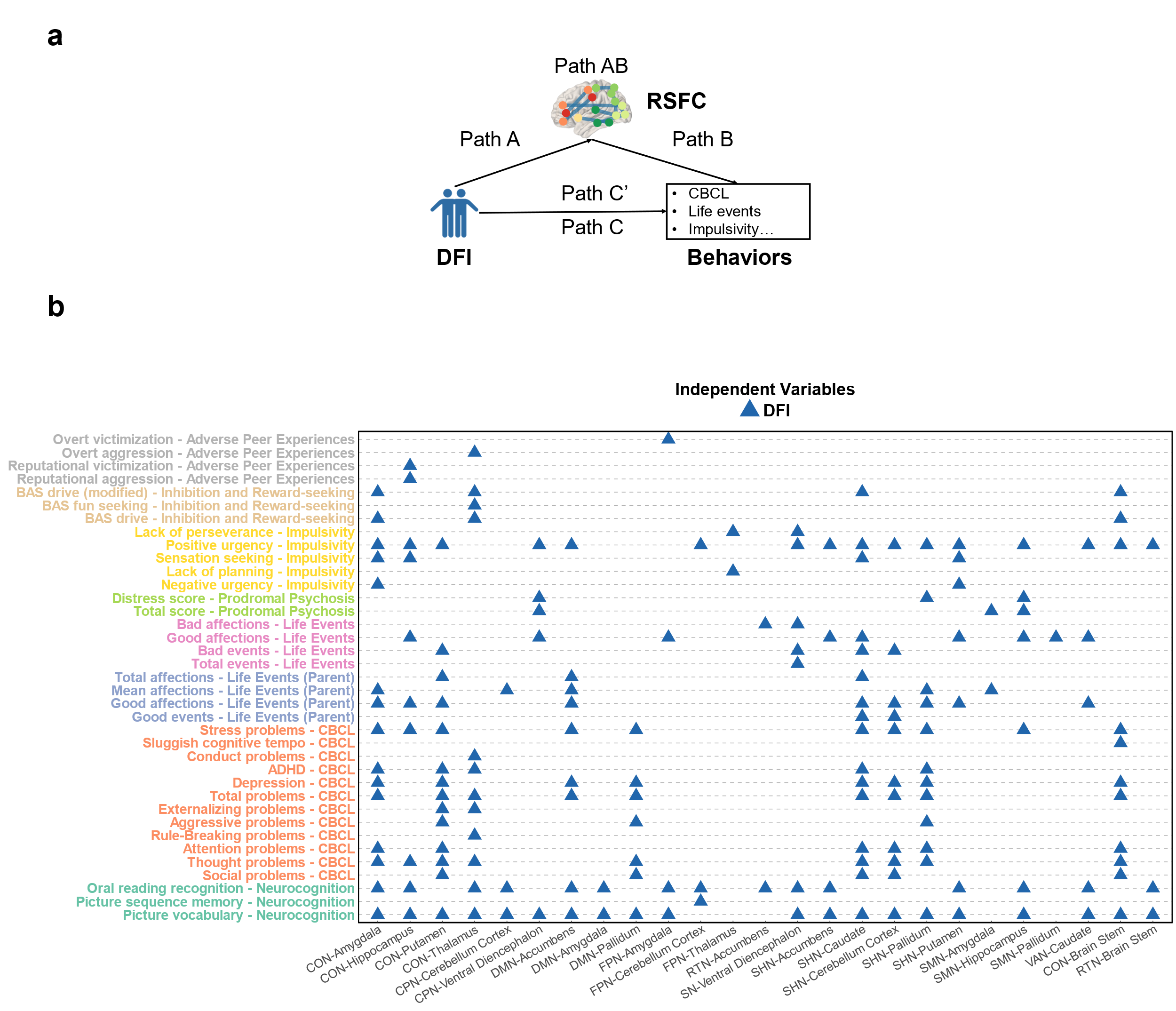


**Supplementary Fig. 11 |** **Longitudinal analysis (supplementary analyses).** Similar to the primary analyses, PFI still had longitudinal associations with internal symptoms, whereas DFI had longitudinal associations with external symptoms and adverse peer experiences. Note that a lack of data on the summary score of neighborhood safety at 3YFU led to their exclusion from the longitudinal analysis involving adolescents at the 3YFU stage. (a) PFI at 2YFU was negatively associated with 12 (originally 19) variables at 3YFU, including depression . (b) DFI at 2YFU positively correlated with 12 (originally 22) variables at 3YFU, including 2 behavioral problem scores (rule-break, conduct), 2 parent-reported life events (bad affections, bad events), 2 life events (bad affections, bad events), 2 prodromal psychosis scores (total score, distress score), and 4 adverse peer experience (reputation aggression, overt victimization, relational aggression and overt aggression). FDR corrections were used for multiple comparisons (*p*_fdr_ < 0.05, *n* = 41 behavioral variables).


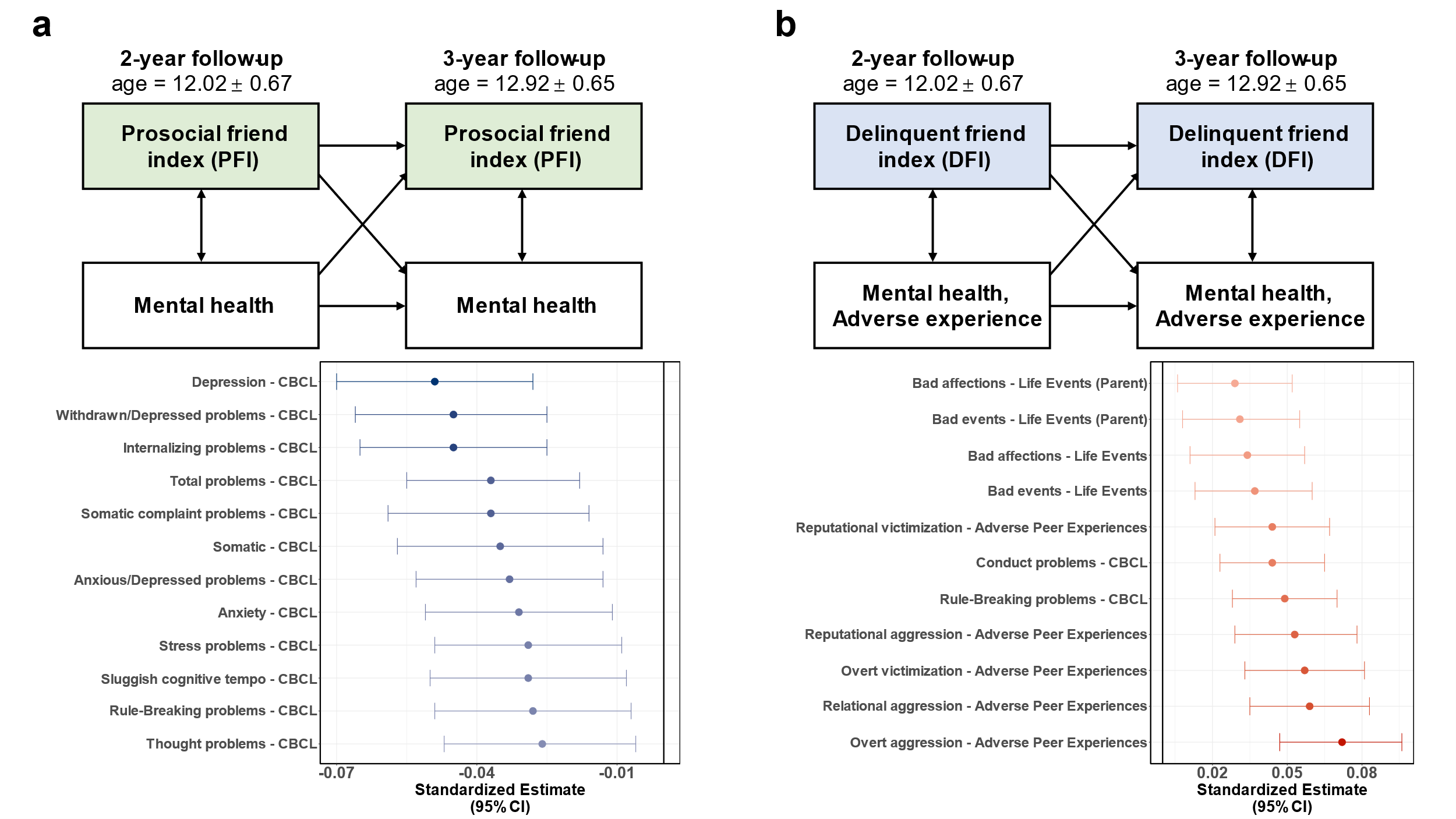


**Supplementary Fig. 12 | PFI’s effect sizes from primary LMMs and 5-fold cross-validation.** The X-axis represents the significant independent variables (behavior, brain, and resting-state functional connectivity) in the association analysis of PFI. High correlations were found between the effect sizes from primary LMMs and 5-fold cross-validation (*r* = 0.998, *p* < 0.001). Note that cross-validation-based R2 *p* is different from R2 s*p*, and is depicted in the figure for better illustration. Abbreviation: AN = auditory network; VN = visual network; SHN = sensorimotor hand network; SMN = sensorimotor mouth network; CON = cingulo-opercular network; CPN = cingulo-parietal network; DAN = dorsal attention network; DMN = default mode network; FPN = fronto-parietal network; RTN = retrosplenial temporal network; SN = salience network; Vtdc = ventral diencephalon; Amg = amygdala; Cde = caudate.


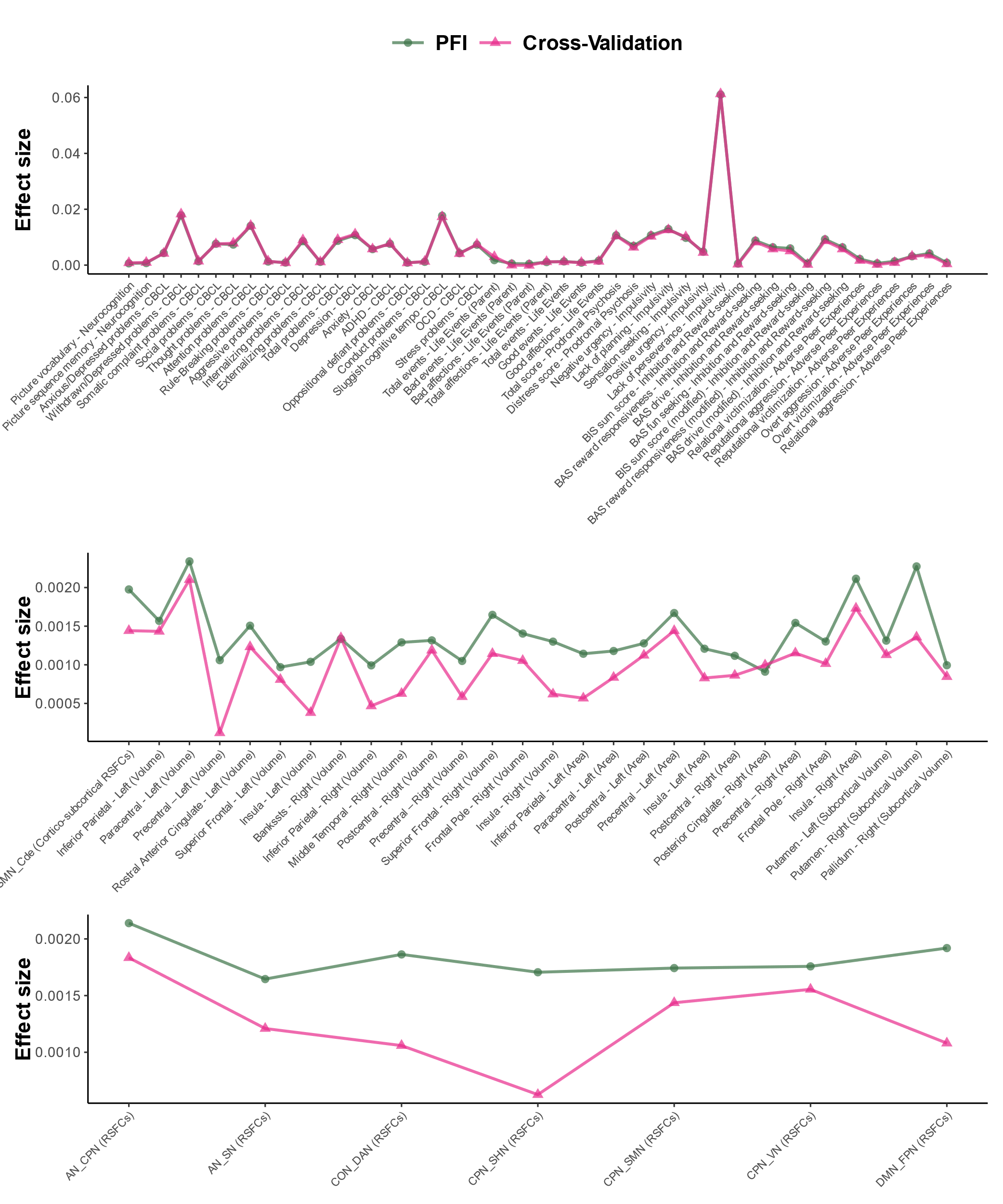


**Supplementary Fig. 13 | DFI’s effect sizes from primary LMMs and 5-fold cross-validation.** The X-axis represents the significant independent variables (behavior, brain, and resting-state functional connectivity) in the association analysis of DFI. High correlations were found between the effect sizes from primary LMMs and 5-fold cross-validation (*r* = 0.994, *p* < 0.001). Note that cross-validation-based R2 *p* is different from R2 s*p*, and is depicted in the figure for better illustration. AN = auditory network; VN = visual network; SHN = sensorimotor hand network; SMN = sensorimotor mouth network; CON = cingulo-opercular network; CPN = cingulo-parietal network; DAN = dorsal attention network; DMN = default mode network; FPN = fronto-parietal network; RTN = retrosplenial temporal network; SN = salience network; VAN = ventral attention network; Crcx = cerebellum cortex; Tha = thalamus; Hip = hippocampus; Amg = amygdala; Pt = putamen; Pl = pallidum; Cde = caudate; NAc = nucleus accumbens; Vtdc = ventral diencephalon; BS = brain-stem.


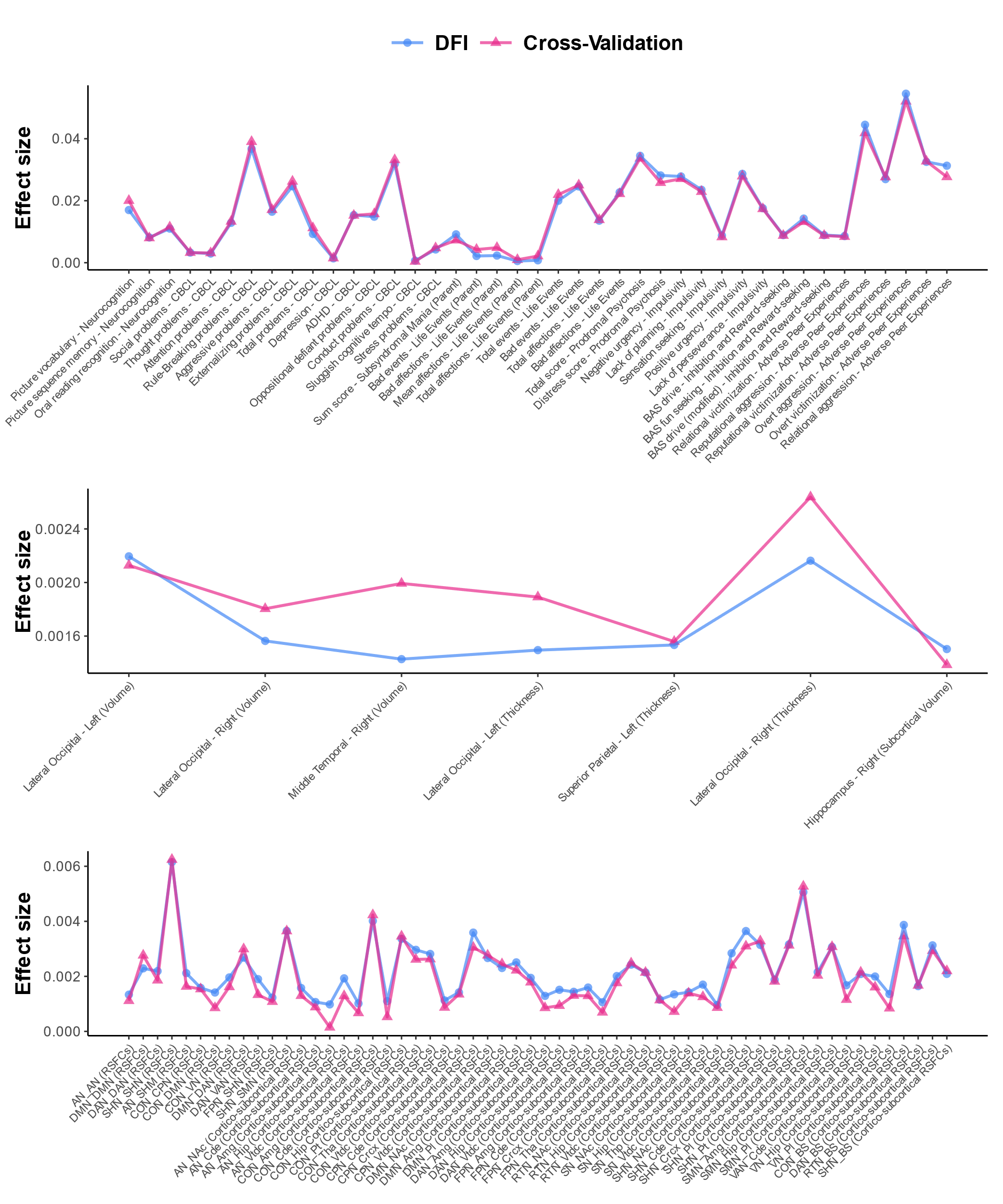


**Supplementary Fig. 14 |** **Validation of neurotransmitter density maps.** Another set of eight available neurotransmitter density maps from other studies was utilized to validate the findings regarding neurotransmitters. The eight validation maps are: 5HT_1a_^2^, 5HT_1b_^2^, 5HT_2a_^2^, 5HTT^2^, D2^3^, GABAa^4^, VAChT^5^, and mGluR5^6^. Overall, these results are similar to our primary analyses. For DFI, *t*-maps still had significant positive spatial correlations with mGluR5 (thickness, *ρ* = 0.43, *p*_fdr_ = 0.049) and VAChT (thickness, *ρ* = 0.37, *p*_fdr_ = 0.048) after the changes of density maps, which suggest the findings from DFI are relatively stable. No significant results were for *t*-maps from PFI (also the same as the primary analyses).


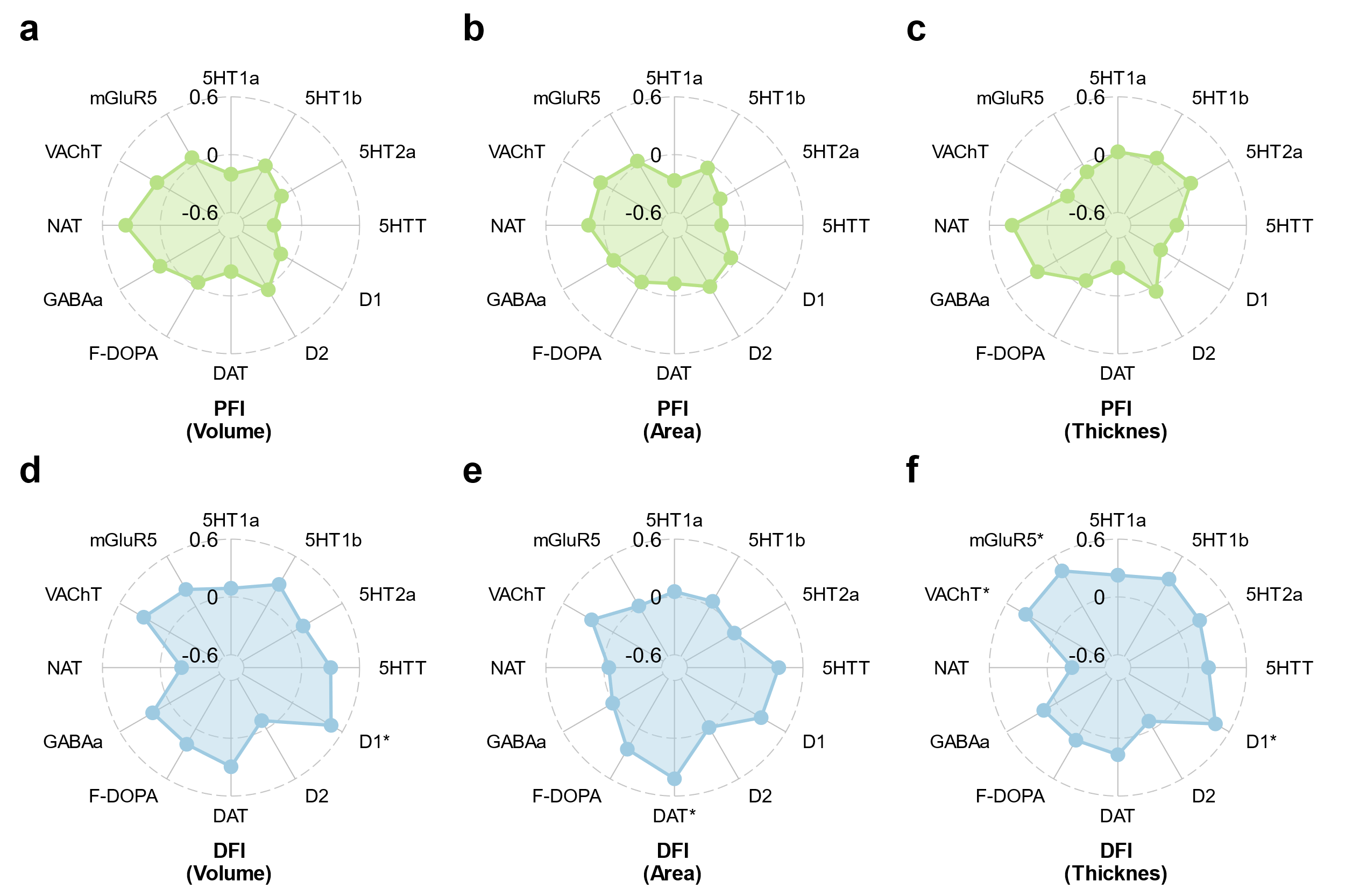


**Supplementary Fig. 15 | Validation of longitudinal analysis (the bivariate latent change score models).** The bivariate latent change score models (BLCS) were used to investigate the longitudinal relationship between peer environments and Δbehavioral variables (changes of behavioral variables between 2-year follow-up and 3-year follow-up). A detailed description of BLCS is available elsewhere^7^. Specifically, BLCS was estimated by using the maximum likelihood algorithm using the R package lavvan^8^. The 2YFU assessments were set for the first time point, and the 3YFU assessments were set as the second time point. Covariates were regressed before running BLCS (the same procedure as the cross-lagged panel model). Overall, similar to our primary analyses, PFI at 2YFU had positive associations with the changes of 19 behavioral or mental variables between 2YFU and 3YFU, including depression and internal problems.

Conversely, DFI at 2YFU had positive associations with the changes of 15 variables between 2YFU and 3YFU, mainly in external problems and adverse peer experiences. FDR corrections were used for multiple comparisons (*p*_fdr_ < 0.05, *n* = 41 behavioral variables).


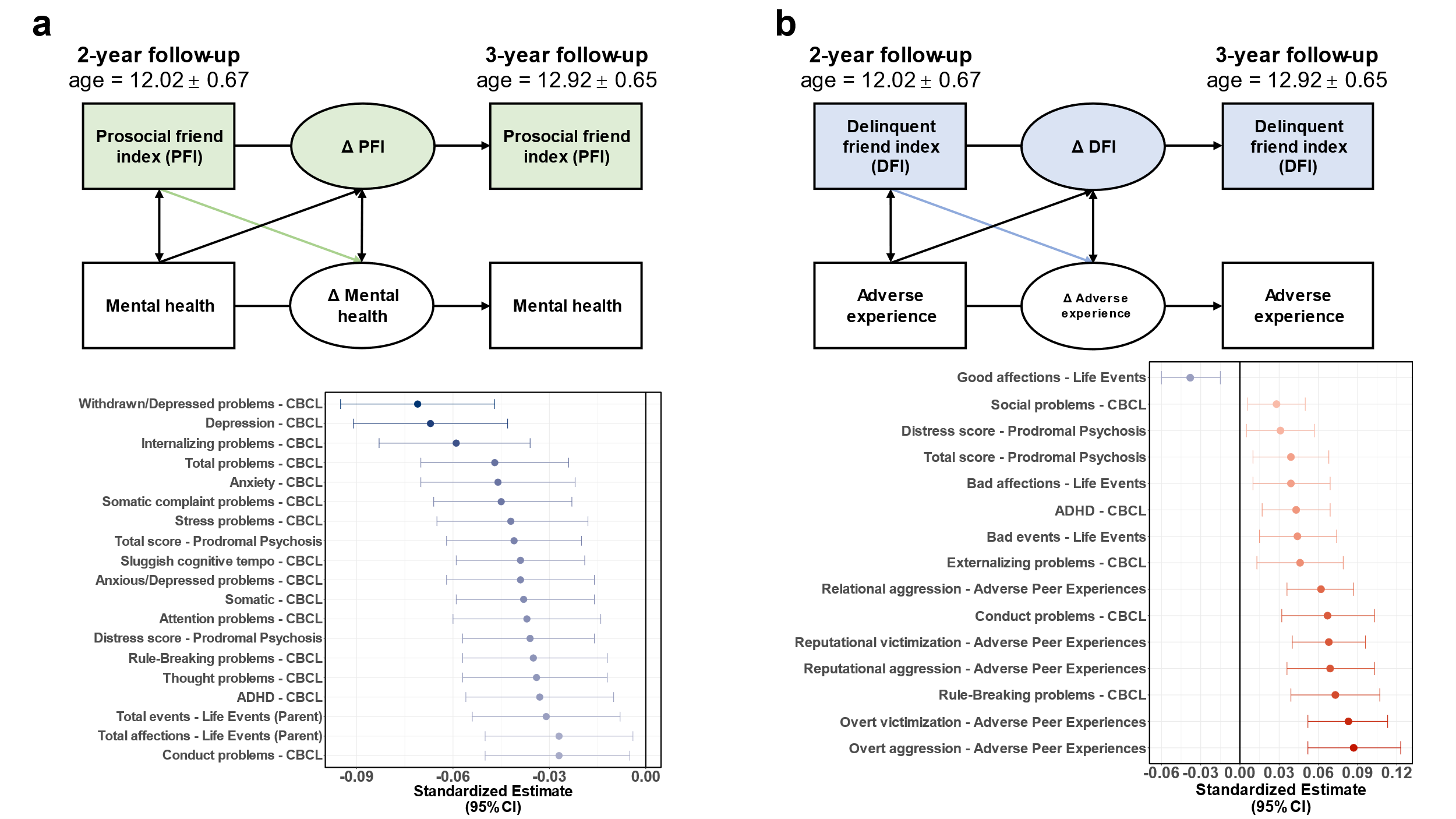


**References**

1 Goltermann, J. *et al.* Cross-validation for the estimation of effect size generalizability in mass-univariate brain-wide association studies. *bioRxiv*, 2023.2003. 2029.534696 (2023).

2 Beliveau, V. *et al.* A High-Resolution In Vivo Atlas of the Human Brain's Serotonin System. *J Neurosci* **37**, 120-128 (2017). <https://doi.org:10.1523/jneurosci.2830-16.2016>

3 Alakurtti, K. *et al.* Long-Term Test–Retest Reliability of Striatal and Extrastriatal Dopamine D2/3 Receptor Binding: Study with [11C]Raclopride and High-Resolution PET. *Journal of Cerebral Blood Flow & Metabolism* **35**, 1199-1205 (2015). <https://doi.org:10.1038/jcbfm.2015.53>

4 Nørgaard, M. *et al.* A high-resolution in vivo atlas of the human brain's benzodiazepine binding site of GABAA receptors. *NeuroImage* **232**, 117878 (2021). <https://doi.org:https://doi.org/10.1016/j.neuroimage.2021.117878>

5 Bedard, M.-A. *et al.* Brain cholinergic alterations in idiopathic REM sleep behaviour disorder: a PET imaging study with 18F-FEOBV. *Sleep Medicine* **58**, 35-41 (2019). <https://doi.org:https://doi.org/10.1016/j.sleep.2018.12.020>

6 DuBois, J. M. *et al.* Characterization of age/sex and the regional distribution of mGluR5 availability in the healthy human brain measured by high-resolution [(11)C]ABP688 PET. *Eur J Nucl Med Mol Imaging* **43**, 152-162 (2016). <https://doi.org:10.1007/s00259-015-3167-6>

7 Kievit, R. A. *et al.* Developmental cognitive neuroscience using latent change score models: A tutorial and applications. *Developmental Cognitive Neuroscience* **33**, 99-117 (2018). <https://doi.org:https://doi.org/10.1016/j.dcn.2017.11.007>

8 Curtis, R., Mackinnon, S. & O'Connor, R. A Tutorial in Longitudinal Measurement Invariance Using Lavaan. (2020).
